# Large-scale survey of a neglected agent of sparganosis *Spirometra erinaceieuropaei* (Cestoda: Diphyllobothriidae) in wild frogs in China

**DOI:** 10.1371/journal.pntd.0008019

**Published:** 2020-02-26

**Authors:** Xi Zhang, Xiu Hong, Shi Nan Liu, Peng Jiang, Shu Chuan Zhao, Chuan Xi Sun, Zhong Quan Wang, Jing Cui

**Affiliations:** Department of Parasitology, School of Basic Medical Sciences, Zhengzhou University, Zhengzhou, China; James Cook University, AUSTRALIA

## Abstract

**Background:**

In China, frogs play an understudied role in the spread of human sparganosis (caused by the larval form of *Spirometra*). However, our knowledge about the prevalence of sparganum infection in frogs remains fragmented, and the taxonomic identification of the parasite is still controversial.

**Methodology/Principal findings:**

The prevalence of sparganum infection in wild frogs was surveyed at 145 geographical locations from 28 of the 34 provinces/autonomous regions/municipalities in China for six years. The collected sparganum isolates from the different locations were subjected to molecular identification by a multiplex PCR assay and then were analysed with clustering analysis. In the survey, sparganum infection was found in 8 out of 13 of the collected frog species, and the most frequently infected species was *Pelophylax nigromaculatus* (the infection rate was up to 14.07%). Infected frogs were found in 80 of the 145 surveyed locations. The sparganum infection rates in the wild frogs in several regions of China were still high (above 10%), especially in South and Southwest China. A total of 72 spargana were selected for molecular identification, and the clustering analysis showed that sequences from the Chinese isolates were very similar to those identified as from *Spirometra erinaceieuropaei*. However, the taxonomy of the genus remains confused and further analysis is required.

**Conclusions:**

Eating wild frogs is associated with considerable health risks in China. Several traditional Chinese folk remedies may increase the risk of infection. The sparganum isolates in China are most likely from *S*. *erinaceieuropaei*, but new studies, especially comprehensive morphological analyses, are needed in the future.

## Author summary

Human sparganosis has increased in recent years in China. Frogs play an important role in the spread of sparganosis. However, our knowledge about the prevalence of sparganum infection in frogs remains incomplete, and the taxonomic identification of sparganum is still controversial. In this study, the prevalence of sparganum infection in frogs was surveyed at 145 geographical locations in China. In addition, 72 spargana representing 72 distinct geographical isolates were selected for molecular identification. Frogs that tested positive for sparganum infection were found in 80 of the 145 surveyed locations, and the average infection rate was 10.96% (447/4078), with most of the spargana present in the thigh muscles of the infected frogs. The levels of sparganum infections in wild frogs in South and Southwest China were higher than those in the central and eastern regions. In the molecular identification analysis, all of the isolates revealed two specific bands in the multiplex PCR assay; the further clustering analysis of the sequenced PCR products showed that the Chinese isolates had a close relationship with *Spirometra erinaceieuropaei*.

## Introduction

Human sparganosis is a neglected food-borne parasitic disease caused by the larval forms (procercoid/plerocercoid) of the species in the genus *Spirometra* [1]. Despite its global distribution, most cases occur in Eastern and Southeastern Asia [2]. Frogs, as the second intermediate host in the life cycle of *Spirometra*, play an important role in the spread of the disease in China [3, 4]. Humans can be infected through the consumption of raw or undercooked frog meat or by using raw frog flesh in traditional poultices [5]. In addition, eating raw frog meat is a traditional custom for many people in some areas of China [6–8]. Recently, more than 30 autochthonous human cases caused by the ingestion of live frog tadpoles have been recorded in central China [4]. The number of reported cases of human sparganosis has exceeded 1300 in China [3, 9], but this number is believed to be an underestimate. The actual number of infections may be far higher than those estimated because many cases may not be recognized or reported. As a result, the investigation of infection by plerocercoid larva (sparganum) in frogs is therefore valuable for food safety and the prevention and control of human sparganosis in China [10].

The human sparganosis has been reported to be distributed in 26 of 34 provinces/autonomous regions/municipalities in China, with the majority of cases in Southern and Eastern China [3]. Sporadic investigations of the sparganum infection in frogs have been performed in several regions of China, such as in Henan, Hunan and Guangdong provinces [6, 11–13]; however, there is still a lack of data on the geographical distribution of sparganum infection in wild frogs in all regions of China where the parasite is endemic. In this study, we conducted the first large-scale survey of sparganum infection in wild frogs from 145 geographical locations that covered 88.9% of the endemic regions of human sparganosis.

Although a medically important genus, the identification and taxonomy of *Spirometra* species have been controversial for a long time [14]. The most recent review concluded that there were only 4 valid species (*S*. *erinaceieuropaei*, *S*. *mansonoides*, *S*. *pretoriensis* and *S*. *theileri*) in the genus *Spirometra* [15]; however, dozens of nominal species of *Spirometra* have been described in other publications [16–18]. In addition, several unidentified species have been reported in South America [19]. Using different genetic markers or the complete mitochondrial genome, *Spirometra* isolates from Australia, New Zealand, Indonesia, India, Japan and Korea [20], mainland China [6, 10, 12–14, 21, 22] and Hong Kong [23] have been assigned as *S*. *erinaceieuropaei*. In contrast, one isolate from Korea was identified as *S*. *decipiens* (KJ599679) through both morphological and genetic methods [24]. However, the morphological identification of the Korean *S*. *decipiens* did not have detailed evidence, such as the study of the type material of *S*. *decipiens* or collection of new material from the type locality, and two recent phylogenetic analyses have suggested that the Korean *S*. *decipiens* was likely conspecific with the Asian isolates of *S*. *erinaceieuropaei* [14, 19], so we refer to the Korean *S*. *decipiens* as the “Korean *S*. *decipiens* genotype” here for careful consideration. Another Korean isolate, *S*. *erinaceieuropaei* (KJ599680) (which we call the “Korean *S*. *erinaceieuropaei* genotype”), was genetically more distinct from other Asian isolates of *S*. *erinaceieuropaei* [14, 19]. In China, most reported cases of human sparganosis have been caused by *S*. *erinaceieuropaei*. In contrast, both the “Korean *S*. *erinaceieuropaei* genotype” and “Korean *S*. *decipiens* genotype” have been identified as infectious to humans [24]. In addition, several sparganum isolates collected from snakes (*Dinodon rufozonatum* and *Agkistrodon saxatilis*) in China were identified as the “Korean *S*. *decipiens* genotype” [17]. Therefore, the exact identification of *Spirometra* species requires further investigation.

To clarify the exact taxonomy of the collected sparganum isolates and to explore whether there were other species of *Spirometra* in China, we intended to identify the collected samples using the multiplex PCR assay developed by Jeon et al. [18]. The multiplex PCR method was useful for distinguishing the “Korean *S*. *erinaceieuropaei* genotype” and “Korean *S*. *decipiens* genotype” [18]. More specifically, the aims of this study were as follows: (1) to conduct a large-scale survey of sparganum infections in wild frogs in mainland China; and (2) to identify the collected sparganum specimens using molecular identification.

## Materials and methods

### Ethics statement

Our study was performed strictly based on the recommendations of the Guide for the Care and Use of Laboratory Animals of the National Health Commission of China. The protocol was approved by the Life Science Ethics Committee of Zhengzhou University (Permission No. 2013–0137). All of the frog specimens were collected from paddy fields after obtaining the permission of farm owners. No specific permits were required by the authorities for the collection of frog samples.

### Study site

**Fig 1:**
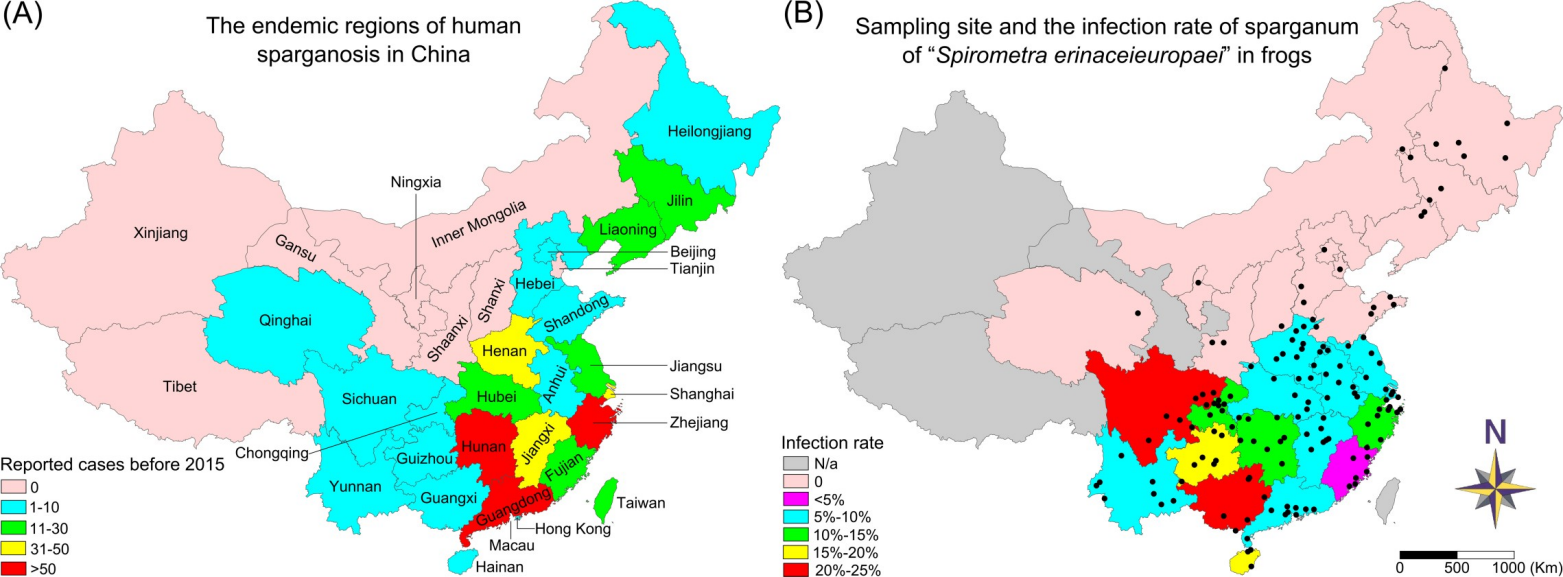
The sampling sites of wild frogs and the prevalence of *Spirometra* sparganum infection in frogs in each province/autonomous region/municipality of China. (A) The endemic regions of human sparganosis and reported cases before 2015 in each province/autonomous region/municipality of China. (B) The sampling sites and the infection rate of *Spirometra erinaceieuropaei* sparganum in frogs in each province/autonomous region/municipality of China.

According to the most recent review of human sparganosis in China [3], the endemic region for the disease encompassed 26 provinces/autonomous regions/municipalities (Fig 1A). More specifically, there were 2 regions in North China: Hebei (HeB) province and Beijing (BJ) municipality; 3 regions in Northeast China: the Heilongjiang (HLJ), Jilin (JL) and Liaoning (LN) provinces; 8 regions in East China: the Shandong (SD), Anhui (AH), Jiangsu (JS), Zhejiang (ZJ), Jiangxi (JX), Fujian (FJ), and Taiwan (TW) provinces and Shanghai (SH) municipality; 3 regions in central China: the Henan (HeN), Hubei (HuB) and Hunan (HuN) provinces; 5 regions in South China: Guangxi (GX) autonomous region and the Guangdong (GD) and Hainan (HN) provinces and Hong Kong and Macao; 4 regions in Southwest China: the Sichuan (SC), Yunnan (YN), and Guizhou (GZ) provinces and Chongqing (CQ) municipality; and only 1 region in Northwest China: Qinghai (QH) Province. From July 2013 to September 2018, we surveyed wild frogs for sparganum infections in 23 of these 26 endemic regions (excluding Taiwan, Hong Kong and Macao). In addition, 5 nonendemic regions were also investigated in our survey: Tianjin (TJ) municipality, the Shaanxi (SaX) and Shanxi (SX) provinces and the Ningxia (NX) and Inner Mongolia (IM) autonomous regions. In total, 145 geographical sampling sites in 28 of the 34 administrative regions in China were included (Fig 1B). The precise locality (coordinates), origin and date of collection for each sampling site are presented in the Supplementary S1 Table.

### Examination of frogs and sample collection

The presence of spargana in wild frogs was evaluated according to previously described methods [12]. Briefly, the frogs were caught in paddy fields or other wild environments, and then the collected frogs were euthanized using ethyl-ether anaesthesia and were weighed and skinned. The presence of spargana in the skeletal muscles was carefully observed visually. Once identified, the spargana were isolated using small scissors and forceps (Supplementary S1 Fig). The white spargana are highly active and able to change shape in physiological saline. The larvae show an enlarged head and many horizontal folds on the surface of the body when examined under a microscope. The number of identified spargana, parasitizing sites, infection intensity and length of each sparganum were counted and measured. The measurements were expressed as ranges, with the means followed by SDs. After that, all collected larvae were preserved in alcohol (99% ethanol) for molecular identification.

### Multiplex PCR and sequencing analysis

The multiplex PCR method was used to identify the sparganum isolates collected from the different geographical locations. For each specific locality, one sparganum was selected for the multiplex PCR assay. In total, 72 spargana were used for the molecular identification, and specimens from 8 locations were excluded because of the close proximities of the sampling sites (Supplementary S2 Table). The multiplex PCR assay was performed using two sets of species-specific primers (Supplementary S3 Table) and the methods described in Jeon et al. [18]. In brief, two sets of species-specific primers, Se/Sd-1800F + Se-2018R plus Se/Sd-7955F + Se-8356R, and Se/Sd-1800F + Sd-2317R plus Se/Sd-7955F + Sd-8567R, were designed based on the mitochondrial nucleotide sequences of cytochrome *b* (*cyt*b), NADH dehydrogenase subunit 4L (*nad*4L), NADH dehydrogenase subunit 4 (*nad*4), cytochrome c oxidase subunit 1 (*cox*1), and large subunit ribosomal DNA (lrDNA) from “*S*. *erinaceieuropaei* (KJ599680)” and “*S*. *decipiens* (KJ599679)”. As described, using a combination of “Korean *S*. *erinaceieuropaei* genotype” specific primers (Se/Sd-1800F + Se-2018R; Se/Sd-7955F + Se-8356R) should generate two “*S*. *erinaceieuropaei*” specific bands of 239 bp and 401 bp, respectively. In contrast, the “Korean *S*. *decipiens* genotype” specific primers (Se/Sd-1800F + Sd-2317R; Se/Sd-7955F + Sd-8567R) should generate two “*S*. *decipiens*” specific bands of 540 bp and 644 bp, respectively. In addition, the amplified PCR products were sequenced to confirm the results of the electrophoretic analysis. The PCR products were purified using the EasyPure PCR Purification Kit (Transgen, China) and sequenced bidirectionally using an automated sequencer (ABI Prism 3730 XL DNA Analyzer; ABI Prism, Foster City, CA) at the Genwiz Company (Suzhou, China). All sequences were deposited in the GenBank database under accession numbers MN842190-MN842261 and MN861812-MN861883. Moreover, eight complete mitochondrial genomes of *S*. *erinaceieuropaei* (JQ267473, KY114886-114889, AP017668, AB374543 and KU852381), two mitochondrial genomes from the Korean genotypes, the “*S*. *decipiens* genotype” (KJ599679) and “*S*. *erinaceieuropaei* genotype” (KJ599680), from GenBank were included as reference sequences. One *Schistocephalus* species (*Schistocephalus solidus*), two Solenophoridae species (*Duthiersia expansa* and *Scyphocephalus bisulcatus*) and one Cephalochlamydidae species (*Cephalochlamys namaquensis*), were used as outgroups. These species were selected as the closest known groups to *Spirometra* based on the study of Waeschenbach et al. [25]. The sequences were aligned in MEGA v.6.06 [26] with the default settings. The variable sites, nucleotide compositions and pairwise distances were also estimated in MEGA. The clustering analysis was performed with two methods: the maximum likelihood (ML) method and Bayesian inference (BI). The substitution model of the dataset was selected by jModelTest v0.2 [27]. The ML analysis was conducted in MEGA, and the confidence levels in each node were assessed with the boot-strap method (1000 pseudoreplicates). The BI analysis was performed in MrBayes v.3.2 [28]. The analysis consisted of two runs, each with four MCMC chains running for 5,000,000 generations, which were sampled every 100th generation.

## Results

### Prevalence of sparganum infection in frogs in China

From July 2013 to September 2018, the prevalence of sparganum infection in wild frogs was surveyed at 145 geographical locations in 28 of the 34 provinces/autonomous regions/municipalities in China (Fig 1). A total of 4665 wild frogs belonging to 13 species were collected (Table 1). Among these 13 frog species, the 5 species *Rana chensinensis*, *Microhyla heymonsi*, *Polypedates megacephalus*, *Kaloula pulchra* and *Hyla chinensis* did not show sparganum infection, indicating that these frog species were insensitive for sparganum infection. The remaining 8 species were susceptible to sparganum infection. In general, *Spirometra* spargana were found in 10.96% (447/4078) of these 8 positive frog species. The highest infection rate was found in *P*. *nigromaculatus* (14.07%), followed by *O*. *margaretae* (13.30%) and *S*. *latouchii* (12.15%). Relatively low infection rates were found in *Q*. *spinosa* (2.78%) and *P*. *plancyi* (3.51%). Interestingly, the highest mean infection intensity of sparganum in the infected frogs was also found in *P*. *nigromaculatus* (4.27±3.02), followed by *O*. *margaretae* (4.04±1.37) and *S*. *latouchii* (4.01±3.61), and the mean infection intensity in *Q*. *spinosa* was the lowest (2.25±1.89). For the mean length of the spargana, the longest was identified in *H*. *chinensis* (6.09±1.84 cm), and the shortest was in *P*. *nigromaculatus* (4.68±2.13 cm). The prevalence of sparganum infection in wild frogs ranged from 0 to 66.67%, with an infection intensity of 1–49 spargana per frog in different geographical locations (Table 2). Most of the spargana were present in the thigh muscles of the frogs (Supplementary S4 Table). The percentage of the parasitic spargana that were found in the thighs was 68.11% (1085/1593), followed by the backside (14.94%, 238/1593) and abdomen (9.98%, 159/1593). In the endemic regions, the provinces with the highest numbers of human cases were Hunan, Zhejiang and Guangdong (>50 cases), and the corresponding sparganum infection rates in wild frogs were 14.29% (31/217), 10.86% (33/304) and 7.73% (17/220), respectively (Fig 1 and Supplementary S4 Table). The reported human cases in Jiangxi, Shanghai and Henan were the second highest (31–50 cases), and the infection rates in these regions were 9.01% (20/222), 8.82% (6/68) and 8.79% (88/1001), respectively. In the regions with moderate number of human cases (11–30 cases), which included Jiangsu, Hubei and Fujian, the infection rates were 9.14% (17/186), 8.56% (16/187) and 3.9% (8/205), respectively. In the regions with low number of reported human cases (1–10 cases), the frogs collected from Heilongjiang (0/178), Hebei (0/18), Beijing (0/13), Shandong (0/90) and Qinghai (0/7) were negative for sparganum infection. In addition, no sparganum-positive frogs were found in Jilin (0/69) and Liaoning (0/68) in Northeast China. The infection rates in the Anhui (6.61%, 22/333) and Yunnan (6.81%, 16/235) provinces were also low. However, the infection rates in most of the southern and southwestern regions, such as Chongqing (10.23%, 18/176), Guizhou (15.24%, 16/105), Hainan (15.49%, 11/71), and especially Sichuan (23.61%, 51/216) and Guangxi (24.84%, 77/310), were high. In the non-endemic regions of Inner Mongolia (0/20), Tianjin (0/16), Shanxi (0/25), Shaanxi (0/42) and Ningxia (0/31), the frogs were negative for infection. In general, the levels of sparganum infection in frogs in the South and Southwest China were higher than those in central and East China. However, no infected frogs have been found in Northeast China in the past 6 years.

**Table 1:** The comparison of sparganum infections in different frog species collected in different geographical locations in China. N = number of frogs examined, P = number of positive frogs, MI = mean infection intensity, ML = mean length of spargana. Geographic regions in China are designated as described in the “Study site” section of the main text.

Frog species	N	P (P/N)	MI	ML (cm)	Geographical origin (N)
*Pelophylax nigromaculatus*	1642	231 (14.07)	4.27±3.02	4.68±2.13	IM (4), HLJ (22), LN (19), HeB (6), JL (5), BJ (4), TJ (4), SX (8), SD (22), AH (111), JS (31), ZJ (74), JX (47), FJ (31), SH (40), HeN (587), HuB (43), HuN (89), GD (73), GX (133), SC (92), GZ (41), CQ (88), QH (7), SaX (30), NX (31)
*Pelophylax plancyi*	456	16 (3.51)	3.30±2.49	5.06±1.84	HLJ (18), LN (35), HeB (8), BJ (9), TJ (11), SX (17), SD (42), AH (79), JS (39), ZJ (65), JX (53), SH (13), HeN (67)
*Fejervarya limnocharis*	736	75 (10.19)	2.49±1.79	5.12±2.19	HeB (4), TJ (1), SD (26), AH (62), JS (34), ZJ (52), JX (33), FJ (16), SH (8), HeN (223), HuB (18), HuN (28), GD (23), GX (17), HaN (18), SC (36), YN (62), GZ (10), CQ (53), SaX (12)
*Sylvirana latouchii*	494	60 (12.15)	4.01±3.61	5.88±2.36	AH (68), JS (27), ZJ (41), JX (38), FJ (56), SH (4), HeN (56), HuB (43), HuN (35), GD (48), GX (53), GZ (25)
*Boulengerana guentheri*	397	34 (8.56)	2.28±0.87	5.69±1.75	AH (26), JS (23), ZJ (35), JX (31), FJ (32), HeN (67), HuB (22), HuN (22), GD (18), GX (20), HaN (16), SC (25), YN (57), GZ (3)
*Quasipaa spinosa*	144	4 (2.78)	2.25±1.89	4.96±1.35	AH (13), JS (24), ZJ (16), JX (10), FJ (20), SH (3), HuB (15), HuN (19), GD (7), GX (17)
*Odorrana margaretae*	188	25 (13.30)	4.04±1.37	5.41±1.97	HuB (38), HuN (12), GD (4), GX (25), SC (48), GZ (26), CQ (35)
*Hoplobatrachus chinensis*	21	2 (9.52)	4.00±1.41	6.09±1.84	AH (2), JS (1), ZJ (2), FJ (2), HeN (1), GX (3), HaN (5), YN (5)
*Rana chensinensis*	232	0	0	0	IM (16), HLJ (138), LN (14), JL (64)
*Microhyla heymonsi*	107	0	0	0	AH (4), JS (5), ZJ (7), JX (3), FJ (5), HuB (6), HuN (9), GD (3), GX (17), HaN (14), SC (7), YN (27)
*Polypedates megacephalus*	51	0	0	0	JS (2), ZJ (3), FJ (6), HuB (2), HuN (3), GD (3), GX (4), HaN (8), SC (8), YN (12)
*Kaloula pulchra*	128	0	0	0	FJ (22), GD (29), GX (21), HaN (10), YN (46)
*Hyla chinensis*	69	0	0	0	ZJ (9), JX (7), FJ (15), GD (12), YN (26)
**Total or Average**	4665	447 (9.58)	3.31±2.43	5.30±1.97	

**Table 2:** Prevalence of sparganum of *Spirometra erinaceieuropaei* infection in frogs in China during 2013–2018.

Origin		Locality		No. infected/No. examined (%)	Infection intensity (spargana/frog)
Province/autonomous region/municipality	County	Longitude	Latitude		
Inner Mongolia (IM)	Wulanhaote, Xinganmeng	122.23E	46.03N	0/20 (0)	0
Hebei (HeB)	Qiaodong, Xingtai	114.51E	37.07N	0/10 (0)	0
	Shijiazhuang	114.26E	38.03N	0/8 (0)	0
Beijing (BJ)	Yanqing	116.14E	40.31N	0/13 (0)	0
Tianjin (TJ)	Dongli	117.31E	39.09N	0/16 (0)	0
Shanxi (SX)	Jincheng	112.83E	35.52N	0/25 (0)	0
Heilongjiang (HLJ)	Qitaihe	130.49E	45.48N	0/22 (0)	0
	Qinggang, Suihua	126.59E	46.38N	0/36 (0)	0
	Daqing	125.01E	46.36N	0/7 (0)	0
	Acheng, Harbin	126.96E	45.55N	0/31 (0)	0
	Baoquanling, Hegang	130.53E	47.43N	0/40 (0)	0
	Daxinganling	125.47E	50.10N	0/42 (0)	0
Jilin (JL)	Changchun	125.32E	43.82N	0/24 (0)	0
	Baicheng	122.84E	45.62N	0/33 (0)	0
	Siping	124.37E	43.17N	0/12 (0)	0
Liaoning (LN)	Kaiyuan, Tieling	124.04E	42.55N	0/40 (0)	0
	Tieling	123.84E	42.29N	0/28 (0)	0
Shandong (SD)	Yantai	121.39E	37.50N	0/27 (0)	0
	Juancheng, Heze	115.51E	35.56N	0/20 (0)	0
	Rushan, Weihai	121.54E	36.92N	0/13 (0)	0
	Gaomi, Weifang	119.76E	36.38N	0/21 (0)	0
	Pingdu, Qingdao	119.99E	36.78N	0/9 (0)	0
Anhui (AH)	Yizhou, Yicheng	118.75E	30.95N	2/43 (4.65)	1
	Lujiang, Hefei	117.25E	31.88N	6/89 (6.74)	1–5
	Wuwei, Wuhu	118.57E	31.15N	1/52 (1.92)	5
	Linquan, Fuyang	115.26E	33.04N	0/26 (0)	0
	Huoqiu, Luan	116.28E	32.35N	9/40 (22.5)	1–8
	Yuexi, Anqing	116.36E	30.85N	0/32 (0)	0
	Dangtu, Maanshan	118.50E	31.57N	1/32 (3.13)	2
	Qiaocheng, Bozhou	115.78E	33.88N	0/28 (0)	0
	Bengbu	117.36E	32.94N	3/23 (13.04)	1–2
Jiangsu (JS)	Kunshan, Suzhou	120.98E	31.38N	6/35 (17.14)	1–3
	Pizhou, Xuzhou	118.01E	34.34N	0/35 (0)	0
	Runzhou, Zhenjiang	119.41E	32.20N	8/32 (25)	2–8
	Funing, Yancheng	119.80E	33.78N	2/35 (5.17)	1–2
	Dafeng, Yancheng	120.50E	33.20N	1/30 (3.33)	1
	Ganyu, Lianyungang	119.17E	34.84N	0/19 (0)	0
Zhejiang (ZJ)	Pinghu, Jiaxing	121.02E	30.70N	1/39 (2.56)	1
	Yuyao, Ningbo	121.15E	30.03N	4/36 (11.11)	2–6
	Beilun, Ningbo	121.85E	29.93N	3/31(9.68)	1
	Cixi, Ningbo	121.23E	30.17N	7/50 (14)	1–19
	Shaoxing	120.47E	30.08N	7/58 (12.07)	1–2
	Wucheng, Jinhua	119.57E	29.09N	0/7 (0)	0
	Dinghai, Zhoushan	122.11E	30.02N	0/23 (0)	0
	Ouhai, Wenzhou	120.61E	27.97N	1/20 (5)	3
	Dongbaihu, Zhuji	120.38E	29.58N	10/40 (25)	1–7
Jiangxi (JX)	Linchuan, Fuzhou	116.31E	27.93N	3/14 (21.43)	1–8
	Chonggang, Fuzhou	116.38E	27.90N	6/53 (11.32)	1–5
	Chongren, Fuzhou	116.06E	27.77N	3/43 (6.98)	1–5
	Xingzi, Jiujiang	116.05E	29.45N	1/27 (3.7)	1
	Jishui, Jian	115.14E	27.23N	3/40 (7.5)	1–2
	Yushui, Xinyu	115.14E	27.23N	0/4 (0)	0
	Yifeng, Yichun	114.80E	28.39N	4/31 (12.9)	1–6
	Xinjian, Nanchang	115.82E	28.69N	0/10 (0)	0
Fujian (FJ)	Xianyou, Putian	118.69E	25.36N	0/44 (0)	0
	Tongan, Xiamen	118.15E	24.72N	0/5 (0)	0
	Shouning, Ningde	119.51E	27.45N	1/40 (2.5)	1
	Pingtan, Fuzhou	119.79E	25.50N	0/20 (0)	0
	Quanzhou	118.68E	24.87N	4/38 (4.53)	1–3
	Yanghou, Nanping	118.52E	26.63N	3/46 (6.52)	3–7
	Haikou, Fuqing	119.47E	25.70N	0/12 (0)	0
Shanghai (SH)	Nanhui	121.85E	30.86N	5/43 (11.63)	1–9
	Huangpu	121.48E	31.23N	1/13 (7.69)	2
	Songjiang	121.45E	31.03N	0/12 (0)	0
Henan (HeN)	Zhengzhou	113.65E	34.73N	19/161 (11.8)	1–16
	Nanle, Puyang	115.20E	36.07N	0/20 (0)	0
	Nanzhao, Nanyang	112.43E	33.49N	5/47 (10.65)	2–5
	Lushan, Pingdingshan	112.91E	33.74N	0/21 (0)	0
	Shihe, Xinyang	114.06E	32.10N	2/33 (6.06)	1–2
	Huangchuan, Xinyang	115.05E	32.13N	1/15 (6.67)	2
	Yongcheng	116.45E	33.93N	0/20 (0)	0
	Xiayi, Shangqiu	116.13E	34.24N	0/17 (0)	0
	Hua, Anyang	114.52E	35.58N	0/22 (0)	0
	Xinxiang	113.87E	35.30N	4/67 (5.97)	1–4
	Kaifeng	114.47E	34.48N	14/153 (9.15)	1–17
	Fugou, Zhoukou	114.38E	34.07N	16/142 (11.27)	1–13
	Luohe	114.02E	33.58N	27/283 (9.54)	1–20
Hubei (HuB)	Yunmeng, Xiaogan	113.75E	31.02N	7/46 (15.22)	1–12
	Xiangzhou, Xiangyang	112.21E	32.09N	0/18 (0)	0
	Chongyang, Xianning	114.04E	29.56N	1/5 (20)	2
	Yunxi, Shiyan	110.43E	32.99N	0/18 (0)	0
	Huanggang	114.88E	30.45N	8/43(18.6)	1–2
	Laifeng, Enshi	109.41E	29.49N	0/27 (0)	0
	Mingshan, Daye	114.76E	30.07N	0/30 (0)	0
Hunan (HuN)	Sangzhi, Zhangjiajie	110.20E	29.41N	4/57 (7.02)	1–49
	Xupu, Huaihua	110.59E	27.91N	3/12 (25)	2–4
	Shaodong, Shaoyang	111.74E	27.26N	2/19 (10.53)	5–12
	Huarong, Yueyang	112.54E	29.53N	4/29 (13.79)	1–6
	Leiyang, Hengyang	112.83E	26.31N	5/40 (12.5)	1–2
	Yunhuqiao, Xiangtan	112.73E	27.85N	2/10 (20)	1–5
	Fenghuang, Xiangxi	109.58E	27.96N	10/43 (23.26)	1–7
	Changsha	113.04E	28.14N	1/7 (14.29)	1
Guangdong (GD)	Chashan, Dongguan	113.87E	23.08N	3/20 (15)	2–8
	Shunde, Fushan	113.29E	22.81N	11/45 (24.44)	1–9
	Guangzhou	113.26E	23.13N	1/18 (5.56)	1
	Baoan, Shenzhen	113.88E	22.56N	0/5 (0)	0
	Yuncheng, Yunfu	112.04E	22.93N	0/18 (0)	0
	Huidong, Huizhou	114.72E	22.99N	0/21 (0)	0
	Leizhou, Zhanjiang	110.10E	20.91N	0/26 (0)	0
	Haifeng, Shanwei	115.32E	22.97N	0/4 (0)	0
	Jiangmen	113.09E	22.59N	2/63 (3.17)	5–10
Guangxi (GX)	Yinhai, Beihai	109.14E	21.45N	0/47 (0)	0
	Cangwu, Wuzhou	111.54E	23.85N	12/49 (24.49)	1–24
	Luchuan, Yulin	110.16E	22.19N	25/70 (35.71)	2–18
	Nanning	108.21E	22.51N	12/87 (13.79)	1–11
	Guilin	110.28E	25.29N	22/33 (66.67)	3–15
	Lingui, Guilin	110.22E	25.22N	6/24 (25)	2–9
Hainan (HaN)	Bailian, Chengmai	110.13E	19.91N	0/13 (0)	0
	Haikou	110.37E	20.03N	2/21 (9.52)	1–2
	Wanning, Wuzhishan	110.40E	18.80N	9/37 (24.32)	2–18
Sichuan (SC)	Yingshan, Nanchong	106.57E	31.08N	5/16 (31.25)	1–8
	Nanchong	106.08E	30.78N	11/22 (50.00)	1–23
	Linshui, Guangan	106.93E	30.33N	8/27 (29.63)	2–31
	Luzhou	105.83E	28.82N	10/32 (31.25)	1–19
	Dazhou	107.45E	31.21N	2/12 (16.67)	4–5
	Dechang, Liangshanzhou	102.26E	27.88N	6/39 (15.38)	2–12
	Jiajiang, Leshan	103.73E	29.57N	6/23 (26.09)	2–5
	Rong, Zigong	104.81E	29.34N	3/45 (6.67)	1–4
Yunnan (YN)	Kunming	102.72E	25.05N	6/55 (10.91)	1–7
	Tengchong, Baoshan	98.50E	25.03N	4/26 (15.38)	3–5
	Lianghe, Dehongzhou	98.30E	24.82N	3/36 (8.33)	2–8
	Zhenkang, Lincang	98.83E	23.76N	0/10 (0)	0
	Tonghai, Yuxi	102.76E	24.11N	0/19 (0)	0
	Yanshan, Wenshan	104.34E	23.61N	2/40 (5)	1–3
	Yulong, Lijiang	100.24E	26.82N	0/23 (0)	0
	Mengzi, Honghe	103.36E	23.40N	1/26 (3.85)	1
Guizhou (GZ)	Zhengan, Zunyi	107.45E	28.55N	3/14 (21.43)	28–46
	Majiang, Kaili	107.63E	26.53N	9/37 (24.32)	1–8
	Xingren, Xingyi	104.93E	25.08N	0/3 (0)	0
	Changshun, Duyun	107.52E	26.27N	0/12 (0)	0
	Dejiang, Tongren	108.12E	28.26N	0/17 (0)	0
	Anshun	105.95E	26.25N	2/13 (15.38)	2–4
	Guiyang	106.63E	26.65N	2/9 (22.22)	2–3
Chongqing (CQ)	Nanbin, Shizhu	108.12E	30.00N	0/5 (0)	0
	Baijia, Liangping	107.80E	30.68N	5/50 (10)	1–3
	Baishi, Zhong	107.88E	30.31N	2/23 (8.7)	3
	Changsha, Kai	108.31E	30.40N	0/11 (0)	0
	Shaping, Dianjiang	107.44E	30.47N	0/14 (0)	0
	Shituo, Fuling	107.15E	29.71N	2/27 (7.41)	2–5
	Mawang, Youyang	108.96E	28.90N	2/12 (16.67)	1–5
	Yunyang	108.70E	30.93N	7/34 (20.59)	1–2
Qinghai (QH)	Huangzhong, Xining	101.48E	36.38N	0/7 (0)	0
Shaanxi (SaX)	Fengxiang, Baoji	107.40E	34.52N	0/19 (0)	0
	Qian, Xianyang	108.24E	34.53N	0/23 (0)	0
Ningxia (NX)	Yongning, Yinchuan	106.25E	38.28N	0/31 (0)	0

### Molecular identification

**Fig 2:**
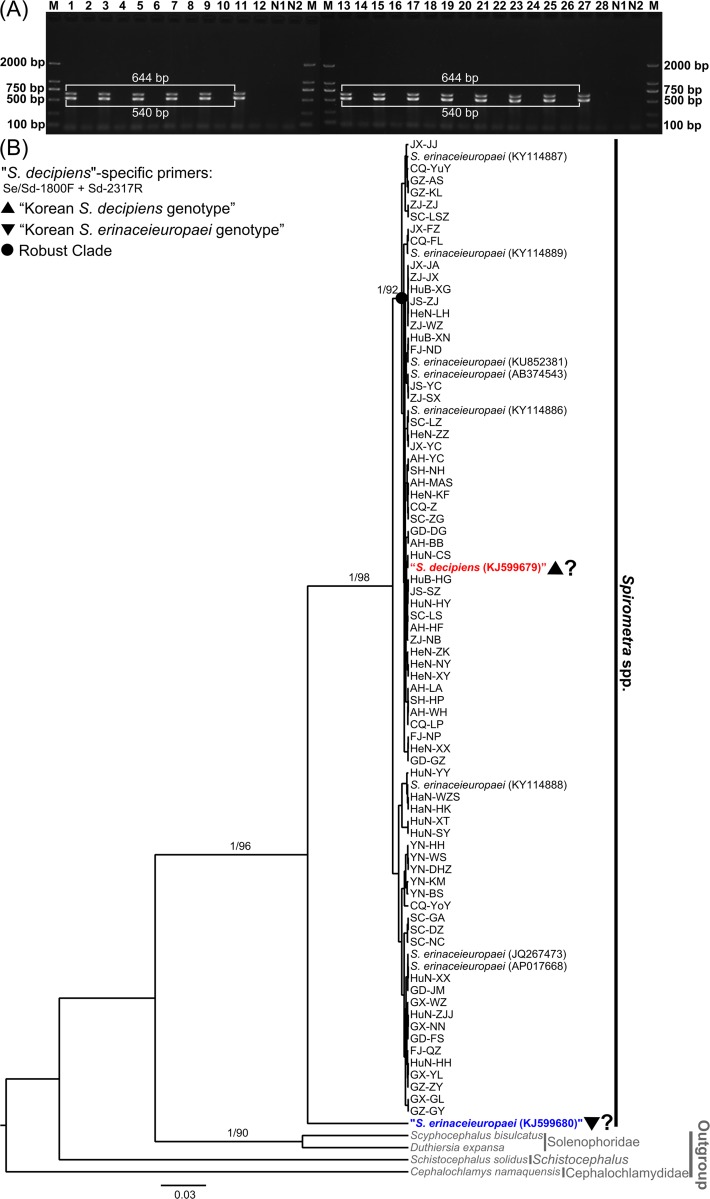
(A) An example of the multiplex PCR performed with species-specific primer sets. Lanes 1, 3, 5, 7, 9, 11, 13, 15, 17, 19, 21, 23, 25, and 27 indicate amplification with *S*. *decipiens*-specific primers (Se/Sd-1800F + Sd-2317R and Se/Sd-7955F + Sd-8567R). Lanes 2, 4, 6, 8, 10, 12, 14, 16, 18, 20, 22, 24, 26, and 28 indicate amplification with *S*. *erinaceieuropaei*-specific primers (Se/Sd-1800F + Se-2018R and Se/Sd-7955F + Se-8356R). Lanes 1–12, samples from Anhui Province; Lanes 13–22, samples from Yunnan Province; Lanes 23–28, samples from Fujian Province. M, DNA size marker (100 bp ladder). N1, negative control with *S*. *decipiens*-specific primers; N2, negative control with *S*. *erinaceieuropaei* -specific primers. (B) The phylogenetic analysis of the sequences of the PCR products amplified with the primers Se/Sd-1800F + Sd-2317R based on the maximum likelihood and Bayesian methods. The numbers along the branches indicate posterior probabilities and bootstrap values. Only posterior probabilities above 0.9 and bootstrap values above 90 are shown.

As shown in Fig 2A, all sparganum isolates collected from the 72 different geographical locations revealed two bands (540 bp and 644 bp) after amplification with the “Korean *S*. *decipiens* genotype” specific primers; however, when using the “Korean *S*. *erinaceieuropaei* genotype” specific primers, no bands were detected. For further identification, the PCR products were sequenced for comparison with the 10 referenced mitochondrial genomes of the *Spirometra* tapeworms from GenBank. The first band (using the primer pair of Se/Sd-1800F + Sd-2317R) was sequenced and consisted of 622 bp after trimming. The corresponding tree topologies generated by the two methods (ML and BI) were identical. As shown in Fig 2B, the earliest divergence gave rise to the Cephalochlamydidae (*C*. *namaquensis*), followed by *S*. *solidus*, and then to the 2 samples of Solenophoridae (*D*. *expansa* and *S*. *bisulcatus*). The last divergence gave rise to the remaining *Spirometra* isolates; the isolates collected in this study and the 10 *Spirometra* mitochondrial genomes from GenBank made up a single, highly supported group. Within *Spirometra*, two main clades were revealed. One clade included only a single isolate of the “Korean *S*. *erinaceieuropaei* genotype” (KJ599680). The Chinese isolates, “Korean *S*. *decipiens* genotype” (KJ599679) and 8 other *S*. *erinaceieuropaei* mitochondrial genomes were clustered in the other clade. Using the sequences of the second band (515 bp, using the primer pair of Se/Sd-7955F + Sd-8567R), the clustering pattern of the *Spirometra* samples was consistent with that generated based on the sequences of the first band (622 bp) (Supplementary S2 Fig). These phylogenetic patterns suggested that the taxonomic positions of the “Korean *S*. *decipiens* genotype” (KJ599679) and “Korean *S*. *erinaceieuropaei* genotype” (KJ599680) are controversial.

## Discussion

After the first human case of sparganosis was reported in Xiamen, Fujian Province in 1882, more than 1300 cases were reported from 1949 to 2014 in China [3]. Eating raw or undercooked frog/snake meat, using raw frog/snake flesh in traditional poultices and ingesting live frog tadpoles are risk factors for infection [4]. Therefore, in addition to snakes, frogs also play an important role in the spread of human sparganosis in China. From 2013–2018, we conducted a large-scale survey of sparganum infection in wild frogs from 145 geographical locations to understand the prevalence of spirometrid tapeworms in wild frogs.

In previous investigations of sparganum infections in wild frogs in China, the frog species that were reported to be sensitive to infection included *P*. *nigromaculatus*, *F*. *limnocharis*, *H*. *chinensis*, *R*. *chensinensis*, *R*. *rugulosa* and *Bufo gargarizans* [6, 12, 29, 30]. In our survey, sparganum infection was also found in *P*. *nigromaculatus*, *F*. *limnocharis* and *H*. *chinensis*. In addition, 5 other frog species: *P*. *plancyi*, *S*. *latouchii*, *B*. *guentheri*, *Q*. *spinosa* and *O*. *margaretae*, have been proven to be sensitive to sparganum infections. Among these frog species, the most frequently infected species was *P*. *nigromaculatus*, which indicates that the species is important in the prevention and control of human sparganosis in China. Although many specimens of *R*. *chensinensis* have been collected here, no sparganum-positive frogs were found. In addition, specimens of *R*. *rugulosa* and *B*. *gargarizans* were not collected in this survey.

Many human cases of sparganosis have been reported in Hunan province; accordingly, the sparganum infection in wild frogs in Hunan was the highest in our survey, but it was still slightly lower than that in a previous survey in this region [6]. High prevalence of human sparganosis has also been reported in Guangdong and Zhejiang [3]; however, the infection rates in these regions were moderate. For example, in the Guangdong province, which accounts for 10% of the reported human sparganosis cases in China [7], the infection rate was 7.73% in wild frogs. However, a previous survey of the province suggested that 35% of wild frogs had sparganum infections [8]. In addition, frog meat, as a type of “bushmeat”, has an important role in the cuisine of Guangdong, and approximately 59.9% of the residents eat frog meat [8, 11]. Thus, the risk of infection in Guangdong remains high. In Zhejiang province, the infection rate detected in this study was 10.86%; in contrast, a previous report found that the infection rate in Hangzhou, Zhejiang, reached 31.15% [31]. Compared with the other regions, we chose the most sampling sites and examined the highest number of frogs in Henan province in central China. Therefore, the data generated in this region were the most representative. The infection rate in Henan was lower than those in most of the southern and southwestern regions; however, the number of reported human cases was higher than those from the other regions. Before 2006, human sparganosis in Henan was rarely reported (only 3 imported cases from southern China). After 2006, twenty autochthonous cases caused by the ingestion of live tadpoles emerged because some rural villagers in this region believe that live tadpole consumption has a medicinal role in skin diseases [4, 32], which suggests that several traditional Chinese folk remedies are problematic for the prevention of human sparganosis. This incident was not the only case of human sparganosis that was caused by folk remedies: many cases of human sparganosis in Fujian in Southeastern China were caused by using fresh frog flesh as a poultice for sore eyes [33]. Generally, the infection rates in most of the south and southwest regions (Guangxi, Sichuan, Hainan, Guizhou and Chongqing) were higher than those in the other regions. These regions contain many minorities who have different dietary customs, and frog meat is a delicacy for many people [13]. Although few human cases have been reported, the high sparganum infection rate suggests that people living in these regions are at an increased risk of infection. One surprising finding was that no infected frogs were found in Northeast China (Heilongjiang, Jilin and Liaoning) over the 6 year study period, although many cases have been reported in these regions [34–36]. The possible reasons were that (1) the number of sampling sites and the sample sizes were small, especially in Liaoning and Jilin, as only 2 and 3 sampling sites were selected, respectively; (2) the main frog species collected in the northeastern regions was *R*. *chensinensis*, but no infection was found in *R*. *chensinensis* frogs in this study; or (3) the reported human cases in Northeast China were probably caused by the importation of infected frogs/snakes from other regions of China. Human sparganosis cases have also been reported in Beijing and Hebei in North China and Qinghai in Northwest China, but no sparganum-positive frogs were detected in our survey. It should come as no surprise that no infected frogs were found in the non-endemic regions of Shaanxi, Shanxi, Ningxia and Inner Mongolia in North and Northwest China. In summary, this survey indicated the following: (1) sparganum infection in wild frogs was detected in 16 of the 23 surveyed regions where human sparganosis is endemic in China; (2) eating wild frogs is associated with considerable health risks in China, especially in the southern and southwestern regions, and improper cooking methods may increase the risk of infection; and (3) several traditional Chinese folk remedies play an important role in the spread of human sparganosis; therefore, health education should be strengthened to prevent the transmission of this disease.

The accurate morphological identification of *Spirometra* tapeworm species must be based on the morphology of the adult worms; however, the specimens obtained in the field are usually larval forms. It is impossible to distinguish plerocercoids using only morphological characteristics. Therefore, an increasing number of researchers have utilized molecular methods to identify spirometrid tapeworms [19, 20, 37]. Recently, Jeon and colleagues developed a multiplex PCR assay to specifically distinguish the Korean “*S*. *erinaceieuropaei*” and “*S*. *decipiens*” genotypes [18]. Using the multiplex PCR system, isolates from 72 different locations revealed uniform electrophoretic bands. Furthermore, the clustering analysis of the sequenced PCR bands suggested that the taxonomic positions of the “Korean *S*. *decipiens* genotype” (KJ599679) and “Korean *S*. *erinaceieuropaei* genotype” (KJ599680) are controversial. In agreement with our results, using the *cox*1 gene, Almeida et al [19] suggested that “*S*. *decipiens*” (KJ599679) is likely conspecific with the Asian isolates of *S*. *erinaceieuropaei* and that “*S*. *erinaceieuropaei*” (KJ599680) is a different species of *Spirometra*. Therefore, the controversial results here probably indicate the following: (1) if “*S*. *decipiens*” (KJ599679) was correctly identified, all of the Chinese isolates and the 8 mitochondrial genomes in GenBank should be *S*. *decipiens*; otherwise, “*S*. *decipiens*” (KJ599679) should be a conspecific of *S*. *erinaceieuropaei*; and (2) “*S*. *erinaceieuropaei*” (KJ599680) might be a different species of *Spirometra* or a special genotype that differed from those of other isolates. Nevertheless, as described above, the accurate identification of a species must be based on its morphological characteristics, so new studies especially comprehensive morphological analyses, are needed to accurately identify *Spirometra* species.

Traditionally, the plerocercoids in the Far East were called Manson’s tapeworm. The scientific name of Manson’s tapeworm has been described in many text books as *Spirometra mansoni*, but as more detailed studies have been conducted, parasitologists have divided it into many “species” according to its morphological characteristics [16]. However, Iwata [16] proposed that only *S*. *erinacei* exists and that other species have been described only because the characteristics used for identification vary with the environmental conditions of the host and the developmental stage and especially the position of the proglottid in the strobila. *S*. *erinacei* was first reported by Rudoiphi in 1819 as a sparganum isolated from a European hedgehog (*Erinaceus europaeus*). Then, the name was changed to *S*. *erinaceieuropaei* in 1959 [38]. Currently, this species has been reported as the major aetiological agent of human sparganosis worldwide [3, 5, 39]. On the other hand, Mueller [40] concluded that *S*. *mansonoides* is also a valid species based on a uterine trait. Later, the validation of *S*. *mansonoides* was supported by the molecular data [41]. Recently, Jeon et al. [24] reported that the “species” *S*. *decipiens* (Gedoelst, 1911) in Korea is infectious to humans. They noted that the main characteristic for “*S*. *erinaceieuropaei*” and “*S*. *decipiens*” differentiation is the spiral coiled uterus; “*S*. *erinaceieuropaei*” has 5–7 complete coils, while “*S*. *decipiens*” has 4–4.5 coils. According to Iwata [16], the uterus generally has 3–6 coils but can have 7–8 coils in long, young proglottids or only 2 coils (rarely one) in the anterior proglottids of young worms or the posterior proglottids of overmature worms; thus, the number of uterine coils cannot be regarded as a specific characteristic. In addition, the forms of the uterus, testes, vitellaria, uterine pore, genital pouch, etc. are changed by the conditions of contraction and cannot be regarded as specific characteristics either. In the most recent reviews [15, 42], only 4 valid species of the genus *Spirometra* have been accepted: *S*. *erinaceieuropaei* (Rudolphi, 1819) Faust, Campbell & Kellogg, 1929; *S*. *mansonoides* (Mueller, 1935) Mueller, 1936; *S*. *pretoriensis* (Baer, 1924) Wardle, McLeod & Stewart, 1947; and *S*. *theileri* (Baer, 1924) Opuni & Muller, 1974. Accordingly, “*S*. *decipiens*” should be considered a conspecific of *S*. *erinaceieuropaei*. Therefore, the systematics of *Spirometra* spp. are still complex, and more morphological and molecular phylogenetic analyses are necessary to clarify the taxonomy of the genus in the future.

## Conclusions

In this survey, 8 frog species were found to be sensitive to sparganum infection, and the most frequently infected species was *P*. *nigromaculatus*. The sparganum infection rates in wild frogs in several regions of China were still high, especially in South and Southwest China. Eating wild frogs especially with improper cooking methods, is associated with considerable health risks in China. Several traditional Chinese folk remedies may increase the risk of infection. The molecular identification suggested that the taxonomic positions of “*S*. *decipiens*” (KJ599679) and “*S*. *erinaceieuropaei*” (KJ599680) are controversial. The sparganum isolates collected here were more likely the species of *S*. *erinaceieuropaei*, but new studies, especially comprehensive morphological analyses, are needed in the future.

## Supporting information

S1 Table The information of the origin, locality, collectors and date of collection for each sampling site. (DOC)

S2 Table The sparganum isolates used for multiplex PCR diagnosis and sequencing analysis. (DOC)

S3 Table The multiplex PCR primers used in the present study. (DOC)

S4 Table The prevalence of *Spirometra* sparganum infection in different locations of China and the parasitizing sites of spargana in frogs. (DOC)

S1 Fig The collection locations and the presence of sparganum parasitization in frogs. (DOC)

S2 Fig The maximum likelihood and Bayesian tree based on the sequences of the PCR products amplified with the primers Se/Sd-7955F + Sd-8567R. (DOC)

S1 Dataset The sequencing data of the sparganum isolates used in this study. (DOC)
