# Supplementary material for: Large-scale survey of a neglected agent of sparganosis Spirometra erinaceieuropaei (Cestoda: Diphyllobothriidae) in wild frogs in China
Source: PLoS Negl Trop Dis. 2020 Feb 26;14(2):e0008019. doi: 10.1371/journal.pntd.0008019 (PMC7043720; doi:10.1371/journal.pntd.0008019)
Supplement: S1 Table — (DOC) [file pntd.0008019.s001.doc]

**S1 Table.** The information of the origin, locality, collectors and date of collection for each sampling site.

| Origin | | Longitude | Latitude | Collection date | Collector |
| --- | --- | --- | --- | --- | --- |
| Province/autonomous region/municipality | County |  |  |  |  |
| Inner Mongolia (IM) | Wulanhaote, Xinganmeng | 122.23E | 46.03N | 30 Aug, 2017 | Fu J |
| Hebei (HeB) | Qiaodong, Xingtai | 114.51E | 37.07N | 4 Jul, 2014 | Li X |
|  | Shijiazhuang | 114.26E | 38.03N | 19 Jun, 2016 | Li L |
| Beijing (BJ) | Yanqing | 116.14E | 40.31N | 2 Jul, 2015 | Zhang Y |
| Tianjin (TJ) | Dongli | 117.31E | 39.09N | 31 Aug, 2014 | Chen YY |
| Shanxi (SX) | Jincheng | 112.83E | 35.52N | 18 Jun, 2014 | Deng JW |
| Heilongjiang (HLJ) | Qitaihe | 130.49E | 45.48N | 9 Sept, 2014 | Song YX |
|  | Qinggang, Suihua | 126.59E | 46.38N | 16 Jul, 2017 | Ren T |
|  | Daqing | 125.01E | 46.36N | 31 Jul, 2017 | Ren T |
|  | Acheng , Harbin | 126.96E | 45.55N | 21 Aug, 2017 | Teng S |
|  | Baoquanling, Hegang | 130.53E | 47.43N | 25 Jul, 2017 | Wang QB |
|  | Daxinganling | 125.47E | 50.10N | 13 Aug, 2017 | Qiao LY |
| Jilin (JL) | Changchun | 125.32E | 43.82N | 16 Jul, 2014 | Zhou XZ |
|  | Baicheng | 122.84E | 45.62N | 20 Jul, 2017 | Xu XT |
|  | Siping | 124.37E | 43.17N | 13 Aug, 2017 | Jiang M |
| Liaoning (LN) | Kaiyuan, Tieling | 124.04E | 42.55N | 26 Jul, 2017 | Li JH |
|  | Tieling | 123.84E | 42.29N | 8 Aug, 2017 | Shi FH |
| Shandong (SD) | Yantai | 121.39E | 37.50N | 15 Jul, 2017 | Lin FX |
|  | Juancheng, Heze | 115.51E | 35.56N | 28 Jul, 2014 | Sun L |
|  | Rushan, Weihai | 121.54E | 36.92N | 12 Aug, 2017 | Lin JB |
|  | Gaomi, Weifang | 119.76E | 36.38N | 22 Jul, 2017 | Chi YJ |
|  | Pingdu, Qingdao | 119.99E | 36.78N | 25 Jul, 2017 | Chi YJ |
| Anhui (AH) | Yizhou, Yicheng | 118.75E | 30.95N | 14 Jul, 2014 | Yu Q |
|  | Lujiang, Hefei | 117.25E | 31.88N | 11 Jul, 2014 | Jiang DK |
|  | Wuwei, Wuhu | 118.57E | 31.15N | 16 Jul, 2014 | Peng J |
|  | Linquan, Fuyang | 115.26E | 33.04N | 15 Jul, 2015 | Rao YF |
|  | Huoqiu, Luan | 116.28E | 32.35N | 17 Jul, 2016 | Zeng DJ |
|  | Yuexi, Anqing | 116.36E | 30.85N | 24 Jul, 2017 | Liu J |
|  | Dangtu, Maanshan | 118.50E | 31.57N | 22 Jul, 2017 | Tao M |
|  | Qiaocheng, Bozhou | 115.78E | 33.88N | 21 Jul, 2017 | Li GY |
|  | Bengbu | 117.36E | 32.94N | 16 Jul, 2014 | He RZ |
| Jiangsu (JS) | Kunshan, Suzhou | 120.98E | 31.38N | 8 Jul, 2014 | Tian X |
|  | Pizhou, Xuzhou | 118.01E | 34.34N | 15 Jul, 2014 | Gao YK |
|  | Runzhou, Zhenjiang | 119.41E | 32.20N | 7 Jul, 2014 | Zhou J |
|  | Funing, Yancheng | 119.80E | 33.78N | 14 Jul, 2014 | Zhu XY |
|  | Dafeng, Yancheng | 120.50E | 33.20N | 1 Sept, 2018 | Chen ZY |
|  | Ganyu, Lianyungang | 119.17E | 34.84N | 16 Jul, 2014 | Yang Q |
| Zhejiang (ZJ) | Pinghu, Jiaxing | 121.02E | 30.70N | 12 Jul, 2014 | Fang B |
|  | Yuyao, Ningbo | 121.15E | 30.03N | 13 Jul, 2014 | He RZ |
|  | Beilun, Ningbo | 121.85E | 29.93N | 9 Aug, 2014 | Wang SH |
|  | Cixi, Ningbo | 121.23E | 30.17N | 4 Aug, 2014 | Jin YB |
|  | Shaoxing, Shaoxing | 120.47E | 30.08N | 21 Jul, 2014 | Bao HJ |
|  | Wucheng, Jinhua | 119.57E | 29.09N | 26 Jun, 2015 | Hu CX |
|  | Dinghai, Zhoushan | 122.11E | 30.02N | 17 Jul, 2015 | Wang Y |
|  | Ouhai, Wenzhou | 120.61E | 27.97N | 26 Jul, 2016 | Guo QQ |
|  | Dongbaihu, Zhuji | 120.38E | 29.58N | 20 Jul, 2016 | Wu F |
| Jiangxi (JX) | Linchuan, Fuzhou | 116.31E | 27.93N | 8 Jul, 2014 | Xu JF |
|  | Chonggang, Fuzhou | 116.38E | 27.90N | 15 Jul, 2014 | Chen X |
|  | Chongren, Fuzhou | 116.06E | 27.77N | 15 Aug, 2014 | Liao TT |
|  | Xingzi, Jiujiang | 116.05E | 29.45N | 15 Jul, 2014 | Lei XH |
|  | Jishui, Jian | 115.14E | 27.23N | 4 Aug, 2016 | Peng M |
|  | Yushui, Xinyu | 115.14E | 27.23N | 23 Aug, 2016 | Du CH |
|  | Yifeng, Yichun | 114.80E | 28.39N | 26 Aug, 2016 | Du CH |
|  | Xinjian, Nanchang | 115.82E | 28.69N | 24 Aug, 2017 | Xiong C |
| Fujian (FJ) | Xianyou, Putian | 118.69E | 25.36N | 25 Jul, 2015 | Yu L |
|  | Tongan, Xiamen | 118.15E | 24.72N | 8 Aug, 2015 | Zheng LH |
|  | Shouning, Ningde | 119.51E | 27.45N | 21 Jul, 2016 | Miao LJ |
|  | Pingtan, Fuzhou | 119.79E | 25.50N | 26 Jul, 2015 | Lin AM |
|  | Quanzhou | 118.68E | 24.87N | 10 Jul, 2017 | Lin YH |
|  | Yanghou, Nanping | 118.52E | 26.63N | 24 Jul, 2016 | Rao JH |
|  | Haikou, Fuqing | 119.47E | 25.70N | 26 Jul, 2017 | Lu YH |
| Shanghai (SH) | Nanhui | 121.85E | 30.86N | 21 Jul, 2016 | Wang SZ |
|  | Huangpu | 121.48E | 31.23N | 18 Aug, 2016 | Wang SZ |
|  | Songjiang | 121.45E | 31.03N | 18 Aug, 2017 | Liu J |
| Henan (HeN) | Zhengzhou | 113.65E | 34.73N | 30 Aug, 2013 | Liu LN |
|  | Nanle, Puyang | 115.20E | 36.07N | 9 Aug, 2018 | Chen HY |
|  | Nanzhao, Nanyang | 112.43E | 33.49N | 7 Aug, 2018 | Liu X |
|  | Lushan, Pingdingshan | 112.91E | 33.74N | 31 Aug, 2018 | Bai H |
|  | Shihe, Xinyang | 114.06E | 32.10N | 12 Aug, 2018 | Li N |
|  | Huangchuan, Xinyang | 115.05E | 32.13N | 14 Jul, 2018 | Zhang YS |
|  | Yongcheng | 116.45E | 33.93N | 19 Jul, 2018 | Fang L |
|  | Xiayi, Shangqiu | 116.13E | 34.24N | 22 Jul, 2018 | Gong C |
|  | Hua, Anyang | 114.52E | 35.58N | 9 Jul, 2018 | Li F |
|  | Xinxiang | 113.87E | 35.30N | 7 Aug, 2013 | Wen H |
|  | Kaifeng | 114.47E | 34.48N | 19 Jul, 2013 | Zhang ZW |
|  | Fugou, Zhoukou | 114.38E | 34.07N | 17 Aug, 2013 | Wang ZQ |
|  | Luohe | 114.02E | 33.58N | 24 Jul, 2013 | Weitong |
| Hubei (HuB) | Yunmeng, Xiaogan | 113.75E | 31.02N | 16 Jul, 2015 | Zhang B |
|  | Xiangzhou, Xiangyang | 112.21E | 32.09N | 21 Jul, 2015 | Liu YL |
|  | Chongyang, Xianning | 114.04E | 29.56N | 15 Jul, 2015 | Wang JL |
|  | Yunxi, Shiyan | 110.43E | 32.99N | 17 Jul, 2015 | Ke XT |
|  | Huanggang | 114.88E | 30.45N | 28 Jul, 2016 | Gao M |
|  | Laifeng, Enshi | 109.41E | 29.49N | 8 Sept, 2018 | Zhang R |
|  | Mingshan, Daye | 114.76E | 30.07N | 2 Sept, 2018 | Wang BT |
| Hunan (HuN) | Sangzhi, Zhangjiajie | 110.20E | 29.41N | 19 Jul, 2015 | Zhong K |
|  | Xupu, Huaihua | 110.59E | 27.91N | 30 Jul, 2015 | Zhong K |
|  | Shaodong, Shaoyang | 111.74E | 27.26N | 9 Aug, 2015 | Wang CY |
|  | Huarong, Yueyang | 112.54E | 29.53N | 28 Jul, 2015 | Jiang F |
|  | Leiyang, Hengyang | 112.83E | 26.31N | 3 Aug, 2016 | Li HQ |
|  | Yunhuqiao, Xiangtan | 112.73E | 27.85N | 18 Jul, 2015 | Wang M |
|  | Fenghuang, Xiangxi | 109.58E | 27.96N | 8 Aug, 2016 | Wu QW |
|  | Changsha | 113.04E | 28.14N | 26 Jun, 2013 | Cui J |
| Guangdong (GD) | Chashan, Dongguan | 113.87E | 23.08N | 27 Jul, 2014 | Peng XE |
|  | Shunde, Fushan | 113.29E | 22.81N | 17 Aug, 2014 | Wang QM |
|  | Guangzhou | 113.26E | 23.13N | 18 Jul, 2015 | Zhang YS |
|  | Baoan, Shenzhen | 113.88E | 22.56N | 10 Aug, 2016 | Du CH |
|  | Yuncheng, Yunfu | 112.04E | 22.93N | 23 Jul, 2016 | Yu SM |
|  | Huidong, Huizhou | 114.72E | 22.99N | 16 Jul, 2017 | Luo WJ |
|  | Leizhou, Zhanjiang | 110.10E | 20.91N | 14 Jul, 2017 | Huang SJ |
|  | Haifeng, Shanwei | 115.32E | 22.97N | 28 Jul, 2017 | Luo WT |
|  | Jiangmen | 113.09E | 22.59N | 22 Jul, 2017 | Yi SQ |
| Guangxi (GX) | Yinhai, Beihai | 109.14E | 21.45N | 24 Jul, 2015 | Yin XQ |
|  | Cangwu, Wuzhou | 111.54E | 23.85N | 18 Jul, 2016 | Wang SS |
|  | Luchuan, Yulin | 110.16E | 22.19N | 16 Jul, 2013 | Lu JC |
|  | Nanning | 108.21E | 22.51N | 9 Aug, 2013 | Jiang J |
|  | Guilin | 110.28E | 25.29N | 30 Aug, 2013 | Li LY |
|  | Lingui, Guilin | 110.22E | 25.22N | 16 Aug, 2013 | Li LY |
| Hainan (HaN) | Bailian, Chengmai | 110.13E | 19.91N | 1 Aug, 2014 | Zhang YB |
|  | Haikou | 110.37E | 20.03N | 30 Jul, 2013 | Wu DF |
|  | Wanning, Wuzhishan | 110.40E | 18.80N | 17 Jul, 2013 | Wang C |
| Sichuan (SC) | Yingshan, Nanchong | 106.57E | 31.08N | 15 Jul, 2014 | Xiang Y |
|  | Nanchong | 106.08 E | 30.78 N | 5 Jul, 2014 | Man YX |
|  | Linshui, Guangan | 106.93E | 30.33N | 26 Jul, 2014 | Huang KP |
|  | Luzhou | 105.83E | 28.82N | 13 Jul, 2014 | Huang XQ |
|  | Dazhou | 107.45E | 31.21N | 18 Jul, 2015 | Fu GM |
|  | Dechang, Liangshanzhou | 102.26E | 27.88N | 12 Jul, 2015 | Xu MG |
|  | Jiajiang, Leshan | 103.73E | 29.57N | 15 Jul, 2015 | Wang LF |
|  | Rong, Zigong | 104.81E | 29.34N | 31 Jul, 2015 | Liu Y |
| Yunnan (YN) | Kunming | 102.72E | 25.05N | 23 Jul, 2014 | Wang LA |
|  | Tengchong, Baoshan | 98.50E | 25.03N | 25 Jul, 2014 | Yang LD |
|  | Lianghe, Dehongzhou | 98.30E | 24.82N | 12 Aug, 2014 | Dong XX |
|  | Zhenkang, Lincang | 98.83E | 23.76N | 7 Aug, 2015 | Fu GM |
|  | Tonghai, Yuxi | 102.76E | 24.11N | 2 Sept, 2018 | Song BL |
|  | Yanshan, Wenshan | 104.34E | 23.61N | 5 Aug, 2016 | Chen Y |
|  | Yulong, Lijiang | 100.24E | 26.82N | 18 Aug, 2016 | He CX |
|  | Mengzi, Honghe | 103.36E | 23.40N | 17 Jul, 2016 | Xiang X |
| Guizhou (GZ) | Zhengan, Zunyi | 107.45E | 28.55N | 30 Jul, 2014 | Yu Z |
|  | Majiang, Kaili | 107.63E | 26.53N | 11 Jul, 2014 | Wu XQ |
|  | Xingren, Xingyi | 104.93E | 25.08N | 19 Aug, 2014 | Xie DD |
|  | Changshun, Duyun | 107.52E | 26.27N | 30 Jul, 2014 | Luo P |
|  | Dejiang, Tongren | 108.12E | 28.26N | 2 Aug, 2018 | Feng WJ |
|  | Anshun | 105.95E | 26.25N | 7 Aug, 2013 | Cui J |
|  | Guiyang | 106.63E | 26.65N | 2 Aug, 2013 | Cui J |
| Chongqing (CQ) | Nanbin, Shizhu | 108.12E | 30.00N | 20 Jul, 2014 | Huang KP |
|  | Baijia, Liangping | 107.80E | 30.68N | 16 Jul, 2014 | Liu J |
|  | Baishi, Zhong | 107.88E | 30.31N | 21 Jul, 2015 | Chen Y |
|  | Changsha, Kai | 108.31E | 30.40N | 28 Aug, 2015 | Liu L |
|  | Shaping , Dianjiang | 107.44E | 30.47N | 13 Aug, 2017 | Lu WT |
|  | Shituo, Fuling | 107.15E | 29.71N | 17 Aug, 2018 | Wang J |
|  | Mawang, Youyang | 108.96E | 28.90N | 21 Aug, 2018 | Mao Z |
|  | Yunyang | 108.70E | 30.93N | 2 Aug, 2018 | Ye M |
| Qinghai (QH) | Huangzhong, Xining | 101.48E | 36.38N | 14 Aug, 2016 | Dou HM |
| Shaanxi (SaX) | Fengxiang, Baoji | 107.40E | 34.52N | 14 Aug, 2014 | Liu S |
|  | Qian, Xianyang | 108.24E | 34.53N | 15 Jul, 2014 | Liu S |
| Ningxia (NX) | Yongning, Yinchuan | 106.25E | 38.28N | 17 Jul, 2014 | Wang J |
