# Supplementary material for: Large-scale survey of a neglected agent of sparganosis Spirometra erinaceieuropaei (Cestoda: Diphyllobothriidae) in wild frogs in China
Source: PLoS Negl Trop Dis. 2020 Feb 26;14(2):e0008019. doi: 10.1371/journal.pntd.0008019 (PMC7043720; doi:10.1371/journal.pntd.0008019)
Supplement: S2 Table — (DOC) [file pntd.0008019.s002.doc]

**S2 Table.** The sparganum isolates used for multiplex PCR diagnosis and sequencing analysis.

| Sample code | Origin |  | Locality | |
| --- | --- | --- | --- | --- |
|  | Province/Autonomous region/Municipality | City | Longitude | Latitude |
| AH-YC | Anhui | Yizhou, Yicheng | 118.75E | 30.95N |
| AH-HF |  | Lujiang, Hefei | 117.25E | 31.88N |
| AH-WH |  | Wuwei, Wuhu | 118.57E | 31.15N |
| AH-LA |  | Huoqiu, Luan | 116.28E | 32.35N |
| AH-MAS |  | Dangtu, Maanshan | 118.50E | 31.57N |
| AH-BB |  | Bengbu | 117.36E | 32.94N |
| JS-SZ | Jiangsu | Kunshan, Suzhou | 120.98E | 31.38N |
| JS-ZJ |  | Runzhou, Zhenjiang | 119.41E | 32.20N |
| JS-YC |  | Funing, Yancheng | 119.80E | 33.78N |
| ZJ-JX | Zhejiang | Pinghu, Jiaxing | 121.02E | 30.70N |
| ZJ-NB |  | Cixi, Ningbo | 121.23E | 30.17N |
| ZJ-SX |  | Shaoxing | 120.47E | 30.08N |
| ZJ-WZ |  | Ouhai, Wenzhou | 120.61E | 27.97N |
| ZJ-ZJ |  | Dongbaihu, Zhuji | 120.38E | 29.58N |
| JX-FZ | Jiangxi | Linchuan, Fuzhou | 116.31E | 27.93N |
| JX-JJ |  | Xingzi, Jiujiang | 116.05E | 29.45N |
| JX-JA |  | Jishui, Jian | 115.14E | 27.23N |
| JX-YC |  | Yifeng, Yichun | 114.80E | 28.39N |
| FJ-ND | Fujian | Shouning, Ningde | 119.51E | 27.45N |
| FJ-QZ |  | Quanzhou | 118.68E | 24.87N |
| FJ-NP |  | Yanghou, Nanping | 118.52E | 26.63N |
| SH-NH | Shanghai | Nanhui | 121.85E | 30.86N |
| SH-HP |  | Huangpu | 121.48E | 31.23N |
| HeN-ZZ | Henan | Zhengzhou | 113.65E | 34.73N |
| HeN-NY |  | Nanzhao, Nanyang | 112.43E | 33.49N |
| HeN-XY |  | Shihe, Xinyang | 114.06E | 32.10N |
| HeN-XX |  | Xinxiang | 113.87E | 35.30N |
| HeN-KF |  | Kaifeng | 114.47E | 34.48N |
| HeN-ZK |  | Fugou, Zhoukou | 114.38E | 34.07N |
| HeN-LH |  | Luohe | 114.02E | 33.58N |
| HuB-XG | Hubei | Yunmeng, Xiaogan | 113.75E | 31.02N |
| HuB-XN |  | Chongyang, Xianning | 114.04E | 29.56N |
| HuB-HG |  | Huanggang | 114.88E | 30.45N |
| HuN-ZJJ | Hunan | Sangzhi, Zhangjiajie | 110.20E | 29.41N |
| HuN-HH |  | Xupu, Huaihua | 110.59E | 27.91N |
| HuN-SY |  | Shaodong, Shaoyang | 111.74E | 27.26N |
| HuN-YY |  | Huarong, Yueyang | 112.54E | 29.53N |
| HuN-HY |  | Leiyang, Hengyang | 112.83E | 26.31N |
| HuN-XT |  | Yunhuqiao, Xiangtan | 112.73E | 27.85N |
| HuN-XX |  | Fenghuang, Xiangxi | 109.58E | 27.96N |
| HuN-CS |  | Changsha | 113.04E | 28.14N |
| GD-DG | Guangdong | Chashan, Dongguan | 113.87E | 23.08N |
| GD-GZ |  | Guangzhou | 113.26E | 23.13N |
| GD-JM |  | Jiangmen | 113.09E | 22.59N |
| GD-FS |  | Shunde, Fushan | 113.29E | 22.81N |
| GX-WZ | Guangxi | Cangwu, Wuzhou | 111.54E | 23.85N |
| GX-YL |  | Luchuan, Yulin | 110.16E | 22.19N |
| GX-NN |  | Nanning | 108.21E | 22.51N |
| GX-GL |  | Guilin | 110.28E | 25.29N |
| HaN-HK | Hainan | Haikou | 110.37E | 20.03N |
| HaN-WZS |  | Wanning, Wuzhishan | 110.40E | 18.80N |
| SC-NC | Sichuan | Nanchong | 106.08E | 30.78N |
| SC-GA |  | Linshui, Guangan | 106.93E | 30.33N |
| SC-LZ |  | Luzhou | 105.83E | 28.82N |
| SC-DZ |  | Dazhou | 107.45E | 31.21N |
| SC-LSZ |  | Dechang, Liangshanzhou | 102.26E | 27.88N |
| SC-LS |  | Jiajiang, Leshan | 103.73E | 29.57N |
| SC-ZG |  | Rong, Zigong | 104.81E | 29.34N |
| YN-KM | Yunnan | Kunming | 102.72E | 25.05N |
| YN-BS |  | Tengchong, Baoshan | 98.50E | 25.03N |
| YN-DHZ |  | Lianghe, Dehongzhou | 98.30E | 24.82N |
| YN-WS |  | Yanshan, Wenshan | 104.34E | 23.61N |
| YN-HH |  | Mengzi, Honghe | 103.36E | 23.40N |
| GZ-ZY | Guizhou | Zhengan, Zunyi | 107.45E | 28.55N |
| GZ-KL |  | Majiang, Kaili | 107.63E | 26.53N |
| GZ-AS |  | Anshun | 105.95E | 26.25N |
| GZ-GY |  | Guiyang | 106.63E | 26.65N |
| CQ-LP | Chongqing | Baijia, Liangping | 107.80E | 30.68N |
| CQ-Z |  | Baishi, Zhong | 107.88E | 30.31N |
| CQ-FL |  | Shituo, Fuling | 107.15E | 29.71N |
| CQ-YoY |  | Mawang, Youyang | 108.96E | 28.90N |
| CQ-YuY |  | Yunyang | 108.70E | 30.93N |
