# Supplementary material for: Large-scale survey of a neglected agent of sparganosis Spirometra erinaceieuropaei (Cestoda: Diphyllobothriidae) in wild frogs in China
Source: PLoS Negl Trop Dis. 2020 Feb 26;14(2):e0008019. doi: 10.1371/journal.pntd.0008019 (PMC7043720; doi:10.1371/journal.pntd.0008019)
Supplement: S3 Table — (DOC) [file pntd.0008019.s003.doc]

**S3 Table.** The multiplex PCR primers used in the present study.

| Primer | Primer sequence (5’-3’) | Reference |
| --- | --- | --- |
| Se/Sd-1800F | TAT TTT CGG TTG GTG CTG TAG | Jeon et al. (2018) |
| Se-2018R | CCA CAA ACC CAA TAA CAA ACT A |
| Sd-2317R | TCC TCC CCC CAC ACG ACA AAA |
| Se/Sd-7955F | ACG TGG TTT GTG GTG GCT CAT TTT |
| Se-8356R | ATG ATA GGG TAT AGG TGA CCA |
| Sd-8567R | TTA TTA ACT TCC TAA CCA ACT TGA TAC |

Jeon HK, Kim KH, Sohn WM, Eom KS (2018) Differential diagnosis of human sparganosis using multiplex PCR. Korean J Parasitol 56: 295-300.
