# Supplementary material for: Large-scale survey of a neglected agent of sparganosis Spirometra erinaceieuropaei (Cestoda: Diphyllobothriidae) in wild frogs in China
Source: PLoS Negl Trop Dis. 2020 Feb 26;14(2):e0008019. doi: 10.1371/journal.pntd.0008019 (PMC7043720; doi:10.1371/journal.pntd.0008019)
Supplement: S4 Table — (DOC) [file pntd.0008019.s004.doc]

**S4 Table.** The prevalence of *Spirometra* sparganum infection in different locations of China and the parasitizing sites of spargana in frogs.

| Province/autonomous region/municipality | Number examined | Number positive | Infection rate (%) | Total number of spargana | Number of the identified spargana and parasitic sites | | | | |
| --- | --- | --- | --- | --- | --- | --- | --- | --- | --- |
|  |  |  |  |  | thigh | forelimb | backside | abdomen | other |
| Inner Mongolia (IM) | 20 | 0 | 0 | 0 | 0 | 0 | 0 | 0 | 0 |
| Hebei (HeB) | 18 | 0 | 0 | 0 | 0 | 0 | 0 | 0 | 0 |
| Beijing (BJ) | 13 | 0 | 0 | 0 | 0 | 0 | 0 | 0 | 0 |
| Tianjin (TJ) | 16 | 0 | 0 | 0 | 0 | 0 | 0 | 0 | 0 |
| Shanxi (SX) | 25 | 0 | 0 | 0 | 0 | 0 | 0 | 0 | 0 |
| Heilongjiang (HLJ) | 178 | 0 | 0 | 0 | 0 | 0 | 0 | 0 | 0 |
| Jilin (JL) | 69 | 0 | 0 | 0 | 0 | 0 | 0 | 0 | 0 |
| Liaoning (LN) | 68 | 0 | 0 | 0 | 0 | 0 | 0 | 0 | 0 |
| Shandong (SD) | 90 | 0 | 0 | 0 | 0 | 0 | 0 | 0 | 0 |
| Jiangsu (JS) | 186 | 17 | 9.14 | 36 | 32 | 0 | 3 | 1 | 0 |
| Anhui (AH) | 365 | 22 | 6.03 | 53 | 25 | 0 | 22 | 2 | 4 |
| Zhejiang (ZJ) | 304 | 33 | 10.86 | 71 | 52 | 5 | 4 | 8 | 2 |
| Jiangxi (JX) | 222 | 20 | 9.01 | 53 | 48 | 0 | 3 | 2 | 0 |
| Fujian (FJ) | 205 | 8 | 3.9 | 28 | 24 | 0 | 3 | 0 | 1 |
| Shanghai (SH) | 68 | 6 | 8.82 | 16 | 16 | 0 | 0 | 0 | 0 |
| Henan (HeN) | 1001 | 88 | 8.79 | 220 | 146 | 11 | 33 | 22 | 8 |
| Hubei (HuB) | 187 | 16 | 8.56 | 33 | 30 | 0 | 3 | 0 | 0 |
| Hunan (HuN) | 217 | 31 | 14.29 | 136 | 79 | 5 | 22 | 25 | 5 |
| Guangdong (GD) | 220 | 17 | 7.73 | 126 | 68 | 0 | 22 | 25 | 11 |
| Guangxi (GX) | 310 | 77 | 24.84 | 318 | 216 | 19 | 48 | 30 | 5 |
| Hainan (HaN) | 71 | 11 | 15.49 | 36 | 25 | 2 | 5 | 3 | 1 |
| Sichuan (SC) | 216 | 51 | 23.61 | 234 | 154 | 8 | 28 | 32 | 12 |
| Yunnan (YN) | 235 | 16 | 6.81 | 40 | 28 | 2 | 6 | 4 | 0 |
| Guizhou (GZ) | 105 | 16 | 15.24 | 140 | 105 | 0 | 28 | 1 | 6 |
| Chongqing (CQ) | 176 | 18 | 10.23 | 53 | 37 | 3 | 8 | 4 | 1 |
| Qinghai (QH) | 7 | 0 | 0 | 0 | 0 | 0 | 0 | 0 | 0 |
| Shaanxi (SaX) | 42 | 0 | 0 | 0 | 0 | 0 | 0 | 0 | 0 |
| Ningxia (NX) | 31 | 0 | 0 | 0 | 0 | 0 | 0 | 0 | 0 |
