# Supplementary material for: Large-scale survey of a neglected agent of sparganosis Spirometra erinaceieuropaei (Cestoda: Diphyllobothriidae) in wild frogs in China
Source: PLoS Negl Trop Dis. 2020 Feb 26;14(2):e0008019. doi: 10.1371/journal.pntd.0008019 (PMC7043720; doi:10.1371/journal.pntd.0008019)
Supplement: S1 Fig — (DOC) [file pntd.0008019.s005.doc]

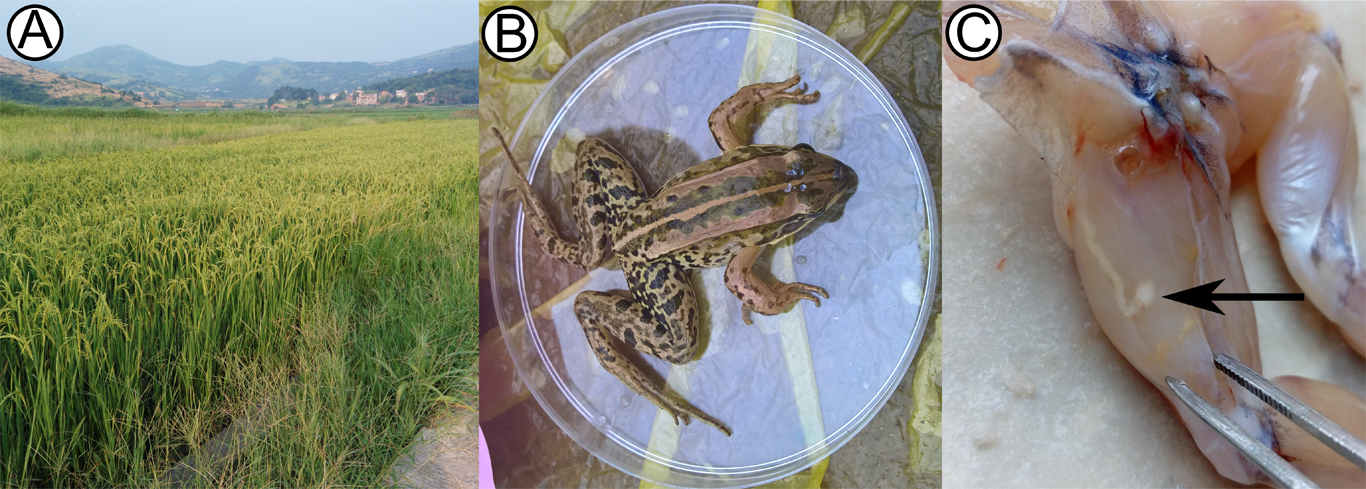


**S1 Fig.** The collection locations and the presence of sparganum parasitization in frogs. (A) The paddy field where frogs were collected. (B) The collected frog. (C) The sparganum parasitizing in the frog.
