# Supplementary material for: Large-scale survey of a neglected agent of sparganosis Spirometra erinaceieuropaei (Cestoda: Diphyllobothriidae) in wild frogs in China
Source: PLoS Negl Trop Dis. 2020 Feb 26;14(2):e0008019. doi: 10.1371/journal.pntd.0008019 (PMC7043720; doi:10.1371/journal.pntd.0008019)
Supplement: S2 Fig — (DOC) [file pntd.0008019.s006.doc]

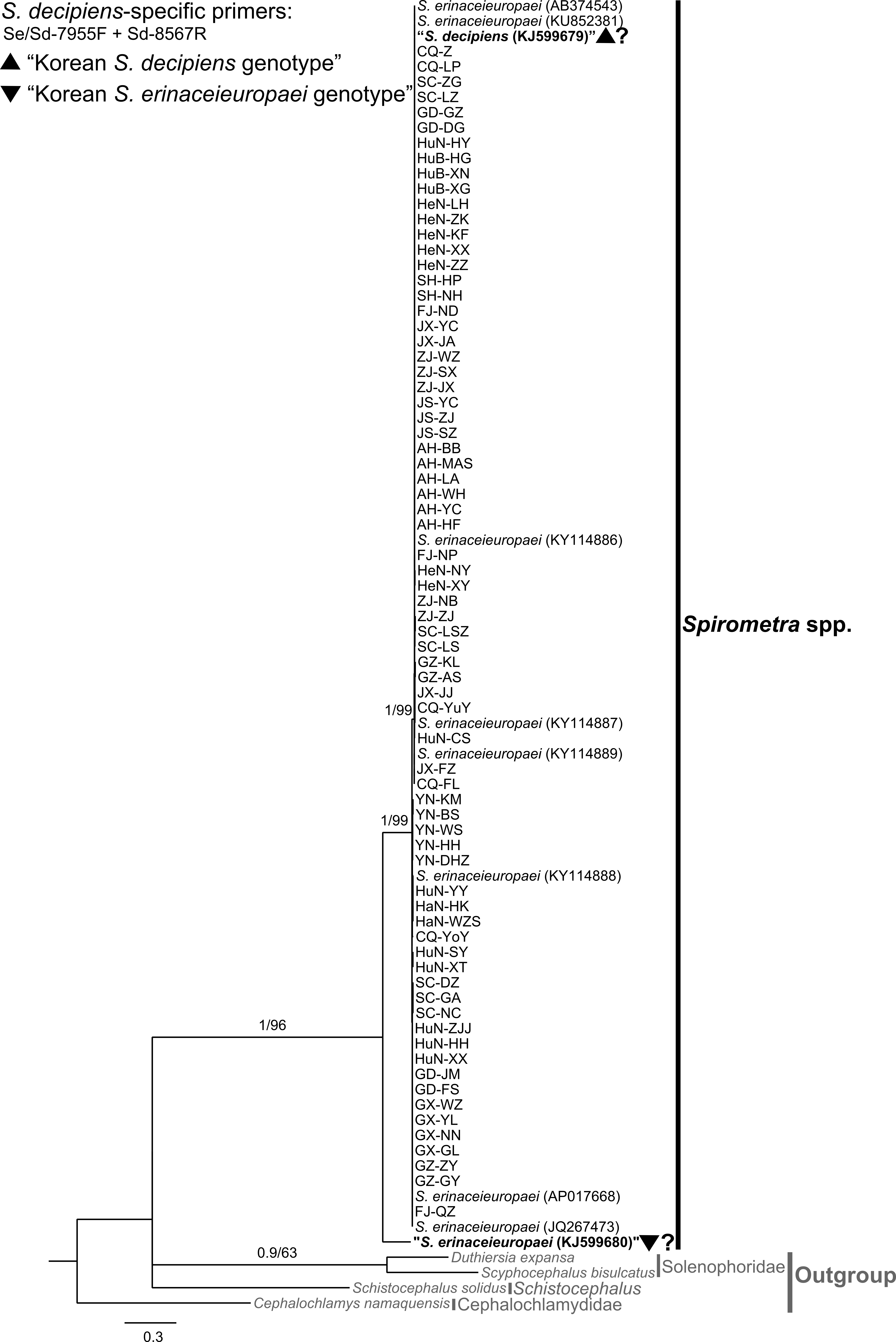


**S2 Fig.** The maximum likelihood and Bayesian tree based on the sequences of the PCR products amplified with the primers Se/Sd-7955F + Sd-8567R.
