# Supplementary material for: Large-scale survey of a neglected agent of sparganosis Spirometra erinaceieuropaei (Cestoda: Diphyllobothriidae) in wild frogs in China
Source: PLoS Negl Trop Dis. 2020 Feb 26;14(2):e0008019. doi: 10.1371/journal.pntd.0008019 (PMC7043720; doi:10.1371/journal.pntd.0008019)
Supplement: S1 Dataset — (DOC) [file pntd.0008019.s007.doc]

**S1 Dataset.** The sequencing data of the sparganum isolates used in this study.

Sequenced PCR products using primers Se/Sd-1800F+Sd-2317R:

>AH_YC

TGGCTCATTTTCATTATGTTATGTCTTTGGGTTCTTATATTAGGGTTATTATATTTTTTGTTTGGTGATGGCCTGTTATCACAGGGGTTAGCTTGAATAAGTATTTGTTACAGTGTCATTGTATAGTATCAAATGTGGGCTTTAATTTGTGTTTTTTTCCTATGCATTATTTTGGTATTTGTGGTTTACCTCGGCGTGTTTGTGTGTATGAGTCAGGGTACGCTTGAGTTAATATGCTTTGTTCAATAGGTTCTTTTGTTTCTGCCTTTAGTGGTTGCTTTTTTATTTTTATTTTATGGGAGTCTTTAGCTAAAAAGAATGTTGTTATAGGTTATTATGGTAGTTCTTCAACTTTGCTTAATTTGTGTTGATCGCCAGTGCCTTACCACAGTAATTTTTTTGTGCGTGGATTATTTGTTGATTATTCTGTATTGGCTTTTTAGTTTATTGTTTAGAATATTGATTTTGTAAATCAGGGGTAGGGTGTTTTGACCTTTAAGCCTGTTTGGTGGAACTTTTAATTGGGTTGATTGGTTTTAATTGCCTTTTGCATCATGCTATATGGATGTGGTTAGTATATGGGCTATACACCGAAAAGTTTGGATCTAGTATCAAGTTGGTT

>AH_HF

TGGCTCATTTTCATTATGTTATGTCTTTGGGTTCTTATATTAGGGTTATTATATTTTTTGTTTGGTGATGGCCTGTTATCACAGGGGTTAGCTTGAATAAGTATTTGTTACAGTGTCATTGTATAGTATCAAATGTGGGCTTTAATTTGTGTTTTTTTCCTATGCATTATTTTGGTATTTGTGGTTTACCTCGGCGTGTTTGTGTGTATGAGTCAGGGTACGCTTGAGTTAATATGCTTTGTTCAATAGGTTCTTTTGTTTCTGCCTTTAGTGGTTGCTTTTTTATTTTTATTTTATGGGAGTCTTTAGCTAAAAAGAATGTTGTTATAGGTTATTATGGTAGTTCTTCAACTTTGCTTAATTTGTGTTGATCGCCAGTGCCTTACCACAGTAATTTTTTTGTGCGTGGATTATTTGTTGATTATTCTGTATTGGCTTTTTAGTTTATTGTTTAGAATATTGATTTTGTAAATCAGGGGTAGGGTGTTTTGACCTTTAAGCCTGTTTGGTGGAACTTTTAATTGGGTTGATTGGTTTTAATTGCCTTTTGCATCATGCTATATGGATGTGGTTAGTATATGGGCTATACACCGAAAAGTTTGGATCTAGTATCAAGTTGGTT

>AH_WH

TGGCTCATTTTCATTATGTTATGTCTTTGGGTTCTTATATTAGGGTTATTATATTTTTTGTTTGGTGATGGCCTGTTATCACAGGGGTTAGCTTGAATAAGTATTTGTTACAGTGTCATTGTATAGTATCAAATGTGGGCTTTAATTTGTGTTTTTTTCCTATGCATTATTTTGGTATTTGTGGTTTACCTCGGCGTGTTTGTGTGTATGAGTCAGGGTACGCTTGAGTTAATATGCTTTGTTCAATAGGTTCTTTTGTTTCTGCCTTTAGTGGTTGCTTTTTTATTTTTATTTTATGGGAGTCTTTAGCTAAAAAGAATGTTGTTATAGGTTATTATGGTAGTTCTTCAACTTTGCTTAATTTGTGTTGATCGCCAGTGCCTTACCACAGTAATTTTTTTGTGCGTGGATTATTTGTTGATTATTCTGTATTGGCTTTTTAGTTTATTGTTTAGAATATTGATTTTGTAAATCAGGGGTAGGGTGTTTTGACCTTTAAGCCTGTTTGGTGGAACTTTTAATTGGGTTGATTGGTTTTAATTGCCTTTTGCATCATGCTATATGGATGTGGTTAGTATATGGGCTATACACCGAAAAGTTTGGATCTAGTATCAAGTTGGTT

>AH_LA

TGGCTCATTTTCATTATGTTATGTCTTTGGGTTCTTATATTAGGGTTATTATATTTTTTGTTTGGTGATGGCCTGTTATCACAGGGGTTAGCTTGAATAAGTATTTGTTACAGTGTCATTGTATAGTATCAAATGTGGGCTTTAATTTGTGTTTTTTTCCTATGCATTATTTTGGTATTTGTGGTTTACCTCGGCGTGTTTGTGTGTATGAGTCAGGGTACGCTTGAGTTAATATGCTTTGTTCAATAGGTTCTTTTGTTTCTGCCTTTAGTGGTTGCTTTTTTATTTTTATTTTATGGGAGTCTTTAGCTAAAAAGAATGTTGTTATAGGTTATTATGGTAGTTCTTCAACTTTGCTTAATTTGTGTTGATCGCCAGTGCCTTACCACAGTAATTTTTTTGTGCGTGGATTATTTGTTGATTATTCTGTATTGGCTTTTTAGTTTATTGTTTAGAATATTGATTTTGTAAATCAGGGGTAGGGTGTTTTGACCTTTAAGCCTGTTTGGTGGAACTTTTAATTGGGTTGATTGGTTTTAATTGCCTTTTGCATCATGCTATATGGATGTGGTTAGTATATGGGCTATACACCGAAAAGTTTGGATCTAGTATCAAGTTGGTT

>AH_MAS

TGGCTCATTTTCATTATGTTATGTCTTTGGGTTCTTATATTAGGGTTATTATATTTTTTGTTTGGTGATGGCCTGTTATCACAGGGGTTAGCTTGAATAAGTATTTGTTACAGTGTCATTGTATAGTATCAAATGTGGGCTTTAATTTGTGTTTTTTTCCTATGCATTATTTTGGTATTTGTGGTTTACCTCGGCGTGTTTGTGTGTATGAGTCAGGGTACGCTTGAGTTAATATGCTTTGTTCAATAGGTTCTTTTGTTTCTGCCTTTAGTGGTTGCTTTTTTATTTTTATTTTATGGGAGTCTTTAGCTAAAAAGAATGTTGTTATAGGTTATTATGGTAGTTCTTCAACTTTGCTTAATTTGTGTTGATCGCCAGTGCCTTACCACAGTAATTTTTTTGTGCGTGGATTATTTGTTGATTATTCTGTATTGGCTTTTTAGTTTATTGTTTAGAATATTGATTTTGTAAATCAGGGGTAGGGTGTTTTGACCTTTAAGCCTGTTTGGTGGAACTTTTAATTGGGTTGATTGGTTTTAATTGCCTTTTGCATCATGCTATATGGATGTGGTTAGTATATGGGCTATACACCGAAAAGTTTGGATCTAGTATCAAGTTGGTT

>AH_BB

TGGCTCATTTTCATTATGTTATGTCTTTGGGTTCTTATATTAGGGTTATTATATTTTTTGTTTGGTGATGGCCTGTTATCACAGGGGTTAGCTTGAATAAGTATTTGTTACAGTGTCATTGTATAGTATCAAATGTGGGCTTTAATTTGTGTTTTTTTCCTATGCATTATTTTGGTATTTGTGGTTTACCTCGGCGTGTTTGTGTGTATGAGTCAGGGTACGCTTGAGTTAATATGCTTTGTTCAATAGGTTCTTTTGTTTCTGCCTTTAGTGGTTGCTTTTTTATTTTTATTTTATGGGAGTCTTTAGCTAAAAAGAATGTTGTTATAGGTTATTATGGTAGTTCTTCAACTTTGCTTAATTTGTGTTGATCGCCAGTGCCTTACCACAGTAATTTTTTTGTGCGTGGATTATTTGTTGATTATTCTGTATTGGCTTTTTAGTTTATTGTTTAGAATATTGATTTTGTAAATCAGGGGTAGGGTGTTTTGACCTTTAAGCCTGTTTGGTGGAACTTTTAATTGGGTTGATTGGTTTTAATTGCCTTTTGCATCATGCTATATGGATGTGGTTAGTATATGGGCTATACACCGAAAAGTTTGGATCTAGTATCAAGTTGGTT

>JS_SZ

TGGCTCATTTTCATTATGTTATGTCTTTGGGTTCTTATATTAGGGTTATTATATTTTTTGTTTGGTGATGGCCTGTTATCACAGGGGTTAGCTTGAATAAGTATTTGTTACAGTGTCATTGTATAGTATCAAATGTGGGCTTTAATTTGTGTTTTTTTCCTATGCATTATTTTGGTATTTGTGGTTTACCTCGGCGTGTTTGTGTGTATGAGTCAGGGTACGCTTGAGTTAATATGCTTTGTTCAATAGGTTCTTTTGTTTCTGCCTTTAGTGGTTGCTTTTTTATTTTTATTTTATGGGAGTCTTTAGCTAAAAAGAATGTTGTTATAGGTTATTATGGTAGTTCTTCAACTTTGCTTAATTTGTGTTGATCGCCAGTGCCTTACCACAGTAATTTTTTTGTGCGTGGATTATTTGTTGATTATTCTGTATTGGCTTTTTAGTTTATTGTTTAGAATATTGATTTTGTAAATCAGGGGTAGGGTGTTTTGACCTTTAAGCCTGTTTGGTGGAACTTTTAATTGGGTTGATTGGTTTTAATTGCCTTTTGCATCATGCTATATGGATGTGGTTAGTATATGGGCTATACACCGAAAAGTTTGGATCTAGTATCAAGTTGGTT

>JS_ZJ

TGGCTCATTTTCATTATGTTATGTCTTTGGGTTCTTATATTAGGGTTATTATATTTTTTGTTTGGTGATGGCCTGTTATCACAGGGGTTAGCTTGAATAAGTATTTGTTACAGTGTCATTGTATAGTATCAAATGTGGGCTTTAATTTGTGTTTTTTTCCTATGCATTATTTTGGTATTTGTGGTTTACCTCGGCGTGTTTGTGTGTATGAGTCAGGGTACGCTTGAGTTAATATGCTTTGTTCAATAGGTTCTTTTGTTTCTGCCTTTAGTGGTTGCTTTTTTATTTTTATTTTATGGGAGTCTTTAGCTAAAAAGAATGTTGTTATAGGTTATTATGGTAGTTCTTCAACTTTGCTTAATTTGTGTTGATCGCCAGTGCCTTACCACAGTAATTTTTTTGTGCGTGGATTATTTGTTGATTATTCTGTATTGGCTTTTTAGTTTATTGTTTAGAATATTGATTTTGTAAATCAGGGGTAGGGTGTTTTGACCTTTAAGCCTGTTTGGTGGAACTTTTAATTGGGTTGATTGGTTTTAATTGCCTTTTGCATCATGCTATATGGATGTGGTTAGTATATGGGCTATACACCGAAAAGTTTGGATCTAGTATCAAGTTGGTT

>JS_YC

TGGCTCATTTTCATTATGTTATGTCTTTGGGTTCTTATATTAGGGTTATTATATTTTTTGTTTGGTGATGGCCTGTTATCACAGGGGTTAGCTTGAATAAGTATTTGTTACAGTGTCATTGTATAGTATCAAATGTGGGCTTTAATTTGTGTTTTTTTCCTATGCATTATTTTGGTATTTGTGGTTTACCTCGGCGTGTTTGTGTGTATGAGTCAGGGTACGCTTGAGTTAATATGCTTTGTTCAATAGGTTCTTTTGTTTCTGCCTTTAGTGGTTGCTTTTTTATTTTTATTTTATGGGAGTCTTTAGCTAAAAAGAATGTTGTTATAGGTTATTATGGTAGTTCTTCAACTTTGCTTAATTTGTGTTGATCGCCAGTGCCTTACCACAGTAATTTTTTTGTGCGTGGATTATTTGTTGATTATTCTGTATTGGCTTTTTAGTTTATTGTTTAGAATATTGATTTTGTAAATCAGGGGTAGGGTGTTTTGACCTTTAAGCCTGTTTGGTGGAACTTTTAATTGGGTTGATTGGTTTTAATTGCCTTTTGCATCATGCTATATGGATGTGGTTAGTATATGGGCTATACACCGAAAAGTTTGGATCTAGTATCAAGTTGGTT

>ZJ_JX

TGGCTCATTTTCATTATGTTATGTCTTTGGGTTCTTATATTAGGGTTATTATATTTTTTGTTTGGTGATGGCCTGTTATCACAGGGGTTAGCTTGAATAAGTATTTGTTACAGTGTCATTGTATAGTATCAAATGTGGGCTTTAATTTGTGTTTTTTTCCTATGCATTATTTTGGTATTTGTGGTTTACCTCGGCGTGTTTGTGTGTATGAGTCAGGGTACGCTTGAGTTAATATGCTTTGTTCAATAGGTTCTTTTGTTTCTGCCTTTAGTGGTTGCTTTTTTATTTTTATTTTATGGGAGTCTTTAGCTAAAAAGAATGTTGTTATAGGTTATTATGGTAGTTCTTCAACTTTGCTTAATTTGTGTTGATCGCCAGTGCCTTACCACAGTAATTTTTTTGTGCGTGGATTATTTGTTGATTATTCTGTATTGGCTTTTTAGTTTATTGTTTAGAATATTGATTTTGTAAATCAGGGGTAGGGTGTTTTGACCTTTAAGCCTGTTTGGTGGAACTTTTAATTGGGTTGATTGGTTTTAATTGCCTTTTGCATCATGCTATATGGATGTGGTTAGTATATGGGCTATACACCGAAAAGTTTGGATCTAGTATCAAGTTGGTT

>ZJ_NB

TGGCTCATTTTCATTATGTTATGTCTTTGGGTTCTTATATTAGGGTTATTATATTTTTTGTTTGGTGATGGCCTGTTATCACAGGGGTTAGCTTGAATAAGTATTTGTTACAGTGTCATTGTATAGTATCAAATGTGGGCTTTAATTTGTGTTTTTTTCCTATGCATTATTTTGGTATTTGTGGTTTACCTCGGCGTGTTTGTGTGTATGAGTCAGGGTACGCTTGAGTTAATATGCTTTGTTCAATAGGTTCTTTTGTTTCTGCCTTTAGTGGTTGCTTTTTTATTTTTATTTTATGGGAGTCTTTAGCTAAAAAGAATGTTGTTATAGGTTATTATGGTAGTTCTTCAACTTTGCTTAATTTGTGTTGATCGCCAGTGCCTTACCACAGTAATTTTTTTGTGCGTGGATTATTTGTTGATTATTCTGTATTGGCTTTTTAGTTTATTGTTTAGAATATTGATTTTGTAAATCAGGGGTAGGGTGTTTTGACCTTTAAGCCTGTTTGGTGGAACTTTTAATTGGGTTGATTGGTTTTAATTGCCTTTTGCATCATGCTATGTGGATGTGGTTAGTATATGGGCTATACACCGAAAAGTTTGGATCTAGTATCAAGTTGGTT

>ZJ_SX

TGGCTCATTTTCATTATGTTATGTCTTTGGGTTCTTATATTAGGGTTATTATATTTTTTGTTTGGTGATGGCCTGTTATCACAGGGGTTAGCTTGAATAAGTATTTGTTACAGTGTCATTGTATAGTATCAAATGTGGGCTTTAATTTGTGTTTTTTTCCTATGCATTATTTTGGTATTTGTGGTTTACCTCGGCGTGTTTGTGTGTATGAGTCAGGGTACGCTTGAGTTAATATGCTTTGTTCAATAGGTTCTTTTGTTTCTGCCTTTAGTGGTTGCTTTTTTATTTTTATTTTATGGGAGTCTTTAGCTAAAAAGAATGTTGTTATAGGTTATTATGGTAGTTCTTCAACTTTGCTTAATTTGTGTTGATCGCCAGTGCCTTACCACAGTAATTTTTTTGTGCGTGGATTATTTGTTGATTATTCTGTATTGGCTTTTTAGTTTATTGTTTAGAATATTGATTTTGTAAATCAGGGGTAGGGTGTTTTGACCTTTAAGCCTGTTTGGTGGAACTTTTAATTGGGTTGATTGGTTTTAATTGCCTTTTGCATCATGCTATATGGATGTGGTTAGTATATGGGCTATACACCGAAAAGTTTGGATCTAGTATCAAGTTGGTT

>ZJ_WZ

TGGCTCATTTTCATTATGTTATGTCTTTGGGTTCTTATATTAGGGTTATTATATTTTTTGTTTGGTGATGGCCTGTTATCACAGGGGTTAGCTTGAATAAGTATTTGTTACAGTGTCATTGTATAGTATCAAATGTGGGCTTTAATTTGTGTTTTTTTCCTATGCATTATTTTGGTATTTGTGGTTTACCTCGGCGTGTTTGTGTGTATGAGTCAGGGTACGCTTGAGTTAATATGCTTTGTTCAATAGGTTCTTTTGTTTCTGCCTTTAGTGGTTGCTTTTTTATTTTTATTTTATGGGAGTCTTTAGCTAAAAAGAATGTTGTTATAGGTTATTATGGTAGTTCTTCAACTTTGCTTAATTTGTGTTGATCGCCAGTGCCTTACCACAGTAATTTTTTTGTGCGTGGATTATTTGTTGATTATTCTGTATTGGCTTTTTAGTTTATTGTTTAGAATATTGATTTTGTAAATCAGGGGTAGGGTGTTTTGACCTTTAAGCCTGTTTGGTGGAACTTTTAATTGGGTTGATTGGTTTTAATTGCCTTTTGCATCATGCTATATGGATGTGGTTAGTATATGGGCTATACACCGAAAAGTTTGGATCTAGTATCAAGTTGGTT

>ZJ_ZJ

TGGCTCATTTTCATTATGTTATGTCTTTGGGTTCTTATATTAGGGTTATTATATTTTTTGTTTGGTGATGGCCTGTTATCACAGGGGTTAGCTTGAATAAGTATTTGTTACAGTGTCATTGTATAGTATCAAATGTGGGCTTTAATTTGTGTTTTTTTCCTATGCATTATTTTGGTATTTGTGGTTTACCTCGGCGTGTTTGTGTGTATGAGTCAGGGTACGCTTGAGTTAATATGCTTTGTTCAATAGGTTCTTTTGTTTCTGCCTTTAGTGGTTGCTTTTTTATTTTTATTTTATGGGAGTCTTTAGCTAAAAAGAATGTTGTTATAGGTTATTATGGTAGTTCTTCAACTTTGCTTAATTTGTGTTGGTCGCCAGTGCCTTACCACAGTAATTTTTTTGTGCGTGGATTATTTGTTGATTATTCTGTATTGGCTTTTTAGTTTATTGTTTAGAATATTGATTTTGTAAATCAGGGGTAGGGTGTTTTGACCTTTAAGCCTGTTTGGTGGAACTTTTAATTGGGTTGATTGGTTTTAATTGCCTTTTGCATCATGCTATATGGATGTGGTTAGTATATGGGCTATAGACCGAAAAGTTTGGATCTAGTATCAAGTTGGTT

>JX_FZ

TGGCTCATTTTCATTATGTTATGTCTTTGGGTTCTTATATTAGGGTTATTATATTTTTTGTTTGGTGATGGCCTGTTATCACAGGGGTTAGCTTGAATAAGTATTTGTTACAGTGTCATTGTATAGTATCAAATGTGGGCTTTAATTTGTGTTTTTTTCCTATGCATTATTTTGGTATTTGTGGTTTACCTCGGCGTGTTTGTGTGTATGAGTCAGGGTACGCTTGAGTTAATATGCTTTGTTCAATAGGTTCTTTTGTTTCTGCCTTTAGTGGTTGCTTTTTTATTTTTATTTTATGGGAGTCTTTAGCTAAAAAGAATGTTGTTATAGGTTATTATGGTAGTTCTTCAACTTTGCTTAATTTGTGTTGATCGCCAGTGCCTTACCACAGTAATTTTTTTGTGCGCGGATTATTTGTTGATTATTCTGTATTGGCTTTTTAGTTTATTGTTTAGAATATTGATTTTGTAAATCAGGGGTAGGGTGTTTTGACCTTTAAGCCTGTTTGGTGGAACTTTTAATTGGGTTGATTGGTTTTAATTGCCTTTTGCATCATGCTATATGGATGTGGTTAGTATATGGGCTATACACCGAAAAGTTTGGATCTAGTATCAAGTTGGTT

>JX_JJ

TGGCTCATTTTCATTATGTTATGTCTTTGGGTTCTTATATTAGGGTTATTATATTTTTTGTTTGGTGATGGCCTGTTATCACAGGGGTTAGCTTGAATAAGTATTTGTTACAGTGTCATTGTATAGTATCAAATGTGGGCTTTAATTTGTGTTTTTTTCCTATGCATTATTTTGGTATTTGTGGTTTACCTCGGCGTGTTTGTGTGTATGAGTCAGGGTACGCTTGAGTTAATATGCTTTGTTCAATAGGTTCTTTTGTTTCTGCCTTTAGTGGTTGCTTTTTTATTTTTATTTTATGGGAGTCTTTAGCTAAAAAGAATGTTGTTATAGGTTATTATGGTAGTTCTTCAACTTTGCTTAATTTGTGTTGATCGCCAGTGCCTTACCACAGTAATTTTTTTGTGCGTGGATTATTTGTTGATTATTCTGTATTGGCTTTTTAGTTTATTGTTTAGAATATTGATTTTGTAAATCAGGGGTAGGGTGTTTTGACCTTTAAGCCTGTTTGGTGGAACTTTTAATTGGGTTGATTGGTTTTAATTGCCTTTTGCATCATGCTATATGGATGTGGTTAGTATATGGGCTATAGACCGAAAAGTTTGGATCTAGTATCAAGTTGGTT

>JX_JA

TGGCTCATTTTCATTATGTTATGTCTTTGGGTTCTTATATTAGGGTTATTATATTTTTTGTTTGGTGATGGCCTGTTATCACAGGGGTTAGCTTGAATAAGTATTTGTTACAGTGTCATTGTATAGTATCAAATGTGGGCTTTAATTTGTGTTTTTTTCCTATGCATTATTTTGGTATTTGTGGTTTACCTCGGCGTGTTTGTGTGTATGAGTCAGGGTACGCTTGAGTTAATATGCTTTGTTCAATAGGTTCTTTTGTTTCTGCCTTTAGTGGTTGCTTTTTTATTTTTATTTTATGGGAGTCTTTAGCTAAAAAGAATGTTGTTATAGGTTATTATGGTAGTTCTTCAACTTTGCTTAATTTGTGTTGATCGCCAGTGCCTTACCACAGTAATTTTTTTGTGCGTGGATTATTTGTTGATTATTCTGTATTGGCTTTTTAGTTTATTGTTTAGAATATTGATTTTGTAAATCAGGGGTAGGGTGTTTTGACCTTTAAGCCTGTTTGGTGGAACTTTTAATTGGGTTGATTGGTTTTAATTGCCTTTTGCATCATGCTATATGGATGTGGTTAGTATATGGGCTATACACCGAAAAGTTTGGATCTAGTATCAAGTTGGTT

>JX_YC

TGGCTCATTTTCATTATGTTATGTCTTTGGGTTCTTATATTAGGGTTATTATATTTTTTGTTTGGTGATGGCCTGTTATCACAGGGGTTAGCTTGAATAAGTATTTGTTACAGTGTCATTGTATAGTATCAAATGTGGGCTTTAATTTGTGTTTTTTTCCTATGCATTATTTTGGTATTTGTGGTTTACCTCGGCGTGTTTGTGTGTATGAGTCAGGGTACGCTTGAGTTAATATGCTTTGTTCAATAGGTTCTTTTGTTTCTGCCTTTAGTGGTTGCTTTTTTATTTTTATTTTATGGGAGTCTTTAGCTAAAAAGAATGTTGTTATAGGTTATTATGGTAGTTCTTCAACTTTGCTTAATTTGTGTTGATCGCCAGTGCCTTACCACAGTAATTTTTTTGTGCGTGGATTATTTGTTGATTATTCTGTATTGGCTTTTTAGTTTATTGTTTAGAATATTGATTTTGTAAATCAGGGGTAGGGTGTTTTGACCTTTAAGCCTGTTTGGTGGAACTTTTAATTGGGTTGATTGGTTTTAATTGCCTTTTGCATCATGCTATATGGATGTGGTTAGTATATGGGCTATACACCGAAAAGTTTGGATCTAGTATCAAGTTGGTT

>FJ_ND

TGGCTCATTTTCATTATGTTATGTCTTTGGGTTCTTATATTAGGGTTATTATATTTTTTGTTTGGTGATGGCCTGTTATCACAGGGGTTAGCTTGAATAAGTATTTGTTACAGTGTCATTGTATAGTATCAAATGTGGGCTTTAATTTGTGTTTTTTTCCTATGCATTATTTTGGTATTTGTGGTTTACCTCGGCGTGTTTGTGTGTATGAGTCAGGGTACGCTTGAGTTAATATGCTTTGTTCAATAGGTTCTTTTGTTTCTGCCTTTAGTGGTTGCTTTTTTATTTTTATTTTATGGGAGTCTTTAGCTAAAAAGAATGTTGTTATAGGTTATTATGGTAGTTCTTCAACTTTGCTTAATTTGTGTTGATCGCCAGTGCCTTACCACAGTAATTTTTTTGTGCGTGGATTATTTGTTGATTATTCTGTATTGGCTTTTTAGTTTATTGTTTAGAATATTGATTTTGTAAATCAGGGGTAGGGTGTTTTGACCTTTAAGCCTGTTTGGTGGAACTTTTAATTGGGTTGATTGGTTTTAATTGCCTTTTGCATCATGCTATATGGATGTGGTTAGTATATGGGCTATACACCGAAAAGTTTGGATCTAGTATCAAGTTGGTT

>FJ_QZ

TGGCTCATTTTCATTATGTTATGTCTTTGGGTTCTTATATTAGGATTATTATATTTTTTGTTTGGTGATGGCCTGTTATCACAGGGGTTAGCCTGAATAAGTATTTGTTACAGTGTCATTGTATAGTATCAAATGTGGGTTTTAATTTGTGTTTTTTCCCTATGCATTATTTTGGTATTTGTGGTTTACCTCGGCGTGTTTGTGTGTATGAGTCAGGGTACGCTTGAGTTAATATGCTTTGTTCAATAGGTTCTTTTGTTTCTGCCTTTAGTGGTTGCTTTTTTATTTTTATTTTATGGGAGTCTTTAGCTAAAAAGAATGTTGTTATAGGTTATTATGGTAGTTCTTCAACTTTGCTTAATCTGTGTTGGTCGCCAGTGCCTTATCACAGTAATTTTTTTGTGCGCGGATTATTTGTTGATTATTCTGTATTGGCTTTTTAGTTTATTGTTTAGAATATTGATTTTGTAAATCAGGGGTAGGGTGTTTTGACCTTTAAGCCTGTTTGGTGGAACTTTTAATTGGGTTGATTGGTTTTAATTGCCTTTTGCATCATGCTATATGGATGTGGTTAGTATATGGGCTATAGACCGAAAAGTTT---------------------

>FJ_NP

TGGCTCATTTTCATTATGTTATGTCTTTGGGTTCTTATATTAGGGTTATTATATTTTTTGTTTGGTGATGGCCTGTTATCACAGGGGTTAGCTTGAATAAGTATTTGTTACAGTGTCATTGTATAGTATCAAATGTGGGCTTTAATTTGTGTTTTTTTCCTATGCATTATTTTGGTATTTGTGGTTTACCTCGGCGTGTTTGTGTGTATGAGTCAGGGTACGCTTGAGTTAATATGCTTTGTTCAATAGGTTCTTTTGTTTCTGCCTTTAGTGGTTGCTTTTTTATTTTTATTTTATGGGAGTCTTTAGCTAAAAAGAATGTTGTTATAGGTTATTATGGTAGTTCTTCAACTTTGCTTAATTTGTGTTGATCGCCAGTGCCTTACCACAGTAATTTTTTTGTGCGTGGATTATTTGTTGATTATTCTGTATTGGCTTTTTAGTTTATTGTTTAGAATATTGATTTTGTAAATCAGGGGTAGGGTGTTTTGACCTTTAAGCCTGTTTGGTGGAACTTTTAATTGGGTTGATTGGTTTTAATTGCCTTTTGCATCATGCTATATGGATGTGGTTAGTATATGGGCTATACACCGAAAAGTTT---------------------

>SH_NH

TGGCTCATTTTCATTATGTTATGTCTTTGGGTTCTTATATTAGGGTTATTATATTTTTTGTTTGGTGATGGCCTGTTATCACAGGGGTTAGCTTGAATAAGTATTTGTTACAGTGTCATTGTATAGTATCAAATGTGGGCTTTAATTTGTGTTTTTTTCCTATGCATTATTTTGGTATTTGTGGTTTACCTCGGCGTGTTTGTGTGTATGAGTCAGGGTACGCTTGAGTTAATATGCTTTGTTCAATAGGTTCTTTTGTTTCTGCCTTTAGTGGTTGCTTTTTTATTTTTATTTTATGGGAGTCTTTAGCTAAAAAGAATGTTGTTATAGGTTATTATGGTAGTTCTTCAACTTTGCTTAATTTGTGTTGATCGCCAGTGCCTTACCACAGTAATTTTTTTGTGCGTGGATTATTTGTTGATTATTCTGTATTGGCTTTTTAGTTTATTGTTTAGAATATTGATTTTGTAAATCAGGGGTAGGGTGTTTTGACCTTTAAGCCTGTTTGGTGGAACTTTTAATTGGGTTGATTGGTTTTAATTGCCTTTTGCATCATGCTATATGGATGTGGTTAGTATATGGGCTATACACCGAAAAGTTTGGATCTAGTATCAAGTTGGTT

>SH_HP

TGGCTCATTTTCATTATGTTATGTCTTTGGGTTCTTATATTAGGGTTATTATATTTTTTGTTTGGTGATGGCCTGTTATCACAGGGGTTAGCTTGAATAAGTATTTGTTACAGTGTCATTGTATAGTATCAAATGTGGGCTTTAATTTGTGTTTTTTTCCTATGCATTATTTTGGTATTTGTGGTTTACCTCGGCGTGTTTGTGTGTATGAGTCAGGGTACGCTTGAGTTAATATGCTTTGTTCAATAGGTTCTTTTGTTTCTGCCTTTAGTGGTTGCTTTTTTATTTTTATTTTATGGGAGTCTTTAGCTAAAAAGAATGTTGTTATAGGTTATTATGGTAGTTCTTCAACTTTGCTTAATTTGTGTTGATCGCCAGTGCCTTACCACAGTAATTTTTTTGTGCGTGGATTATTTGTTGATTATTCTGTATTGGCTTTTTAGTTTATTGTTTAGAATATTGATTTTGTAAATCAGGGGTAGGGTGTTTTGACCTTTAAGCCTGTTTGGTGGAACTTTTAATTGGGTTGATTGGTTTTAATTGCCTTTTGCATCATGCTATATGGATGTGGTTAGTATATGGGCTATACACCGAAAAGTTTGGATCTAGTATCAAGTTGGTT

>HeN_ZZ

TGGCTCATTTTCATTATGTTATGTCTTTGGGTTCTTATATTAGGGTTATTATATTTTTTGTTTGGTGATGGCCTGTTATCACAGGGGTTAGCTTGAATAAGTATTTGTTACAGTGTCATTGTATAGTATCAAATGTGGGCTTTAATTTGTGTTTTTTTCCTATGCATTATTTTGGTATTTGTGGTTTACCTCGGCGTGTTTGTGTGTATGAGTCAGGGTACGCTTGAGTTAATATGCTTTGTTCAATAGGTTCTTTTGTTTCTGCCTTTAGTGGTTGCTTTTTTATTTTTATTTTATGGGAGTCTTTAGCTAAAAAGAATGTTGTTATAGGTTATTATGGTAGTTCTTCAACTTTGCTTAATTTGTGTTGATCGCCAGTGCCTTACCACAGTAATTTTTTTGTGCGTGGATTATTTGTTGATTATTCTGTATTGGCTTTTTAGTTTATTGTTTAGAATATTGATTTTGTAAATCAGGGGTAGGGTGTTTTGACCTTTAAGCCTGTTTGGTGGAACTTTTAATTGGGTTGATTGGTTTTAATTGCCTTTTGCATCATGCTATATGGATGTGGTTAGTATATGGGCTATACACCGAAAAGTTTGGATCTAGTATCAAGTTGGTT

>HeN_XX

TGGCTCATTTTCATTATGTTATGTCTTTGGGTTCTTATATTAGGGTTATTATATTTTTTGTTTGGTGATGGCCTGTTATCACAGGGGTTAGCTTGAATAAGTATTTGTTACAGTGTCATTGTATAGTATCAAATGTGGGCTTTAATTTGTGTTTTTTTCCTATGCATTATTTTGGTATTTGTGGTTTACCTCGGCGTGTTTGTGTGTATGAGTCAGGGTACGCTTGAGTTAATATGCTTTGTTCAATAGGTTCTTTTGTTTCTGCCTTTAGTGGTTGCTTTTTTATTTTTATTTTATGGGAGTCTTTAGCTAAAAAGAATGTTGTTATAGGTTATTATGGTAGTTCTTCAACTTTGCTTAATTTGTGTTGATCGCCAGTGCCTTACCACAGTAATTTTTTTGTGCGTGGATTATTTGTTGATTATTCTGTATTGGCTTTTTAGTTTATTGTTTAGAATATTGATTTTGTAAATCAGGGGTAGGGTGTTTTGACCTTTAAGCCTGTTTGGTGGAACTTTTAATTGGGTTGATTGGTTTTAATTGCCTTTTGCATCATGCTATATGGATGTGGTTAGTATATGGGCTATACACCGAAAAGTTTGGATCTAGTATCAAGTTGGTT

>HeN_KF

TGGCTCATTTTCATTATGTTATGTCTTTGGGTTCTTATATTAGGGTTATTATATTTTTTGTTTGGTGATGGCCTGTTATCACAGGGGTTAGCTTGAATAAGTATTTGTTACAGTGTCATTGTATAGTATCAAATGTGGGCTTTAATTTGTGTTTTTTTCCTATGCATTATTTTGGTATTTGTGGTTTACCTCGGCGTGTTTGTGTGTATGAGTCAGGGTACGCTTGAGTTAATATGCTTTGTTCAATAGGTTCTTTTGTTTCTGCCTTTAGTGGTTGCTTTTTTATTTTTATTTTATGGGAGTCTTTAGCTAAAAAGAATGTTGTTATAGGTTATTATGGTAGTTCTTCAACTTTGCTTAATTTGTGTTGATCGCCAGTGCCTTACCACAGTAATTTTTTTGTGCGTGGATTATTTGTTGATTATTCTGTATTGGCTTTTTAGTTTATTGTTTAGAATATTGATTTTGTAAATCAGGGGTAGGGTGTTTTGACCTTTAAGCCTGTTTGGTGGAACTTTTAATTGGGTTGATTGGTTTTAATTGCCTTTTGCATCATGCTATATGGATGTGGTTAGTATATGGGCTATACACCGAAAAGTTTGGATCTAGTATCAAGTTGGTT

>HeN_ZK

TGGCTCATTTTCATTATGTTATGTCTTTGGGTTCTTATATTAGGGTTATTATATTTTTTGTTTGGTGATGGCCTGTTATCACAGGGGTTAGCTTGAATAAGTATTTGTTACAGTGTCATTGTATAGTATCAAATGTGGGCTTTAATTTGTGTTTTTTTCCTATGCATTATTTTGGTATTTGTGGTTTACCTCGGCGTGTTTGTGTGTATGAGTCAGGGTACGCTTGAGTTAATATGCTTTGTTCAATAGGTTCTTTTGTTTCTGCCTTTAGTGGTTGCTTTTTTATTTTTATTTTATGGGAGTCTTTAGCTAAAAAGAATGTTGTTATAGGTTATTATGGTAGTTCTTCAACTTTGCTTAATTTGTGTTGATCGCCAGTGCCTTACCACAGTAATTTTTTTGTGCGTGGATTATTTGTTGATTATTCTGTATTGGCTTTTTAGTTTATTGTTTAGAATATTGATTTTGTAAATCAGGGGTAGGGTGTTTTGACCTTTAAGCCTGTTTGGTGGAACTTTTAATTGGGTTGATTGGTTTTAATTGCCTTTTGCATCATGCTATATGGATGTGGTTAGTATATGGGCTATACACCGAAAAGTTTGGATCTAGTATCAAGTTGGTT

>HeN_LH

TGGCTCATTTTCATTATGTTATGTCTTTGGGTTCTTATATTAGGGTTATTATATTTTTTGTTTGGTGATGGCCTGTTATCACAGGGGTTAGCTTGAATAAGTATTTGTTACAGTGTCATTGTATAGTATCAAATGTGGGCTTTAATTTGTGTTTTTTTCCTATGCATTATTTTGGTATTTGTGGTTTACCTCGGCGTGTTTGTGTGTATGAGTCAGGGTACGCTTGAGTTAATATGCTTTGTTCAATAGGTTCTTTTGTTTCTGCCTTTAGTGGTTGCTTTTTTATTTTTATTTTATGGGAGTCTTTAGCTAAAAAGAATGTTGTTATAGGTTATTATGGTAGTTCTTCAACTTTGCTTAATTTGTGTTGATCGCCAGTGCCTTACCACAGTAATTTTTTTGTGCGTGGATTATTTGTTGATTATTCTGTATTGGCTTTTTAGTTTATTGTTTAGAATATTGATTTTGTAAATCAGGGGTAGGGTGTTTTGACCTTTAAGCCTGTTTGGTGGAACTTTTAATTGGGTTGATTGGTTTTAATTGCCTTTTGCATCATGCTATATGGATGTGGTTAGTATATGGGCTATACACCGAAAAGTTTGGATCTAGTATCAAGTTGGTT

>HeN_NY

TGGCTCATTTTCATTATGTTATGTCTTTGGGTTCTTATATTAGGGTTATTATATTTTTTGTTTGGTGATGGCCTGTTATCACAGGGGTTAGCTTGAATAAGTATTTGTTACAGTGTCATTGTATAGTATCAAATGTGGGCTTTAATTTGTGTTTTTTTCCTATGCATTATTTTGGTATTTGTGGTTTACCTCGGCGTGTTTGTGTGTATGAGTCAGGGTACGCTTGAGTTAATATGCTTTGTTCAATAGGTTCTTTTGTTTCTGCCTTTAGTGGTTGCTTTTTTATTTTTATTTTATGGGAGTCTTTAGCTAAAAAGAATGTTGTTATAGGTTATTATGGTAGTTCTTCAACTTTGCTTAATTTGTGTTGATCGCCAGTGCCTTACCACAGTAATTTTTTTGTGCGTGGATTATTTGTTGATTATTCTGTATTGGCTTTTTAGTTTATTGTTTAGAATATTGATTTTGTAAATCAGGGGTAGGGTGTTTTGACCTTTAAGCCTGTTTGGTGGAACTTTTAATTGGGTTGATTGGTTTTAATTGCCTTTTGCATCATGCTATATGGATGTGGTTAGTATATGGGCTATACACCGAAAAGTTTGGATCTAGTATCAAGTTGGGT

>HeN_XY

TGGCTCATTTTCATTATGTTATGTCTTTGGGTTCTTATATTAGGGTTATTATATTTTTTGTTTGGTGATGGCCTGTTATCACAGGGGTTAGCTTGAATAAGTATTTGTTACAGTGTCATTGTATAGTATCAAATGTGGGCTTTAATTTGTGTTTTTTTCCTATGCATTATTTTGGTATTTGTGGTTTACCTCGGCGTGTTTGTGTGTATGAGTCAGGGTACGCTTGAGTTAATATGCTTTGTTCAATAGGTTCTTTTGTTTCTGCCTTTAGTGGTTGCTTTTTTATTTTTATTTTATGGGAGTCTTTAGCTAAAAAGAATGTTGTTATAGGTTATTATGGTAGTTCTTCAACTTTGCTTAATTTGTGTTGATCGCCAGTGCCTTACCACAGTAATTTTTTTGTGCGTGGATTATTTGTTGATTATTCTGTATTGGCTTTTTAGTTTATTGTTTAGAATATTGATTTTGTAAATCAGGGGTAGGGTGTTTTGACCTTTAAGCCTGTTTGGTGGAACTTTTAATTGGGTTGATTGGTTTTAATTGCCTTTTGCATCATGCTATATGGATGTGGTTAGTATATGGGCTATACACCGAAAAGTTTGGATCTAGTATCAAGTTGGGT

>HuB_XG

TGGCTCATTTTCATTATGTTATGTCTTTGGGTTCTTATATTAGGGTTATTATATTTTTTGTTTGGTGATGGCCTGTTATCACAGGGGTTAGCTTGAATAAGTATTTGTTACAGTGTCATTGTATAGTATCAAATGTGGGCTTTAATTTGTGTTTTTTTCCTATGCATTATTTTGGTATTTGTGGTTTACCTCGGCGTGTTTGTGTGTATGAGTCAGGGTACGCTTGAGTTAATATGCTTTGTTCAATAGGTTCTTTTGTTTCTGCCTTTAGTGGTTGCTTTTTTATTTTTATTTTATGGGAGTCTTTAGCTAAAAAGAATGTTGTTATAGGTTATTATGGTAGTTCTTCAACTTTGCTTAATTTGTGTTGATCGCCAGTGCCTTACCACAGTAATTTTTTTGTGCGTGGATTATTTGTTGATTATTCTGTATTGGCTTTTTAGTTTATTGTTTAGAATATTGATTTTGTAAATCAGGGGTAGGGTGTTTTGACCTTTAAGCCTGTTTGGTGGAACTTTTAATTGGGTTGATTGGTTTTAATTGCCTTTTGCATCATGCTATATGGATGTGGTTAGTATATGGGCTATACACCGAAAAGTTTGGATCTAGTATCAAGTTGGTT

>HuB_XN

TGGCTCATTTTCATTATGTTATGTCTTTGGGTTCTTATATTAGGGTTATTATATTTTTTGTTTGGTGATGGCCTGTTATCACAGGGGTTAGCTTGAATAAGTATTTGTTACAGTGTCATTGTATAGTATCAAATGTGGGCTTTAATTTGTGTTTTTTTCCTATGCATTATTTTGGTATTTGTGGTTTACCTCGGCGTGTTTGTGTGTATGAGTCAGGGTACGCTTGAGTTAATATGCTTTGTTCAATAGGTTCTTTTGTTTCTGCCTTTAGTGGTTGCTTTTTTATTTTTATTTTATGGGAGTCTTTAGCTAAAAAGAATGTTGTTATAGGTTATTATGGTAGTTCTTCAACTTTGCTTAATTTGTGTTGATCGCCAGTGCCTTACCACAGTAATTTTTTTGTGCGTGGATTATTTGTTGATTATTCTGTATTGGCTTTTTAGTTTATTGTTTAGAATATTGATTTTGTAAATCAGGGGTAGGGTGTTTTGACCTTTAAGCCTGTTTGGTGGAACTTTTAATTGGGTTGATTGGTTTTAATTGCCTTTTGCATCATGCTATATGGATGTGGTTAGTATATGGGCTATACACCGAAAAGTTTGGATCTAGTATCAAGTTGGTT

>HuB_HG

TGGCTCATTTTCATTATGTTATGTCTTTGGGTTCTTATATTAGGGTTATTATATTTTTTGTTTGGTGATGGCCTGTTATCACAGGGGTTAGCTTGAATAAGTATTTGTTACAGTGTCATTGTATAGTATCAAATGTGGGCTTTAATTTGTGTTTTTTTCCTATGCATTATTTTGGTATTTGTGGTTTACCTCGGCGTGTTTGTGTGTATGAGTCAGGGTACGCTTGAGTTAATATGCTTTGTTCAATAGGTTCTTTTGTTTCTGCCTTTAGTGGTTGCTTTTTTATTTTTATTTTATGGGAGTCTTTAGCTAAAAAGAATGTTGTTATAGGTTATTATGGTAGTTCTTCAACTTTGCTTAATTTGTGTTGATCGCCAGTGCCTTACCACAGTAATTTTTTTGTGCGTGGATTATTTGTTGATTATTCTGTATTGGCTTTTTAGTTTATTGTTTAGAATATTGATTTTGTAAATCAGGGGTAGGGTGTTTTGACCTTTAAGCCTGTTTGGTGGAACTTTTAATTGGGTTGATTGGTTTTAATTGCCTTTTGCATCATGCTATATGGATGTGGTTAGTATATGGGCTATACACCGAAAAGTTTGGATCTAGTATCAAGTTGGTT

>HuN_ZJJ

TGGCTCATTTTCATTATGTTATGTCTTTGGGTTCTTATATTAGGATTATTATATTTTTTGTTTGGTGATGGCCTGTTATCACAGGGGTTAGCCTGAATAAGTATTTGTTACAGTGTCATTGTATAGTATCAAATGTGGGTTTTAATTTGTGTTTTTTCCCTATGCATTATTTTGGTATTTGTGGTTTACCTCGGCGTGTTTGTGTGTATGAGTCAGGGTACGCTTGAGTTAATATGCTTTGTTCAATAGGTTCTTTTGTTTCTGCCTTTAGTGGTTGCTTTTTTATTTTTATTTTATGGGAGTCTTTAGCTAAAAAGAATGTTGTTATAGGTTATTATGGTAGTTCTTCAACTTTGCTTAATCTGTGTTGGTCGCCAGTGCCTTATCACAGTAATTTTTTTGTGCGCGGATTATTTGTTGATTATTCTGTATTGGCTTTTTAGTTTATTGTTTAGAATATTGATTTTGTAAATCAGGGGTAGGGTGTTTTGACCTTTAAGCCTGTTTGGTGGAACTTTTAATTGGGTTGATTGGTTTTAATTGCCTTTTGCATCATGCTATATGGATGTGGTTAGTATATGGGCTATAGACCGAAAAGTTTGGATCTAGTATCAAGTTGGTT

>HuN_HH

TGGCTCATTTTCATTATGTTATGTCTTTGGGTTCTTATATTAGGATTATTATATTTTTTGTTTGGTGATGGCCTGTTATCACAGGGGTTAGCCTGAATAAGTATTTGTTACAGTGTCATTGTATAGTATCAAATGTGGGTTTTAATTTGTGTTTTTTCCCTATGCATTATTTTGGTATTTGTGGTTTACCTCGGCGTGTTTGTGTGTATGAGTCAGGGTACGCTTGAGTTAATATGCTTTGTTCAATAGGTTCTTTTGTTTCTGCCTTTAGTGGTTGCTTTTTTATTTTTATTTTATGGGAGTCTTTAGCTAAAAAGAATGTTGTTATAGGTTATTATGGTAGTTCTTCAACTTTGCTTAATCTGTGTTGGTCGCCAGTGCCTTATCACAGTAATTTTTTTGTGCGCGGATTATTTGTTGATTATTCTGTATTGGCTTTTTAGTTTATTGTTTAGAATATTGATTTTGTAAATCAGGGGTAGGGTGTTTTGACCTTTAAGCCTGTTTGGTGGAACTTTTAATTGGGTTGATTGGTTTTAATTGCCTTTTGCATCATGCTATATGGATGTGGTTAGTATATGGGCTATAGACCGAAAAGTTTGGATCTAGTATCAAGTTGGTT

>HuN_SY

TGGCTCATTTTCATTATGTTATGTCTTTGGGTTCTTATATTAGGGTTATTATATTTTTTGTTTGGTGATGGCCTGTTATCACAGGGGTTAGCCTGAATAAGTATTTGTTACAGTGTCATTGTATAGTATCAAATGTGGGTTTTAATTTGTGTTTTTTCCCTATGCATTATTTTGGTATTTGTGGTTTACCTCGGCGTGTTTGTGTGTATGAGTCAGGGTACGCTTGAGTTAATATGCTTTGTTCAATAGGTTCTTTTGTTTCTGCCTTTAGTGGTTGCTTTTTTATTTTTATTTTATGGGAGTCTTTAGCTAAAAAGAATGTTGTTATAGGTTATTATGGCAGTTCTTCAACTTTGCTTAATCTGTGTTGATCGCCGGTGCCTTATCACAGTAATTTTTTTGTGCGCGGATTATTTGTTGATTATTCTGTATTGGCTTTTTAGTTTATTGTTTAGAATATTGATTTTGTAAATCAGGGGTAGGGTGTTTTGACCTTTAAGCCTGTTTGGTGGAACTTTTAATTGGGTTGATTGGTTTTAATTGCCTTTTGCATCATGCTATATGGATGTGGTTAGTATATGGGCTATAGACCGAAAAGTTTGGATCTAGTATCAAGTTGGTT

>HuN_YY

TGGCTCATTTTCATTATGTTATGTCTTTGGGTTCTTATATTAGGGTTATTATATTTTTTGTTTGGTGATGGCCTGTTATTACAGGGGTTAGCCTGAATAAGTATTTGTTACAGTGTCATTGTATAGTATCAAATGTGGGTTTTAATTTGTGTTTTTTCCCTATGCATTATTTTGGTATTTGTGGTTTACCTCGGCGTGTTTGTGTGTATGAGTCAGGGTACGCTTGAGTTAATATGCTTTGTTCAATAGGTTCTTTTGTTTCTGCCTTTAGTGGTTGCTTTTTTATTTTTATTTTATGGGAGTCTTTAGCTAAAAAGAATGTTGTTATAGGTTATTATGGTAATTCTTCAACTTTGCTTAATCTGTGTTGATCGCCAGTGCCTTATCACAGTAATTTTTTTGTGCGCGGATTATTTGTTGATTATTCTGTATTGGCTTTTTAGTTTATTGTTTAGAATATTGATTTTGTAAATCAGGGGTAGGGTGTTTTGACCTTTAAGCCTGTTTGGTGGAACTTTTAATTGGGTTGATTGGTTTTAATTGCCTTTTGCATCATGCTATATGGATGTGGTTAGTATATGGGCTATAGACCGAAAAGTTTGGATCTAGTATCAAGTTGGTT

>HuN_HY

TGGCTCATTTTCATTATGTTATGTCTTTGGGTTCTTATATTAGGGTTATTATATTTTTTGTTTGGTGATGGCCTGTTATCACAGGGGTTAGCTTGAATAAGTATTTGTTACAGTGTCATTGTATAGTATCAAATGTGGGCTTTAATTTGTGTTTTTTTCCTATGCATTATTTTGGTATTTGTGGTTTACCTCGGCGTGTTTGTGTGTATGAGTCAGGGTACGCTTGAGTTAATATGCTTTGTTCAATAGGTTCTTTTGTTTCTGCCTTTAGTGGTTGCTTTTTTATTTTTATTTTATGGGAGTCTTTAGCTAAAAAGAATGTTGTTATAGGTTATTATGGTAGTTCTTCAACTTTGCTTAATTTGTGTTGATCGCCAGTGCCTTACCACAGTAATTTTTTTGTGCGTGGATTATTTGTTGATTATTCTGTATTGGCTTTTTAGTTTATTGTTTAGAATATTGATTTTGTAAATCAGGGGTAGGGTGTTTTGACCTTTAAGCCTGTTTGGTGGAACTTTTAATTGGGTTGATTGGTTTTAATTGCCTTTTGCATCATGCTATATGGATGTGGTTAGTATATGGGCTATACACCGAAAAGTTTGGATCTAGTATCAAGTTGGTT

>HuN_XT

TGGCTCATTTTCATTATGTTATGTCTTTGGGTTCTTATATTAGGGTTATTATATTTTTTGTTTGGTGATGGCCTGTTATCACAGGGGTTAGCCTGAATAAGTATTTGTTACAGTGTCATTGTATAGTATCAAATGTGGGTTTTAATTTGTGTTTTTTCCCTATGCATTATTTTGGTATTTGTGGTTTACCTCGGCGTGTTTGTGTGTATGAGTCAGGGTACGCTTGAGTTAATATGCTTTGTTCAATAGGTTCTTTTGTTTCTGCCTTTAGTGGTTGCTTTTTTATTTTTATTTTATGGGAGTCTTTAGCTAAAAAGAATGTTGTTATAGGTTATTATGGCAGTTCTTCAACTTTGCTTAATCTGTGTTGATCGCCGGTGCCTTATCACAGTAATTTTTTTGTGCGCGGATTATTTGTTGATTATTCTGTATTGGCTTTTTAGTTTATTGTTTAGAATATTGATTTTGTAAATCAGGGGTAGGGTGTTTTGACCTTTAAGCCTGTTTGGTGGAACTTTTAATTGGGTTGATTGGTTTTAATTGCCTTTTGCATCATGCTATATGGATGTGGTTAGTATATGGGCTATAGACCGAAAAGTTTGGATCTAGTATCAAGTTGGTT

>HuN_XX

TGGCTCATTTTCATTATGTTATGTCTTTGGGTTCTTATATTAGGATTATTATATTTTTTGTTTGGTGATGGCCTGTTATCACAGGGGTTAGCCTGAATAAGTATTTGTTACAGTGTCATTGTATAGTATCAAATGTGGGTTTTAATTTGTGTTTTTTCCCTATGCATTATTTTGGTATTTGTGGTTTACCTCGGCGTGTTTGTGTGTATGAGTCAGGGTACGCTTGAGTTAATATGCTTTGTTCAATAGGTTCTTTTGTTTCTGCCTTTAGTGGTTGCTTTTTTATTTTTATTTTATGGGAGTCTTTAGCTAAAAAGAATGTTGTTATAGGTTATTATGGTAGTTCTTCAACTTTGCTTAATCTGTGTTGGTCGCCAGTGCCTTATCACAGTAATTTTTTTGTGCGCGGATTATTTGTTGATTATTCTGTATTGGCTTTTTAGTTTATTGTTTAGAATATTGATTTTGTAAATCAGGGGTAGGGTGTTTTGACCTTTAAGCCTGTTTGGTGGAACTTTTAATTGGGTTGATTGGTTTTAATTGCCTTTTGCATCATGCTATATGGATGTGGTTAGTATATGGGCTATAGACCGAAAAGTTTGGATCTAGTATCAAGTTGGTT

>HuN_CS

TGGCTCATTTTCATTATGTTATGTCTTTGGGTTCTTATATTAGGATTATTATATTTTTTGTTTGGTGATGGCCTGTTATCACAGGGGTTAGCTTGAATAAGTATTTGTTACAGTGTCATTGTATAGTATCAAATGTGGGCTTTAATTTGTGTTTTTTTCCTATGCATTATTTTGGTATTTGTGGTTTACCTCGGCGTGTTTGTGTGTATGAGTCAGGGTACGCTTGAGTTAATATGCTTTGTTCAATAGGTTCTTTTGTTTCTGCCTTTAGTGGTTGCTTTTTTATTTTTATTTTATGGGAGTCTTTAGCTAAAAAGAATGTTGTTATAGGTTATTATGGTAGTTCTTCAACTTTGCTTAATTTGTGTTGATCGCCAGTGCCTTACCACAGTAATTTTTTTGTGCGTGGATTATTTGTTGATTATTCTGTATTGGCTTTTTAGTTTATTGTTTAGAATATTGATTTTGTAAATCAGGGGTAGGGTGTTTTGACCTTTAAGCCTGTTTGGTGGAACTTTTAATTGGGTTGATTGGTTTTAATTGCCTTTTGCATCATGCTATATGGATGTGGTTAGTATATGGGCTATACACCGAAAAGTTTGGATCTAGTATCAAGTTGGTT

>GD_DG

TGGCTCATTTTCATTATGTTATGTCTTTGGGTTCTTATATTAGGGTTATTATATTTTTTGTTTGGTGATGGCCTGTTATCACAGGGGTTAGCTTGAATAAGTATTTGTTACAGTGTCATTGTATAGTATCAAATGTGGGCTTTAATTTGTGTTTTTTTCCTATGCATTATTTTGGTATTTGTGGTTTACCTCGGCGTGTTTGTGTGTATGAGTCAGGGTACGCTTGAGTTAATATGCTTTGTTCAATAGGTTCTTTTGTTTCTGCCTTTAGTGGTTGCTTTTTTATTTTTATTTTATGGGAGTCTTTAGCTAAAAAGAATGTTGTTATAGGTTATTATGGTAGTTCTTCAACTTTGCTTAATTTGTGTTGATCGCCAGTGCCTTACCACAGTAATTTTTTTGTGCGTGGATTATTTGTTGATTATTCTGTATTGGCTTTTTAGTTTATTGTTTAGAATATTGATTTTGTAAATCAGGGGTAGGGTGTTTTGACCTTTAAGCCTGTTTGGTGGAACTTTTAATTGGGTTGATTGGTTTTAATTGCCTTTTGCATCATGCTATATGGATGTGGTTAGTATATGGGCTATACACCGAAAAGTTTGGATCTAGTATCAAGTTGGTT

>GD_GZ

TGGCTCATTTTCATTATGTTATGTCTTTGGGTTCTTATATTAGGGTTATTATATTTTTTGTTTGGTGATGGCCTGTTATCACAGGGGTTAGCTTGAATAAGTATTTGTTACAGTGTCATTGTATAGTATCAAATGTGGGCTTTAATTTGTGTTTTTTTCCTATGCATTATTTTGGTATTTGTGGTTTACCTCGGCGTGTTTGTGTGTATGAGTCAGGGTACGCTTGAGTTAATATGCTTTGTTCAATAGGTTCTTTTGTTTCTGCCTTTAGTGGTTGCTTTTTTATTTTTATTTTATGGGAGTCTTTAGCTAAAAAGAATGTTGTTATAGGTTATTATGGTAGTTCTTCAACTTTGCTTAATTTGTGTTGATCGCCAGTGCCTTACCACAGTAATTTTTTTGTGCGTGGATTATTTGTTGATTATTCTGTATTGGCTTTTTAGTTTATTGTTTAGAATATTGATTTTGTAAATCAGGGGTAGGGTGTTTTGACCTTTAAGCCTGTTTGGTGGAACTTTTAATTGGGTTGATTGGTTTTAATTGCCTTTTGCATCATGCTATATGGATGTGGTTAGTATATGGGCTATACACCGAAAAGTTTGGATCTAGTATCAAGTTGGTT

>GD_JM

TGGCTCATTTTCATTATGTTATGTCTTTGGGTTCTTATATTAGGATTATTATATTTTTTGTTTGGTGATGGCCTGTTATCACAGGGGTTAGCCTGAATAAGTATTTGTTACAGTGTCATTGTATAGTATCAAATGTGGGTTTTAATTTGTGTTTTTTCCCTATGCATTATTTTGGTATTTGTGGTTTACCTCGGCGTGTTTGTGTGTATGAGTCAGGGTACGCTTGAGTTAATATGCTTTGTTCAATAGGTTCTTTTGTTTCTGCCTTTAGTGGTTGCTTTTTTATTTTTATTTTATGGGAGTCTTTAGCTAAAAAGAATGTTGTTATAGGTTATTATGGTAGTTCTTCAACTTTGCTTAATCTGTGTTGGTCGCCAGTGCCTTATCACAGTAATTTTTTTGTGCGCGGATTATTTGTTGATTATTCTGTATTGGCTTTTTAGTTTATTGTTTAGAATATTGATTTTGTAAATCAGGGGTAGGGTGTTTTGACCTTTAAGCCTGTTTGGTGGAACTTTTAATTGGGTTGATTGGTTTTAATTGCCTTTTGCATCATGCTATATGGATGTGGTTAGTATATGGGCTATAGACCGAAAAGTTTGGATCTAGTATCAAGTTGGTT

>GD_FS

TGGCTCATTTTCATTATGTTATGTCTTTGGGTTCTTATATTAGGATTATTATATTTTTTGTTTGGTGATGGCCTGTTATCACAGGGGTTAGCCTGAATAAGTATTTGTTACAGTGTCATTGTATAGTATCAAATGTGGGTTTTAATTTGTGTTTTTTCCCTATGCATTATTTTGGTATTTGTGGTTTACCTCGGCGTGTTTGTGTGTATGAGTCAGGGTACGCTTGAGTTAATATGCTTTGTTCAATAGGTTCTTTTGTTTCTGCCTTTAGTGGTTGCTTTTTTATTTTTATTTTATGGGAGTCTTTAGCTAAAAAGAATGTTGTTATAGGTTATTATGGTAGTTCTTCAACTTTGCTTAATCTGTGTTGGTCGCCAGTGCCTTATCACAGTAATTTTTTTGTGCGCGGATTATTTGTTGATTATTCTGTATTGGCTTTTTAGTTTATTGTTTAGAATATTGATTTTGTAAATCAGGGGTAGGGTGTTTTGACCTTTAAGCCTGTTTGGTGGAACTTTTAATTGGGTTGATTGGTTTTAATTGCCTTTTGCATCATGCTATATGGATGTGGTTAGTATATGGGCTATAGACCGAAAAGTTTGGATCTAGTATCAAGTTGGTT

>GX_WZ

TGGCTCATTTTCATTATGTTATGTCTTTGGGTTCTTATATTAGGATTATTATATTTTTTGTTTGGTGATGGCCTGTTATCACAGGGGTTAGCCTGAATAAGTATTTGTTACAGTGTCATTGTATAGTATCAAATGTGGGTTTTAATTTGTGTTTTTTCCCTATGCATTATTTTGGTATTTGTGGTTTACCTCGGCGTGTTTGTGTGTATGAGTCAGGGTACGCTTGAGTTAATATGCTTTGTTCAATAGGTTCTTTTGTTTCTGCCTTTAGTGGTTGCTTTTTTATTTTTATTTTATGGGAGTCTTTAGCTAAAAAGAATGTTGTTATAGGTTATTATGGTAGTTCTTCAACTTTGCTTAATCTGTGTTGGTCGCCAGTGCCTTATCACAGTAATTTTTTTGTGCGCGGATTATTTGTTGATTATTCTGTATTGGCTTTTTAGTTTATTGTTTAGAATATTGATTTTGTAAATCAGGGGTAGGGTGTTTTGACCTTTAAGCCTGTTTGGTGGAACTTTTAATTGGGTTGATTGGTTTTAATTGCCTTTTGCATCATGCTATATGGATGTGGTTAGTATATGGGCTATAGACCGAAAAGTTTGGATCTAGTATCAAGTTGGTT

>GX_YL

TGGCTCATTTTCATTATGTTATGTCTTTGGGTTCTTATATTAGGATTATTATATTTTTTGTTTGGTGATGGCCTGTTATCACAGGGGTTAGCCTGAATAAGTATTTGTTACAGTGTCATTGTATAGTATCAAATGTGGGTTTTAATTTGTGTTTTTTCCCTATGCATTATTTTGGTATTTGTGGTTTACCTCGGCGTGTTTGTGTGTATGAGTCAGGGTACGCTTGAGTTAATATGCTTTGTTCAATAGGTTCTTTTGTTTCTGCCTTTAGTGGTTGCTTTTTTATTTTTATTTTATGGGAGTCTTTAGCTAAAAAGAATGTTGTTATAGGTTATTATGGTAGTTCTTCAACTTTGCTTAATCTGTGTTGGTCGCCAGTGCCTTATCACAGTAATTTTTTTGTGCGCGGATTATTTGTTGATTATTCTGTATTGGCTTTTTAGTTTATTGTTTAGAATATTGATTTTGTAAATCAGGGGTAGGGTGTTTTGACCTTTAAGCCTGTTTGGTGGAACTTTTAATTGGGTTGATTGGTTTTAATTGCCTTTTGCATCATGCTATATGGATGTGGTTAGTATATGGGCTATAGACCGAAAAGTTTGGATCTAGTATCAAGTTGGTT

>GX_NN

TGGCTCATTTTCATTATGTTATGTCTTTGGGTTCTTATATTAGGATTATTATATTTTTTGTTTGGTGATGGCCTGTTATCACAGGGGTTAGCCTGAATAAGTATTTGTTACAGTGTCATTGTATAGTATCAAATGTGGGTTTTAATTTGTGTTTTTTCCCTATGCATTATTTTGGTATTTGTGGTTTACCTCGGCGTGTTTGTGTGTATGAGTCAGGGTACGCTTGAGTTAATATGCTTTGTTCAATAGGTTCTTTTGTTTCTGCCTTTAGTGGTTGCTTTTTTATTTTTATTTTATGGGAGTCTTTAGCTAAAAAGAATGTTGTTATAGGTTATTATGGTAGTTCTTCAACTTTGCTTAATCTGTGTTGGTCGCCAGTGCCTTATCACAGTAATTTTTTTGTGCGCGGATTATTTGTTGATTATTCTGTATTGGCTTTTTAGTTTATTGTTTAGAATATTGATTTTGTAAATCAGGGGTAGGGTGTTTTGACCTTTAAGCCTGTTTGGTGGAACTTTTAATTGGGTTGATTGGTTTTAATTGCCTTTTGCATCATGCTATATGGATGTGGTTAGTATATGGGCTATAGACCGAAAAGTTTGGATCTAGTATCAAGTTGGTT

>GX_GL

TGGCTCATTTTCATTATGTTATGTCTTTGGGTTCTTATATTAGGATTATTATATTTTTTGTTTGGTGATGGCCTGTTATCACAGGGGTTAGCCTGAATAAGTATTTGTTACAGTGTCATTGTATAGTATCAAATGTGGGTTTTAATTTGTGTTTTTTCCCTATGCATTATTTTGGTATTTGTGGTTTACCTCGGCGTGTTTGTGTGTATGAGTCAGGGTACGCTTGAGTTAATATGCTTTGTTCAATAGGTTCTTTTGTTTCTGCCTTTAGTGGTTGCTTTTTTATTTTTATTTTATGGGAGTCTTTAGCTAAAAAGAATGTTGTTATAGGTTATTATGGTAGTTCTTCAACTTTGCTTAATCTGTGTTGGTCGCCAGTGCCTTATCACAGTAATTTTTTTGTGCGCGGATTATTTGTTGATTATTCTGTATTGGCTTTTTAGTTTATTGTTTAGAATATTGATTTTGTAAATCAGGGGTAGGGTGTTTTGACCTTTAAGCCTGTTTGGTGGAACTTTTAATTGGGTTGATTGGTTTTAATTGCCTTTTGCATCATGCTATATGGATGTGGTTAGTATATGGGCTATAGACCGAAAAGTTTGGATCTAGTATCAAGTTGGTT

>HaN_HK

TGGCTCATTTTCATTATGTTATGTCTTTGGGTTCTTATATTAGGGTTATTATATTTTTTGTTTGGTGATGGCCTGTTATTACAGGGGTTAGCCTGAATAAGTATTTGTTACAGTGTCATTGTATAGTATCAAATGTGGGTTTTAATTTGTGTTTTTTCCCTATGCATTATTTTGGTATTTGTGGTTTACCTCGGCGTGTTTGTGTGTATGAGTCAGGGTACGCTTGAGTTAATATGCTTTGTTCAATAGGTTCTTTTGTTTCTGCCTTTAGTGGTTGCTTTTTTATTTTTATTTTATGGGAGTCTTTAGCTAAAAAGAATGTTGTTATAGGTTATTATGGTAATTCTTCAACTTTGCTTAATCTGTGTTGATCGCCAGTGCCTTATCACAGTAATTTTTTTGTGCGCGGATTATTTGTTGATTATTCTGTATTGGCTTTTTAGTTTATTGTTTAGAATATTGATTTTGTAAATCAGGGGTAGGGTGTTTTGACCTTTAAGCCTGTTTGGTGGAACTTTTAATTGGGTTGATTGGTTTTAATTGCCTTTTGCATCATGCTATATGGATGTGGTTAGTATATGGGCTATAGACCGAAAAGTTTGGATCTAGTATCAAGTTGGTT

>HaN_WZS

TGGCTCATTTTCATTATGTTATGTCTTTGGGTTCTTATATTAGGGTTATTATATTTTTTGTTTGGTGATGGCCTGTTATTACAGGGGTTAGCCTGAATAAGTATTTGTTACAGTGTCATTGTATAGTATCAAATGTGGGTTTTAATTTGTGTTTTTTCCCTATGCATTATTTTGGTATTTGTGGTTTACCTCGGCGTGTTTGTGTGTATGAGTCAGGGTACGCTTGAGTTAATATGCTTTGTTCAATAGGTTCTTTTGTTTCTGCCTTTAGTGGTTGCTTTTTTATTTTTATTTTATGGGAGTCTTTAGCTAAAAAGAATGTTGTTATAGGTTATTATGGTAATTCTTCAACTTTGCTTAATCTGTGTTGATCGCCAGTGCCTTATCACAGTAATTTTTTTGTGCGCGGATTATTTGTTGATTATTCTGTATTGGCTTTTTAGTTTATTGTTTAGAATATTGATTTTGTAAATCAGGGGTAGGGTGTTTTGACCTTTAAGCCTGTTTGGTGGAACTTTTAATTGGGTTGATTGGTTTTAATTGCCTTTTGCATCATGCTATATGGATGTGGTTAGTATATGGGCTATAGACCGAAAAGTTTGGATCTAGTATCAAGTTGGTT

>SC_DZ

TGGCTCATTTTCATTATGTTATGTCTTTGGGTTCTTATATTAGGATTATTATATTTTTTGTTTGGTGATGGCCTGTTATCACAGGGGTTAGCCTGAATAAGTATTTGTTACAGTGTCATTGTATAGTATCAAATGTGGGTTTTAATTTGTGTTTTTTCCCTATGCATTATTTTGGTATTTGTGGTTTACCTCGGCGTGTTTGTGTGTATGAGTCAGGGTACGCTTGAGTTAATATGCTTTGTTCAATAGGTTCTTTTGTTTCTGCCTTTAGTGGTTGCTTTTTTATTTTTATTTTATGGGAGTCTTTAGCTAAAAAGAATGTTGTTATAGGTTATTATGGTAGTTCTTCAACTTTGCTTAATCTGTGTTGGTCGCCAGTGCCTTACCACAGTAATTTTTTTGTGCGCGGATTATTTGTTGATTATTCTGTATTGGCTTTTTAGTTTATTGTTTAGAATATTGATTTTGTAAATCAGGGGTAGGGTGTTTTGACCTTTAAGCCTGTTTGGTGGAACTTTTAATTGGGTTGATTGGTTTTAATTGCCTTTTGCATCATGCTATATGGATGTGGTTAGTATATGGGCTATACACCGAAAAGTTTGGATCTAGTATCAAGTTGGTT

>SC_NC

TGGCTCATTTTCATTATGTTATGTCTTTGGGTTCTTATATTAGGATTATTATATTTTTTGTTTGGTGATGGCCTGTTATCACAGGGGTTAGCCTGAATAAGTATTTGTTACAGTGTCATTGTATAGTATCAAATGTGGGTTTTAATTTGTGTTTTTTCCCTATGCATTATTTTGGTATTTGTGGTTTACCTCGGCGTGTTTGTGTGTATGAGTCAGGGTACGCTTGAGTTAATATGCTTTGTTCAATAGGTTCTTCTGTTTCTGCCTTTAGTGGTTGCTTTTTTATTTTTATTTTATGGGAGTCTTTAGCTAAAAAGAATGTTGTTATAGGTTATTATGGTAGTTCTTCAACTTTGCTTAATCTGTGTTGGTCGCCAGTGCCTTACCACAGTAATTTTTTTGTGCGCGGATTATTTGTTGATTATTCTGTATTGGCTTTTTAGTTTATTGTTTAGAATATTGATTTTGTAAATCAGGGGTAGGGTGTTTTGACCTTTAAGCCTGTTTGGTGGAACTTTTAATTGGGTTGATTGGTTTTAATTGCCTTTTGCATCATGCTATATGGATGTGGTTAGTATATGGGCTATACACCGAAAAGTTTGGATCTAGTATCAAGTTGGTT

>SC_GA

TGGCTCATTTTCATTATGTTATGTCTTTGGGTTCTTATATTAGGATTATTATATTTTTTGTTTGGTGATGGCCTGTTATCACAGGGGTTAGCCTGAATAAGTATTTGTTACAGTGTCATTGTATAGTATCAAATGTGGGTTTTAATTTGTGTTTTTTCCCTATGCATTATTTTGGTATTTGTGGTTTACCTCGGCGTGTTTGTGTGTATGAGTCAGGGTACGCTTGAGTTAATATGCTTTGTTCAATAGGTTCTTTTGTTTCTGCCTTTAGTGGTTGCTTTTTTATTTTTATTTTATGGGAGTCTTTAGCTAAAAAGAATGTTGTTATAGGTTATTATGGTAGTTCTTCAACTTTGCTTAATCTGTGTTGGTCGCCAGTGCCTTACCACAGTAATTTTTTTGTGCGCGGATTATTTGTTGATTATTCTGTATTGGCTTTTTAGTTTATTGTTTAGAATATTGATTTTGTAAATCAGGGGTAGGGTGTTTTGACCTTTAAGCCTGTTTGGTGGAACTTTTAATTGGGTTGATTGGTTTTAATTGCCTTTTGCATCATGCTATATGGATGTGGTTAGTATATGGGCTATACACCGAAAAGTTTGGATCTAGTATCAAGTTGGTT

>SC_LZ

TGGCTCATTTTCATTATGTTATGTCTTTGGGTTCTTATATTAGGGTTATTATATTTTTTGTTTGGTGATGGCCTGTTATCACAGGGGTTAGCTTGAATAAGTATTTGTTACAGTGTCATTGTATAGTATCAAATGTGGGCTTTAATTTGTGTTTTTTTCCTATGCATTATTTTGGTATTTGTGGTTTACCTCGGCGTGTTTGTGTGTATGAGTCAGGGTACGCTTGAGTTAATATGCTTTGTTCAATAGGTTCTTTTGTTTCTGCCTTTAGTGGTTGCTTTTTTATTTTTATTTTATGGGAGTCTTTAGCTAAAAAGAATGTTGTTATAGGTTATTATGGTAGTTCTTCAACTTTGCTTAATTTGTGTTGATCGCCAGTGCCTTACCACAGTAATTTTTTTGTGCGTGGATTATTTGTTGATTATTCTGTATTGGCTTTTTAGTTTATTGTTTAGAATATTGATTTTGTAAATCAGGGGTAGGGTGTTTTGACCTTTAAGCCTGTTTGGTGGAACTTTTAATTGGGTTGATTGGTTTTAATTGCCTTTTGCATCATGCTATATGGATGTGGTTAGTATATGGGCTATACACCGAAAAGTTTGGATCTAGTATCAAGTTGGTT

>SC_LSZ

TGGCTCATTTTCATTATGTTATGTCTTTGGGTTCTTATATTAGGGTTATTATATTTTTTGTTTGGTGATGGCCTGTTATCACAGGGGTTAGCTTGAATAAGTATTTGTTACAGTGTCATTGTATAGTATCAAATGTGGGCTTTAATTTGTGTTTTTTTCCTATGCATTATTTTGGTATTTGTGGTTTACCTCGGCGTGTTTGTGTGTATGAGTCAGGGTACGCTTGAGTTAATATGCTTTGTTCAATAGGTTCTTTTGTTTCTGCCTTTAGTGGTTGCTTTTTTATTTTTATTTTATGGGAGTCTTTAGCTAAAAAGAATGTTGTTATAGGTTATTATGGTAGTTCTTCAACTTTGCTTAATTTGTGTTGGTCGCCAGTGCCTTACCACAGTAATTTTTTTGTGCGTGGATTATTTGTTGATTATTCTGTATTGGCTTTTTAGTTTATTGTTTAGAATATTGATTTTGTAAATCAGGGGTAGGGTGTTTTGACCTTTAAGCCTGTTTGGTGGAACTTTTAATTGGGTTGATTGGTTTTAATTGCCTTTTGCATCATGCTATATGGATGTGGTTAGTATATGGGCTATAGACCGAAAAGTTTGGATCTAGTATCAAGTTGGTT

>SC_LS

TGGCTCATTTTCATTATGTTATGTCTTTGGGTTCTTATATTAGGGTTATTATATTTTTTGTTTGGTGATGGCCTGTTATCACAGGGGTTAGCTTGAATAAGTATTTGTTACAGTGTCATTGTATAGTATCAAATGTGGGCTTTAATTTGTGTTTTTTTCCTATGCATTATTTTGGTATTTGTGGTTTACCTCGGCGTGTTTGTGTGTATGAGTCAGGGTACGCTTGAGTTAATATGCTTTGTTCAATAGGTTCTTTTGTTTCTGCCTTTAGTGGTTGCTTTTTTATTTTTATTTTATGGGAGTCTTTAGCTAAAAAGAATGTTGTTATAGGTTATTATGGTAGTTCTTCAACTTTGCTTAATTTGTGTTGGTCGCCAGTGCCTTACCACAGTAATTTTTTTGTGCGTGGATTATTTGTTGATTATTCTGTATTGGCTTTTTAGTTTATTGTTTAGAATATTGATTTTGTAAATCAGGGGTAGGGTGTTTTGACCTTTAAGCCTGTTTGGTGGAACTTTTAATTGGGTTGATTGGTTTTAATTGCCTTTTGCATCATGCTATATGGATGTGGTTAGTATATGGGCTATACACCGAAAAGTTTGGATCTAGTATCAAGTTGGTT

>SC_ZG

TGGCTCATTTTCATTATGTTATGTCTTTGGGTTCTTATATTAGGGTTATTATATTTTTTGTTTGGTGATGGCCTGTTATCACAGGGGTTAGCTTGAATAAGTATTTGTTACAGTGTCATTGTATAGTATCAAATGTGGGCTTTAATTTGTGTTTTTTTCCTATGCATTATTTTGGTATTTGTGGTTTACCTCGGCGTGTTTGTGTGTATGAGTCAGGGTACGCTTGAGTTAATATGCTTTGTTCAATAGGTTCTTTTGTTTCTGCCTTTAGTGGTTGCTTTTTTATTTTTATTTTATGGGAGTCTTTAGCTAAAAAGAATGTTGTTATAGGTTATTATGGTAGTTCTTCAACTTTGCTTAATTTGTGTTGATCGCCAGTGCCTTACCACAGTAATTTTTTTGTGCGTGGATTATTTGTTGATTATTCTGTATTGGCTTTTTAGTTTATTGTTTAGAATATTGATTTTGTAAATCAGGGGTAGGGTGTTTTGACCTTTAAGCCTGTTTGGTGGAACTTTTAATTGGGTTGATTGGTTTTAATTGCCTTTTGCATCATGCTATATGGATGTGGTTAGTATATGGGCTATACACCGAAAAGTTTGGATCTAGTATCAAGTTGGTT

>YN_KM

TGGCTCATTTTCATTATGTTATGTCTTTGGGTTCTTATATTAGGATTATTATATTTTTTGTTTGGTGATGGCCTGTTATTACAGGGGTTAGCCTGAATAAGTATTTGTTACAGTGTCATTGTATAGTATCAAATGTGGGTTTTAATTTGTGTTTTTTCCCTATGCATTATTTTGGTATTTGTGGTTTACCTCGGCGTGTTTGTGTGTATGAGTCAGGGTACGCTTGAGTTAATATGCTTTGTTCAATAGGTTCTTTTGTTTCTGCCTTTAGTGGTTGCTTTTTTATTTTTATTTTATGGGAGTCTTTAGCTAAAAAGAATGTTGTTATAGGTTATTATGGTAGTTCTTCAACTTTGCTTAATCTGTGTTGATCGCCAGTGCCTTATCACAGTAATTTTTTTGTGCGCGGATTATTTGTTGATTATTCTGTATTGGCTTTTTAGTTTATTGTTTAGAATATTGATTTTGTAAATCAGGGGTAGGGTGTTTTGACCTTTAAGCCTGTTTGGTGGAACTTTTAATTGGGTTGATTGGTTTTAATTGCCTTTTGCATCATGCTATATGGATGTGGTTAGTATATGGGCTATAGACCGAAAAGTTTGGATCTAGTATCAAGTTGGTT

>YN_BS

TGGCTCATTTTCATTATGTTATGTCTTTGGGTTCTTATATTAGGATTATTATATTTTTTGTTTGGTGATGGCCTGTTATTACAGGGGTTAGCCTGAATAAGTATTTGTTACAGTGTCATTGTATAGTATCAAATGTGGGTTTTAATTTGTGTTTTTTCCCTATGCATTATTTTGGTATTTGTGGTTTACCTCGGCGTGTTTGTGTGTATGAGTCAGGGTACGCTTGAGTTAATATGCTTTGTTCAATAGGTTCTTTTGTTTCTGCCTTTAGTGGTTGCTTTTTTATTTTTATTTTATGGGAGTCTTTAGCTAAAAAGAATGTTGTTATAGGTTATTATGGTAGTTCTTCAACTTTGCTTAATCTGTGTTGATCGCCAGTGCCTTATCACAGTAATTTTTTTGTGCGCGGATTATCTGTTGATTATTCTGTATTGGCTTTTTAGTTTATTGTTTAGAATATTGATTTTGTAAATCAGGGGTAGGGTGTTTTGACCTTTAAGCCTGTTTGGTGGAACTTTTAATTGGGTTGATTGGTTTTAATTGCCTTTTGCATCATGCTATATGGATGTGGTTAGTATATGGGCTATAGACCGAAAAGTTTGGATCTAGTATCAAGTTGGTT

>YN_DHZ

TGGCTCATTTTCATTATGTTATGTCTTTGGGTTCTTATATTAGGATTATTATATTTTTTGTTTGGTGATGGCCTGTTATTACAGGGGTTAGCTTGAATAAGTATTTGTTACAGTGTCATTGTATAGTATCAAATGTGGGTTTTAATTTGTGTTTTTTCCCTATGCATTATTTTGGTATTTGTGGTTTACCTCGGCGTGTTTGTGTGTATGAGTCAGGGTACGCTTGAGTTAATATGCTTTGTTCAATAGGTTCTTTTGTTTCTGCCTTTAGTGGTTGCTTTTTTATTTTTATTTTATGGGAGTCTTTAGCTAAAAAGAATGTTGTTATAGGTTATTATGGTAGTTCTTCAACTTTGCTTAATCTGTGTTGATCGCCAGTGCCTTATCACAGTAATTTTTTTGTGCGCGGATTATTTGTTGATTATTCTGTATTGGCTTTTTAGTTTATTGTTTAGAATATTGATTTTGTAAATCAGGGGTAGGGTGTTTTGACCTTTAAGCCTGTTTGGTGGAACTTTTAATTGGGTTGATTGGTTTTAATTGCCTTTTGCATCATGCTATATGGATGTGGTTAGTATA-------------------------------------------

>YN_WS

TGGCTCATTTTCATTATGTTATGTCTTTGGGTTCTTATATTAGGATTATTATATTTTTTGTTTGGTGATGGCCTGTTATTACAGGGGTTAGCTTGAATAAGTATTTGTTACAGTGTCATTGTATAGTATCAAATGTGGGTTTTAATTTGTGTTTTTTCCCTATGCATTATTTTGGTATTTGTGGTTTACCTCGGCGTGTTTGTGTGTATGAGTCAGGGTACGCTTGAGTTAATATGCTTTGTTCAATAGGTTCTTTTGTTTCTGCCTTTAGTGGTTGCTTTTTTATTTTTATTTTATGGGAGTCTTTAGCTAAAAAGAATGTTGTTATAGGTTATTATGGTAGTTCTTCAACTTTGCTTAATCTGTGTTGATCGCCAGTGCCTTATCACAGTAATTTTTTTGTGCGCGGATTATTTGTTGATTATTCTGTATTGGCTTTTTAGTTTATTGTTTAGAATATTGATTTTGTAAATCAGGGGTAGGGTGTTTTGACCTTTAAGCCTGTTTGGTGGAACTTTTAATTGGGTTGATTGGTTTTAATTGCCTTTTGCATCATGCTATATGGATGTGGTTAGTATATGGGCTATAGACCGAAAAGTTTGGATCTAGTATCAAGTTGGTT

>YN_HH

TGGCTCATTTTCATTATGTTATGTCTTTGGGTTCTTATATTAGGATTATTATATTTTTTGTTTGGTGATGGCCTGTTATTACAGGGGTTAGCTTGAATAAGTATTTGTTACAGTGTCATTGTATAGTATCAAATGTGGGTTTTAATTTGTGTTTTTTCCCTATGCATTATTTTGGTATTTGTGGTTTACCTCGGCGTGTTTGTGTGTATGAGTCAGGGTACGCTTGAGTTAATATGCTTTGTTCAATAGGTTCTTTTGTTTCTGCCTTTAGTGGTTGCTTTTTTATTTTTATTTTATGGGAGTCTTTAGCTAAAAAGAATGTTGTTATAGGTTATTATGGTAGTTCTTCAACTTTGCTTAATCTGTGTTGATCGCCAGTGCCTTATCACAGTAATTTTTTTGTGCGCGGATTATTTGTTGATTATTCTGTATTGGCTTTTTAGTTTATTGTTTAGAATATTGATTTTGTAAATCAGGGGTAGGGTGTTTTGACCTTTAAGCCTGTTTGGTGGAACTTTTAATTGGGTTGATTGGTTTTAATTGCCTTTTGCATCATGCTATATGGATGTGGTTAGTATATGGGCTATAGACCGAAAAGTTTGGATCTAGTATCAAGTTGGTT

>GZ_ZY

TGGCTCATTTTCATTATGTTATGTCTTTGGGTTCTTATATTAGGATTATTATATTTTTTGTTTGGTGATGGCCTGTTATCACAGGGGTTAGCCTGAATAAGTATTTGTTACAGTGTCATTGTATAGTATCAAATGTGGGTTTTAATTTGTGTTTTTTCCCTATGCATTATTTTGGTATTTGTGGTTTACCTCGGCGTGTTTGTGTGTATGAGTCAGGGTACGCTTGAGTTAATATGCTTTGTTCAATAGGTTCTTTTGTTTCTGCCTTTAGTGGTTGCTTTTTTATTTTTATTTTATGGGAGTCTTTAGCTAAAAAGAATGTTGTTATAGGTTATTATGGTAGTTCTTCAACTTTGCTTAATCTGTGTTGGTCGCCAGTGCCTTATCACAGTAATTTTTTTGTGCGCGGATTATTTGTTGATTATTCTGTATTGGCTTTTTAGTTTATTGTTTAGAATATTGATTTTGTAAATCAGGGGTAGGGTGTTTTGACCTTTAAGCCTGTTTGGTGGAACTTTTAATTGGGTTGATTGGTTTTAATTGCCTTTTGCATCATGCTATATGGATGTGGTTAGTATATGGGCTATAGACCGAAAAGTTTGGATCTAGTATCAAGTTGGTT

>GZ_GY

TGGCTCATTTTCATTATGTTATGTCTTTGGGTTCTTATATTAGGATTATTATATTTTTTGTTTGGTGATGGCCTGTTATCACAGGGGTTAGCCTGAATAAGTATTTGTTACAGTGTCATTGTATAGTATCAAATGTGGGTTTTAATTTGTGTTTTTTCCCTATGCATTATTTTGGTATTTGTGGTTTACCTCGGCGTGTTTGTGTGTATGAGTCAGGGTACGCTTGAGTTAATATGCTTTGTTCAATAGGTTCTTTTGTTTCTGCCTTTAGTGGTTGCTTTTTTATTTTTATTTTATGGGAGTCTTTAGCTAAAAAGAATGTTGTTATAGGTTATTATGGTAGTTCTTCAACTTTGCTTAATCTGTGTTGGTCGCCAGTGCCTTATCACAGTAATTTTTTTGTGCGCGGATTATTTGTTGATTATTCTGTATTGGCTTTTTAGTTTATTGTTTAGAATATTGATTTTGTAAATCAGGGGTAGGGTGTTTTGACCTTTAAGCCTGTTTGGTGGAACTTTTAATTGGGTTGATTGGTTTTAATTGCCTTTTGCATCATGCTATATGGATGTGGTTAGTATATGGGCTATAGACCGAAAAGTTTGGATCTAGTATCAAGTTGGTT

>GZ_KL

TGGCTCATTTTCATTATGTTATGTCTTTGGGTTCTTATATTAGGGTTATTATATTTTTTGTTTGGTGATGGCCTGTTATCACAGGGGTTAGCTTGAATAAGTATTTGTTACAGTGTCATTGTATAGTATCAAATGTGGGCTTTAATTTGTGTTTTTTTCCTATGCATTATTTTGGTATTTGTGGTTTACCTCGGCGTGTTTGTGTGTATGAGTCAGGGTACGCTTGAGTTAATATGCTTTGTTCAATAGGTTCTTTTGTTTCTGCCTTTAGTGGTTGCTTTTTTATTTTTATTTTATGGGAGTCTTTAGCTAAAAAGAATGTTGTTATAGGTTATTATGGTAGTTCTTCAACTTTGCTTAATTTGTGTTGATCGCCAGTGCCTTACCACAGTAATTTTTTTGTGCGTGGGTTATTTGTTGATTATTCTGTATTGGCTTTTTAGTTTATTGTTTAGAATATTGATTTTGTAAATCAGGGGTAGGGTGTTTTGACCTTTAAGCCTGTTTGGTGGAACTTTTAATTGGGTTGATTGGTTTTAATTGCCTTTTGCATCATGCTATATGGATGTGGTTAGTATATGGGCTATAGACCGAAAAGTTTGGATCTAGTATCAAGTTGGTT

>GZ_AS

TGGCTCATTTTCATTATGTTATGTCTTTGGGTTCTTATATTAGGGTTATTATATTTTTTGTTTGGTGATGGCCTGTTATCACAGGGGTTAGCTTGAATAAGTATTTGTTACAGTGTCATTGTATAGTATCAAATGTGGGCTTTAATTTGTGTTTTTTTCCTATGCATTATTTTGGTATTTGTGGTTTACCTCGGCGTGTTTGTGTGTATGAGTCAGGGTACGCTTGAGTTAATATGCTTTGTTCAATAGGTTCTTTTGTTTCTGCCTTTAGTGGTTGCTTTTTTATTTTTATTTTATGGGAGTCTTTAGCTAAAAAGAATGTTGTTATAGGTTATTATGGTAGTTCTTCAACTTTGCTTAATTTGTGTTGATCGCCAGTGCCTTACCACAGTAATTTTTTTGTGCGTGGGTTATTTGTTGATTATTCTGTATTGGCTTTTTAGTTTATTGTTTAGAATATTGATTTTGTAAATCAGGGGTAGGGTGTTTTGACCTTTAAGCCTGTTTGGTGGAACTTTTAATTGGGTTGATTGGTTTTAATTGCCTTTTGCATCATGCTATATGGATGTGGTTAGTATATGGGCTATAGACCGAAAAGTTTGGATCTAGTATCAAGTTGGTT

>CQ_LP

TGGCTCATTTTCATTATGTTATGTCTTTGGGTTCTTATATTAGGGTTATTATATTTTTTGTTTGGTGATGGCCTGTTATCACAGGGGTTAGCTTGAATAAGTATTTGTTACAGTGTCATTGTATAGTATCAAATGTGGGCTTTAATTTGTGTTTTTTTCCTATGCATTATTTTGGTATTTGTGGTTTACCTCGGCGTGTTTGTGTGTATGAGTCAGGGTACGCTTGAGTTAATATGCTTTGTTCAATAGGTTCTTTTGTTTCTGCCTTTAGTGGTTGCTTTTTTATTTTTATTTTATGGGAGTCTTTAGCTAAAAAGAATGTTGTTATAGGTTATTATGGTAGTTCTTCAACTTTGCTTAATTTGTGTTGATCGCCAGTGCCTTACCACAGTAATTTTTTTGTGCGTGGATTATTTGTTGATTATTCTGTATTGGCTTTTTAGTTTATTGTTTAGAATATTGATTTTGTAAATCAGGGGTAGGGTGTTTTGACCTTTAAGCCTGTTTGGTGGAACTTTTAATTGGGTTGATTGGTTTTAATTGCCTTTTGCATCATGCTATATGGATGTGGTTAGTATATGGGCTATACACCGAAAAGTTTGGATCTAGTATCAAGTTGGTT

>CQ_Z

TGGCTCATTTTCATTATGTTATGTCTTTGGGTTCTTATATTAGGGTTATTATATTTTTTGTTTGGTGATGGCCTGTTATCACAGGGGTTAGCTTGAATAAGTATTTGTTACAGTGTCATTGTATAGTATCAAATGTGGGCTTTAATTTGTGTTTTTTTCCTATGCATTATTTTGGTATTTGTGGTTTACCTCGGCGTGTTTGTGTGTATGAGTCAGGGTACGCTTGAGTTAATATGCTTTGTTCAATAGGTTCTTTTGTTTCTGCCTTTAGTGGTTGCTTTTTTATTTTTATTTTATGGGAGTCTTTAGCTAAAAAGAATGTTGTTATAGGTTATTATGGTAGTTCTTCAACTTTGCTTAATTTGTGTTGATCGCCAGTGCCTTACCACAGTAATTTTTTTGTGCGTGGATTATTTGTTGATTATTCTGTATTGGCTTTTTAGTTTATTGTTTAGAATATTGATTTTGTAAATCAGGGGTAGGGTGTTTTGACCTTTAAGCCTGTTTGGTGGAACTTTTAATTGGGTTGATTGGTTTTAATTGCCTTTTGCATCATGCTATATGGATGTGGTTAGTATATGGGCTATACACCGAAAAGTTTGGATCTAGTATCAAGTTGGTT

>CQ_FL

TGGCTCATTTTCATTATGTTATGTCTTTGGGTTCTTATATTAGGGTTATTATATTTTTTGTTTGGTGATGGCCTGTTATCACAGGGGTTAGCTTGAATAAGTATTTGTTACAGTGTCATTGTATAGTATCAAATGTGGGCTTTAATTTGTGTTTTTTTCCTATGCATTATTTTGGTATTTGTGGTTTACCTCGGCGTGTTTGTGTGTATGAGTCAGGGTACGCTTGAGTTAATATGCTTTGTTCAATAGGTTCTTTTGTTTCTGCCTTTAGTGGTTGCTTTTTTATTTTTATTTTATGGGAGTCTTTAGCTAAAAAGAATGTTGTTATAGGTTATTATGGTAGTTCTTCAACTTTGCTTAATTTGTGTTGATCGCCAGTGCCTTACCACAGTAATTTTTTTGTGCGCGGATTATTTGTTGATTATTCTGTATTGGCTTTTTAGTTTATTGTTTAGAATATTGATTTTGTAAATCAGGGGTAGGGTGTTTTGACCTTTAAGCCTGTTTGGTGGAACTTTTAATTGGGTTGATTGGTTTTAATTGCCTTTTGCATCATGCTATATGGATGTGGTTAGTATATGGGCTATACACCGAAAAGTTTGGATCTAGTATCAAGTTGGTT

>CQ_YuY

TGGCTCATTTTCATTATGTTATGTCTTTGGGTTCTTATATTAGGGTTATTATATTTTTTGTTTGGTGATGGCCTGTTATCACAGGGGTTAGCTTGAATAAGTATTTGTTACAGTGTCATTGTATAGTATCAAATGTGGGCTTTAATTTGTGTTTTTTTCCTATGCATTATTTTGGTATTTGTGGTTTACCTCGGCGTGTTTGTGTGTATGAGTCAGGGTACGCTTGAGTTAATATGCTTTGTTCAATAGGTTCTTTTGTTTCTGCCTTTAGTGGTTGCTTTTTTATTTTTATTTTATGGGAGTCTTTAGCTAAAAAGAATGTTGTTATAGGTTATTATGGTAGTTCTTCAACTTTGCTTAATTTGTGTTGATCGCCAGTGCCTTACCACAGTAATTTTTTTGTGCGTGGATTATTTGTTGATTATTCTGTATTGGCTTTTTAGTTTATTGTTTAGAATATTGATTTTGTAAATCAGGGGTAGGGTGTTTTGACCTTTAAGCCTGTTTGGTGGAACTTTTAATTGGGTTGATTGGTTTTAATTGCCTTTTGCATCATGCTATATGGATGTGGTTAGTATATGGGCTATAGACCGAAAAGTTTGGATCTAGTATCAAGTTGGTT

>CQ_YoY

TGGCTCATTTTCATTATGTTATGTCTTTGGGTTCTTATATTAGGATTATTATATTTTTTGTTTGGTGATGGCCTGTTATTACAGGGGTTAGCCTGAATAAGTATTTGTTACAGTGTCATTGTATAGTATCAAATGTGGGTTTTAATTTGTGTTTTTTCCCTATGCATTATTTTGGTATTTGTGGTTTACCTCGGCGTGTTTGTGTGTATGAGTCAGGGTACGCTTGAGTTAATATGCTTTGTTCAATAGGTTCTTTTGTTTCTGCCTTTAGTGGTTGCTTTTTTATTTTTATTTTATGGGAGTCTTTAGCTAAAAAGAATGTTGTTATAGGTTATTATGGTACTTCTTCAACTTTGCTTAATTTGTGTTGATCGCCAGTGCCTTATCACAGTAATTTTTTTGTGCGCGGATTATTTGTTGATTATTCTGTATTGGCTTTTTAGTTTATTGTTTAGAATATTGATTTTGTAAATCAGGGGTAGGGTGTTTTGACCTTTAAGCCTGTTTGGTGGAACTTTTAATTGGGTTGATTGGTTTTAATTGCCTTTTGCATCATGCTATATGGATGTGGTTAGTATATGGGCTATACACCGAAAAGTTTGGATCTAGTATCAAGTTGGTT

Sequenced PCR products using primers Se/Sd-7955F+Sd-8567R:

>AH_YC

GTTGGTGCTGTAGCTTTTATGTTTATTTATAAGCTATTTTGATCGAGTTATACTAAGTTGGGTTTTAGTATTTTTTTAGGGTAAATATATGGTTTTTTTATATTTCTTCTTGTTTATAATGGTTTTCTTGGGGTTTATTTTGTCTCTAACTCGTTTTCTTAGTTGTTTGATTATTTTAGAGAACTTTAAAGTGTTATTGCTATTATTCAGTTTATTACTTGGCTTACTTGATAGACATGTTTTATTTATTGCGTTGATGGTTGTTTCAACTGTTGAAGTTATTGTTGGTCTTGTTGTTTTAACGCGGGTATGAGAGTGTACAAATTCATTGGATTTGGTTTCTTTTTAGGCTTTTCTTTACTGTTTGTTATTCCTTTGTTGTATTCAGTCGGTGTTGGTGTACTTGGTTCTGTTACTGTTGGTAAAGGTTATTTTATATTTGATTCTGTCTCTTTTTATTTGATTTTATTAATATTCTTTCTGGGGGTATATAGACTTTTCTCTACCTTTTGTCG

>AH_HF

GTTGGTGCTGTAGCTTTTATGTTTATTTATAAGCTATTTTGATCGAGTTATACTAAGTTGGGTTTTAGTATTTTTTTAGGGTAAATATATGGTTTTTTTATATTTCTTCTTGTTTATAATGGTTTTCTTGGGGTTTATTTTGTCTCTAACTCGTTTTCTTAGTTGTTTGATTATTTTAGAGAACTTTAAAGTGTTATTGCTATTATTCAGTTTATTACTTGGCTTACTTGATAGACATGTTTTATTTATTGCGTTGATGGTTGTTTCAACTGTTGAAGTTATTGTTGGTCTTGTTGTTTTAACGCGGGTATGAGAGTGTACAAATTCATTGGATTTGGTTTCTTTTTAGGCTTTTCTTTACTGTTTGTTATTCCTTTGTTGTATTCAGTCGGTGTTGGTGTACTTGGTTCTGTTACTGTTGGTAAAGGTTATTTTATATTTGATTCTGTCTCTTTTTATTTGATTTTATTAATATTCTTTCTGGGGGTATATAGACTTTTCTCTACCTTTTGTCG

>AH_WH

GTTGGTGCTGTAGCTTTTATGTTTATTTATAAGCTATTTTGATCGAGTTATACTAAGTTGGGTTTTAGTATTTTTTTAGGGTAAATATATGGTTTTTTTATATTTCTTCTTGTTTATAATGGTTTTCTTGGGGTTTATTTTGTCTCTAACTCGTTTTCTTAGTTGTTTGATTATTTTAGAGAACTTTAAAGTGTTATTGCTATTATTCAGTTTATTACTTGGCTTACTTGATAGACATGTTTTATTTATTGCGTTGATGGTTGTTTCAACTGTTGAAGTTATTGTTGGTCTTGTTGTTTTAACGCGGGTATGAGAGTGTACAAATTCATTGGATTTGGTTTCTTTTTAGGCTTTTCTTTACTGTTTGTTATTCCTTTGTTGTATTCAGTCGGTGTTGGTGTACTTGGTTCTGTTACTGTTGGTAAAGGTTATTTTATATTTGATTCTGTCTCTTTTTATTTGATTTTATTAATATTCTTTCTGGGGGTATATAGACTTTTCTCTACCTTTTGTCG

>AH_LA

GTTGGTGCTGTAGCTTTTATGTTTATTTATAAGCTATTTTGATCGAGTTATACTAAGTTGGGTTTTAGTATTTTTTTAGGGTAAATATATGGTTTTTTTATATTTCTTCTTGTTTATAATGGTTTTCTTGGGGTTTATTTTGTCTCTAACTCGTTTTCTTAGTTGTTTGATTATTTTAGAGAACTTTAAAGTGTTATTGCTATTATTCAGTTTATTACTTGGCTTACTTGATAGACATGTTTTATTTATTGCGTTGATGGTTGTTTCAACTGTTGAAGTTATTGTTGGTCTTGTTGTTTTAACGCGGGTATGAGAGTGTACAAATTCATTGGATTTGGTTTCTTTTTAGGCTTTTCTTTACTGTTTGTTATTCCTTTGTTGTATTCAGTCGGTGTTGGTGTACTTGGTTCTGTTACTGTTGGTAAAGGTTATTTTATATTTGATTCTGTCTCTTTTTATTTGATTTTATTAATATTCTTTCTGGGGGTATATAGACTTTTCTCTACCTTTTGTCG

>AH_MAS

GTTGGTGCTGTAGCTTTTATGTTTATTTATAAGCTATTTTGATCGAGTTATACTAAGTTGGGTTTTAGTATTTTTTTAGGGTAAATATATGGTTTTTTTATATTTCTTCTTGTTTATAATGGTTTTCTTGGGGTTTATTTTGTCTCTAACTCGTTTTCTTAGTTGTTTGATTATTTTAGAGAACTTTAAAGTGTTATTGCTATTATTCAGTTTATTACTTGGCTTACTTGATAGACATGTTTTATTTATTGCGTTGATGGTTGTTTCAACTGTTGAAGTTATTGTTGGTCTTGTTGTTTTAACGCGGGTATGAGAGTGTACAAATTCATTGGATTTGGTTTCTTTTTAGGCTTTTCTTTACTGTTTGTTATTCCTTTGTTGTATTCAGTCGGTGTTGGTGTACTTGGTTCTGTTACTGTTGGTAAAGGTTATTTTATATTTGATTCTGTCTCTTTTTATTTGATTTTATTAATATTCTTTCTGGGGGTATATAGACTTTTCTCTACCTTTTGTCG

>AH_BB

GTTGGTGCTGTAGCTTTTATGTTTATTTATAAGCTATTTTGATCGAGTTATACTAAGTTGGGTTTTAGTATTTTTTTAGGGTAAATATATGGTTTTTTTATATTTCTTCTTGTTTATAATGGTTTTCTTGGGGTTTATTTTGTCTCTAACTCGTTTTCTTAGTTGTTTGATTATTTTAGAGAACTTTAAAGTGTTATTGCTATTATTCAGTTTATTACTTGGCTTACTTGATAGACATGTTTTATTTATTGCGTTGATGGTTGTTTCAACTGTTGAAGTTATTGTTGGTCTTGTTGTTTTAACGCGGGTATGAGAGTGTACAAATTCATTGGATTTGGTTTCTTTTTAGGCTTTTCTTTACTGTTTGTTATTCCTTTGTTGTATTCAGTCGGTGTTGGTGTACTTGGTTCTGTTACTGTTGGTAAAGGTTATTTTATATTTGATTCTGTCTCTTTTTATTTGATTTTATTAATATTCTTTCTGGGGGTATATAGACTTTTCTCTACCTTTTGTCG

>JS_SZ

GTTGGTGCTGTAGCTTTTATGTTTATTTATAAGCTATTTTGATCGAGTTATACTAAGTTGGGTTTTAGTATTTTTTTAGGGTAAATATATGGTTTTTTTATATTTCTTCTTGTTTATAATGGTTTTCTTGGGGTTTATTTTGTCTCTAACTCGTTTTCTTAGTTGTTTGATTATTTTAGAGAACTTTAAAGTGTTATTGCTATTATTCAGTTTATTACTTGGCTTACTTGATAGACATGTTTTATTTATTGCGTTGATGGTTGTTTCAACTGTTGAAGTTATTGTTGGTCTTGTTGTTTTAACGCGGGTATGAGAGTGTACAAATTCATTGGATTTGGTTTCTTTTTAGGCTTTTCTTTACTGTTTGTTATTCCTTTGTTGTATTCAGTCGGTGTTGGTGTACTTGGTTCTGTTACTGTTGGTAAAGGTTATTTTATATTTGATTCTGTCTCTTTTTATTTGATTTTATTAATATTCTTTCTGGGGGTATATAGACTTTTCTCTACCTTTTGTCG

>JS_ZJ

GTTGGTGCTGTAGCTTTTATGTTTATTTATAAGCTATTTTGATCGAGTTATACTAAGTTGGGTTTTAGTATTTTTTTAGGGTAAATATATGGTTTTTTTATATTTCTTCTTGTTTATAATGGTTTTCTTGGGGTTTATTTTGTCTCTAACTCGTTTTCTTAGTTGTTTGATTATTTTAGAGAACTTTAAAGTGTTATTGCTATTATTCAGTTTATTACTTGGCTTACTTGATAGACATGTTTTATTTATTGCGTTGATGGTTGTTTCAACTGTTGAAGTTATTGTTGGTCTTGTTGTTTTAACGCGGGTATGAGAGTGTACAAATTCATTGGATTTGGTTTCTTTTTAGGCTTTTCTTTACTGTTTGTTATTCCTTTGTTGTATTCAGTCGGTGTTGGTGTACTTGGTTCTGTTACTGTTGGTAAAGGTTATTTTATATTTGATTCTGTCTCTTTTTATTTGATTTTATTAATATTCTTTCTGGGGGTATATAGACTTTTCTCTACCTTTTGTCG

>JS_YC

GTTGGTGCTGTAGCTTTTATGTTTATTTATAAGCTATTTTGATCGAGTTATACTAAGTTGGGTTTTAGTATTTTTTTAGGGTAAATATATGGTTTTTTTATATTTCTTCTTGTTTATAATGGTTTTCTTGGGGTTTATTTTGTCTCTAACTCGTTTTCTTAGTTGTTTGATTATTTTAGAGAACTTTAAAGTGTTATTGCTATTATTCAGTTTATTACTTGGCTTACTTGATAGACATGTTTTATTTATTGCGTTGATGGTTGTTTCAACTGTTGAAGTTATTGTTGGTCTTGTTGTTTTAACGCGGGTATGAGAGTGTACAAATTCATTGGATTTGGTTTCTTTTTAGGCTTTTCTTTACTGTTTGTTATTCCTTTGTTGTATTCAGTCGGTGTTGGTGTACTTGGTTCTGTTACTGTTGGTAAAGGTTATTTTATATTTGATTCTGTCTCTTTTTATTTGATTTTATTAATATTCTTTCTGGGGGTATATAGACTTTTCTCTACCTTTTGTCG

>ZJ_JX

GTTGGTGCTGTAGCTTTTATGTTTATTTATAAGCTATTTTGATCGAGTTATACTAAGTTGGGTTTTAGTATTTTTTTAGGGTAAATATATGGTTTTTTTATATTTCTTCTTGTTTATAATGGTTTTCTTGGGGTTTATTTTGTCTCTAACTCGTTTTCTTAGTTGTTTGATTATTTTAGAGAACTTTAAAGTGTTATTGCTATTATTCAGTTTATTACTTGGCTTACTTGATAGACATGTTTTATTTATTGCGTTGATGGTTGTTTCAACTGTTGAAGTTATTGTTGGTCTTGTTGTTTTAACGCGGGTATGAGAGTGTACAAATTCATTGGATTTGGTTTCTTTTTAGGCTTTTCTTTACTGTTTGTTATTCCTTTGTTGTATTCAGTCGGTGTTGGTGTACTTGGTTCTGTTACTGTTGGTAAAGGTTATTTTATATTTGATTCTGTCTCTTTTTATTTGATTTTATTAATATTCTTTCTGGGGGTATATAGACTTTTCTCTACCTTTTGTCG

>ZJ_NB

GTTGGTGCTGTAGCTTTTATGTTTATTTATAAGCTATTTTGATCGAGTTATACTAAGTTGGGTTTTAGTATTTTTTTAGGGTAAATATATGGTTTTTTTATATTTCTTCTTGTTTATAATGGTTTTCTTGGGGTTTATTTTGTCTCTAACTCGTTTTCTTAGTTGTTTGATTATTTTAGAGAACTTTAAAGTGTTATTGCTATTATTCAGTTTATTACTTGGCTTACTTGATAGACATGTTTTATTTATTGCGTTGATGGTTGTTTCAACTGTTGAAGTTATTGTTGGTCTTGTTGTTTTAACGCGGGTATGAGAGTGTACAAATTCATTGGATTTGGTTTCTTTTTAGGCTTTTCTTTACTGTTTGTTATTCCTTTGTTGTATTCAGTCGGTGTTGGTGTACTTGGTTCTGTTACTGTTGGTAAAGGTTATTTTATATTTGATTCTGTCTCTTTTTATTTGATTTTATTAATATTCTTTCTGGGGGTATATAGACTTTTCTCTACCTTTTGTCG

>ZJ_SX

GTTGGTGCTGTAGCTTTTATGTTTATTTATAAGCTATTTTGATCGAGTTATACTAAGTTGGGTTTTAGTATTTTTTTAGGGTAAATATATGGTTTTTTTATATTTCTTCTTGTTTATAATGGTTTTCTTGGGGTTTATTTTGTCTCTAACTCGTTTTCTTAGTTGTTTGATTATTTTAGAGAACTTTAAAGTGTTATTGCTATTATTCAGTTTATTACTTGGCTTACTTGATAGACATGTTTTATTTATTGCGTTGATGGTTGTTTCAACTGTTGAAGTTATTGTTGGTCTTGTTGTTTTAACGCGGGTATGAGAGTGTACAAATTCATTGGATTTGGTTTCTTTTTAGGCTTTTCTTTACTGTTTGTTATTCCTTTGTTGTATTCAGTCGGTGTTGGTGTACTTGGTTCTGTTACTGTTGGTAAAGGTTATTTTATATTTGATTCTGTCTCTTTTTATTTGATTTTATTAATATTCTTTCTGGGGGTATATAGACTTTTCTCTACCTTTTGTCG

>ZJ_WZ

GTTGGTGCTGTAGCTTTTATGTTTATTTATAAGCTATTTTGATCGAGTTATACTAAGTTGGGTTTTAGTATTTTTTTAGGGTAAATATATGGTTTTTTTATATTTCTTCTTGTTTATAATGGTTTTCTTGGGGTTTATTTTGTCTCTAACTCGTTTTCTTAGTTGTTTGATTATTTTAGAGAACTTTAAAGTGTTATTGCTATTATTCAGTTTATTACTTGGCTTACTTGATAGACATGTTTTATTTATTGCGTTGATGGTTGTTTCAACTGTTGAAGTTATTGTTGGTCTTGTTGTTTTAACGCGGGTATGAGAGTGTACAAATTCATTGGATTTGGTTTCTTTTTAGGCTTTTCTTTACTGTTTGTTATTCCTTTGTTGTATTCAGTCGGTGTTGGTGTACTTGGTTCTGTTACTGTTGGTAAAGGTTATTTTATATTTGATTCTGTCTCTTTTTATTTGATTTTATTAATATTCTTTCTGGGGGTATATAGACTTTTCTCTACCTTTTGTCG

>ZJ_ZJ

GTTGGTGCTGTAGCTTTTATGTTTATTTATAAGCTATTTTGATCGAGTTATACTAAGTTGGGTTTTAGTATTTTTTTAGGGTAAGTATATGGTTTTTTTATATTTCTTCTTGTTTATAATGGTTTTCTTGGGGTTTATTTTGTCTCTAACTCGTTTTCTTAGTTGTTTGATTATTTTAGAGAACTTTAAAGTGTTATTGCTATTATTCAGTTTATTACTTGGCTTACTTGATAGACATGTTTTATTTATTGCGTTGATGGTTGTTTCAACTGTTGAAGTTATTGTTGGTCTTGTTGTTTTAACGCGGGTATGAGAGTGTACAAATTCATTGGATTTGGTTTCTTTTTAGGCTTTTCTTTACTGTTTGTTATTCCTTTGTTGTATTCAGTCGGTGTTGGTGTACTTGGTTCTGTTACTGTTGGTAAAGGTTATTTTATATTTGATTCTGTCTCTTTTTATTTGATTTTATTAATATTCTTTCTGGGGGTATATAGACTTTTCTCTACCTTTTGTCG

>JX_FZ

GTTGGTGCTGTAGCTTTTATGTTTATTTATAAGCTATTTTGATCGAGTTATACTAAGTTGGGTTTTAGTATTTTTTTAGGGTAAATATATGGTTTTTTTATATTTCTTCTTGTTTATAATGGTTTTCTTGGGGTTTATTTTGTCTCTAACTCGTTTTCTTAGTTGTTTGATTATTTTAGAGAACTTTAAAGTGTTATTGCTATTATTCAGTTTATTACTTGGCTTACTTGATAGACATGTTTTATTTATTGCGTTGATGGTTGTTTCAACTGTTGAAGTTATTGTTGGTCTTGTTGTTTTAACGCGGGTATGAGAGTGTACAAATTCATTGGATTTGGTTTCTTTTTAGGCTTTTCTTTACTGTTTGTTATTCCTTTGTTGTATTCAGTCGGTGTTGGTGTACTTGGTTCTGTTACTGTTGGTAAAGGTTATTTTATATTTGATTCTGTTTCTTTTTATTTGATTTTATTAATATTCTTTCTGGGGGTATATAGACTTTTCTCTACCTTTTGTCG

>JX_JJ

GTTGGTGCTGTAGCTTTTATGTTTATTTATAAGCTATTTTGATCGAGTTATACTAAGTTGGGTTTTAGTATTTTTTTAGGGTAAATATATGGTTTTTTTATATTTCTTCTTGTTTATAATGGTTTTCTTGGGGTTTATTTTGTCTCTAACTCGTTTTCTTAGTTGTTTGATTATTTTAGAGAACTTTAAAGTGTTATTGCTATTATTCAGTTTATTACTTGGCTTACTTGATAGACATGTTTTATTTATTGCGTTGATGGTTGTTTCAACTGTTGAAGTTATTGTTGGTCTTGTTGTTTTAACGCGGGTATGAGAGTGTACAAATTCATTGGATTTGGTTTCTTTTTAGGCTTTTCTTTACTGTTTGTTATTCCTTTGTTGTATTCAGTCGGTGTTGGTGTACTTGGTTCTGTTACTGTTGGTAAAGGTTATTTTATATTTGATTCTGTCTCTTTTTATTTGATTTTATTAATATTCTTTCTGGGGGTATATAGACTTTTCTCTACCTTTTGTCG

>JX_JA

GTTGGTGCTGTAGCTTTTATGTTTATTTATAAGCTATTTTGATCGAGTTATACTAAGTTGGGTTTTAGTATTTTTTTAGGGTAAATATATGGTTTTTTTATATTTCTTCTTGTTTATAATGGTTTTCTTGGGGTTTATTTTGTCTCTAACTCGTTTTCTTAGTTGTTTGATTATTTTAGAGAACTTTAAAGTGTTATTGCTATTATTCAGTTTATTACTTGGCTTACTTGATAGACATGTTTTATTTATTGCGTTGATGGTTGTTTCAACTGTTGAAGTTATTGTTGGTCTTGTTGTTTTAACGCGGGTATGAGAGTGTACAAATTCATTGGATTTGGTTTCTTTTTAGGCTTTTCTTTACTGTTTGTTATTCCTTTGTTGTATTCAGTCGGTGTTGGTGTACTTGGTTCTGTTACTGTTGGTAAAGGTTATTTTATATTTGATTCTGTCTCTTTTTATTTGATTTTATTAATATTCTTTCTGGGGGTATATAGACTTTTCTCTACCTTTTGTCG

>JX_YC

GTTGGTGCTGTAGCTTTTATGTTTATTTATAAGCTATTTTGATCGAGTTATACTAAGTTGGGTTTTAGTATTTTTTTAGGGTAAATATATGGTTTTTTTATATTTCTTCTTGTTTATAATGGTTTTCTTGGGGTTTATTTTGTCTCTAACTCGTTTTCTTAGTTGTTTGATTATTTTAGAGAACTTTAAAGTGTTATTGCTATTATTCAGTTTATTACTTGGCTTACTTGATAGACATGTTTTATTTATTGCGTTGATGGTTGTTTCAACTGTTGAAGTTATTGTTGGTCTTGTTGTTTTAACGCGGGTATGAGAGTGTACAAATTCATTGGATTTGGTTTCTTTTTAGGCTTTTCTTTACTGTTTGTTATTCCTTTGTTGTATTCAGTCGGTGTTGGTGTACTTGGTTCTGTTACTGTTGGTAAAGGTTATTTTATATTTGATTCTGTCTCTTTTTATTTGATTTTATTAATATTCTTTCTGGGGGTATATAGACTTTTCTCTACCTTTTGTCG

>FJ_ND

GTTGGTGCTGTAGCTTTTATGTTTATTTATAAGCTATTTTGATCGAGTTATACTAAGTTGGGTTTTAGTATTTTTTTAGGGTAAATATATGGTTTTTTTATATTTCTTCTTGTTTATAATGGTTTTCTTGGGGTTTATTTTGTCTCTAACTCGTTTTCTTAGTTGTTTGATTATTTTAGAGAACTTTAAAGTGTTATTGCTATTATTCAGTTTATTACTTGGCTTACTTGATAGACATGTTTTATTTATTGCGTTGATGGTTGTTTCAACTGTTGAAGTTATTGTTGGTCTTGTTGTTTTAACGCGGGTATGAGAGTGTACAAATTCATTGGATTTGGTTTCTTTTTAGGCTTTTCTTTACTGTTTGTTATTCCTTTGTTGTATTCAGTCGGTGTTGGTGTACTTGGTTCTGTTACTGTTGGTAAAGGTTATTTTATATTTGATTCTGTCTCTTTTTATTTGATTTTATTAATATTCTTTCTGGGGGTATATAGACTTTTCTCTACCTATTGTCG

>FJ_QZ

GTTGGTGCTGTAGCTTTTATGTTTATTTATAAGCTATTTTGATCGAGTTATACTAAGTTGGGTTTTAGTATTTTTTTAGGATAAGTATATGGTTTTTTTATATTTCTTCTTGTTTATAATGGTTTTCTTGGGGTTTATTTTGTCTCTAACTCGTTTTCTTAGTTGTTTGATTATTTTAGAGAACTTTAAAGTGTTATTGCTATTATTCAGTTTATTACTTGGCTTATTTGATAGACATGTTTTATTTATTGCGTTGATGGTTGTTTCAACTGTTGAAGTTATTGTTGGTCTTGTTGTTTTAACACGGGTATGAGAGTGTACAAATTCATTGGATTTGGTTTCTTTTTAGGCTTTTCTTTACTGTTTGTTATTCCCTTATTGTATTCAGTCGGTGTTGGTGTACTTGGTTCTGTTACTGTTGGTAAAGGTTATTTTATATTTGATTCTGTTTCTTTTTATTTGATTTTATTAATATTCTTTCTGGGGGTGTATAGACTTTTCTCTACCTTTTGTCG

>FJ_NP

GTTGGTGCTGTAGCTTTTATGTTTATTTATAAGCTATTTTGATCGAGTTATACTAAGTTGGGTTTTAGTATTTTTTTAGGGTAAATATATGGTTTTTTTATATTTCTTCTTGTTTATAATGGTTTTCTTGGGGTTTATTTTGTCTCTAACTCGTTTTCTTAGTTGTTTGATTATTTTAGAGAACTTTAAAGTGTTATTGCTATTATTCAGTTTATTACTTGGCTTACTTGATAGACATGTTTTATTTATTGCGTTGATGGTTGTTTCAACTGTTGAAGTTATTGTTGGTCTTGTTGTTTTAACGCGGGTATGAGAGTGTACAAATTCATTGGATTTGGTTTCTTTTTAGGCTTTTCTTTACTGTTTGTTATTCCTTTGTTGTATTCAGTCGGTGTTGGTGTACTTGGTTCTGTTACTGTTGGTAAAGGTTATTTTATATTTGATTCTGTCTCTTTTTATTTGATTTTATTAATATTCTTTCTGGGGGTATATAGACTTTTCTCTACCTTTTGTCG

>SH_NH

GTTGGTGCTGTAGCTTTTATGTTTATTTATAAGCTATTTTGATCGAGTTATACTAAGTTGGGTTTTAGTATTTTTTTAGGGTAAATATATGGTTTTTTTATATTTCTTCTTGTTTATAATGGTTTTCTTGGGGTTTATTTTGTCTCTAACTCGTTTTCTTAGTTGTTTGATTATTTTAGAGAACTTTAAAGTGTTATTGCTATTATTCAGTTTATTACTTGGCTTACTTGATAGACATGTTTTATTTATTGCGTTGATGGTTGTTTCAACTGTTGAAGTTATTGTTGGTCTTGTTGTTTTAACGCGGGTATGAGAGTGTACAAATTCATTGGATTTGGTTTCTTTTTAGGCTTTTCTTTACTGTTTGTTATTCCTTTGTTGTATTCAGTCGGTGTTGGTGTACTTGGTTCTGTTACTGTTGGTAAAGGTTATTTTATATTTGATTCTGTCTCTTTTTATTTGATTTTATTAATATTCTTTCTGGGGGTATATAGACTTTTCTCTACCTTTTGTCG

>SH_HP

GTTGGTGCTGTAGCTTTTATGTTTATTTATAAGCTATTTTGATCGAGTTATACTAAGTTGGGTTTTAGTATTTTTTTAGGGTAAATATATGGTTTTTTTATATTTCTTCTTGTTTATAATGGTTTTCTTGGGGTTTATTTTGTCTCTAACTCGTTTTCTTAGTTGTTTGATTATTTTAGAGAACTTTAAAGTGTTATTGCTATTATTCAGTTTATTACTTGGCTTACTTGATAGACATGTTTTATTTATTGCGTTGATGGTTGTTTCAACTGTTGAAGTTATTGTTGGTCTTGTTGTTTTAACGCGGGTATGAGAGTGTACAAATTCATTGTATTTGGTTTCTTTTTAGGCTTTTCTTTACTGTTTGTTATTCCTTTGTTGTATTCAGTCGGTGTTGGTGTACTTGGTTCTGTTACTGTTGGTAAAGGTTATTTTATATTTGATTCTGTCTCTTTTTATTTGATTTTATTAATATTCTTTCTGGGGGTATATAGACTTTTCTCTACCTTTTGTCG

>HeN_ZZ

GTTGGTGCTGTAGCTTTTATGTTTATTTATAAGCTATTTTGATCGAGTTATACTAAGTTGGGTTTTAGTATTTTTTTAGGGTAAATATATGGTTTTTTTATATTTCTTCTTGTTTATAATGGTTTTCTTGGGGTTTATTTTGTCTCTAACTCGTTTTCTTAGTTGTTTGATTATTTTAGAGAACTTTAAAGTGTTATTGCTATTATTCAGTTTATTACTTGGCTTACTTGATAGACATGTTTTATTTATTGCGTTGATGGTTGTTTCAACTGTTGAAGTTATTGTTGGTCTTGTTGTTTTAACGCGGGTATGAGAGTGTACAAATTCATTGGATTTGGTTTCTTTTTAGGCTTTTCTTTACTGTTTGTTATTCCTTTGTTGTATTCAGTCGGTGTTGGTGTACTTGGTTCTGTTACTGTTGGTAAAGGTTATTTTATATTTGATTCTGTCTCTTTTTATTTGATTTTATTAATATTCTTTCTGGGGGTATATAGACTTTTCTCTACCTTTTGTCG

>HeN_XX

GTTGGTGCTGTAGCTTTTATGTTTATTTATAAGCTATTTTGATCGAGTTATACTAAGTTGGGTTTTAGTATTTTTTTAGGGTAAATATATGGTTTTTTTATATTTCTTCTTGTTTATAATGGTTTTCTTGGGGTTTATTTTGTCTCTAACTCGTTTTCTTAGTTGTTTGATTATTTTAGAGAACTTTAAAGTGTTATTGCTATTATTCAGTTTATTACTTGGCTTACTTGATAGACATGTTTTATTTATTGCGTTGATGGTTGTTTCAACTGTTGAAGTTATTGTTGGTCTTGTTGTTTTAACGCGGGTATGAGAGTGTACAAATTCATTGGATTTGGTTTCTTTTTAGGCTTTTCTTTACTGTTTGTTATTCCTTTGTTGTATTCAGTCGGTGTTGGTGTACTTGGTTCTGTTACTGTTGGTAAAGGTTATTTTATATTTGATTCTGTCTCTTTTTATTTGATTTTATTAATATTCTTTCTGGGGGTATATAGACTTTTCTCTACCTTTTGTCG

>HeN_KF

GTTGGTGCTGTAGCTTTTATGTTTATTTATAAGCTATTTTGATCGAGTTATACTAAGTTGGGTTTTAGTATTTTTTTAGGGTAAATATATGGTTTTTTTATATTTCTTCTTGTTTATAATGGTTTTCTTGGGGTTTATTTTGTCTCTAACTCGTTTTCTTAGTTGTTTGATTATTTTAGAGAACTTTAAAGTGTTATTGCTATTATTCAGTTTATTACTTGGCTTACTTGATAGACATGTTTTATTTATTGCGTTGATGGTTGTTTCAACTGTTGAAGTTATTGTTGGTCTTGTTGTTTTAACGCGGGTATGAGAGTGTACAAATTCATTGGATTTGGTTTCTTTTTAGGCTTTTCTTTACTGTTTGTTATTCCTTTGTTGTATTCAGTCGGTGTTGGTGTACTTGGTTCTGTTACTGTTGGTAAAGGTTATTTTATATTTGATTCTGTCTCTTTTTATTTGATTTTATTAATATTCTTTCTGGGGGTATATAGACTTTTCTCTACCTTTTGTCG

>HeN_ZK

GTTGGTGCTGTAGCTTTTATGTTTATTTATAAGCTATTTTGATCGAGTTATACTAAGTTGGGTTTTAGTATTTTTTTAGGGTAAATATATGGTTTTTTTATATTTCTTCTTGTTTATAATGGTTTTCTTGGGGTTTATTTTGTCTCTAACTCGTTTTCTTAGTTGTTTGATTATTTTAGAGAACTTTAAAGTGTTATTGCTATTATTCAGTTTATTACTTGGCTTACTTGATAGACATGTTTTATTTATTGCGTTGATGGTTGTTTCAACTGTTGAAGTTATTGTTGGTCTTGTTGTTTTAACGCGGGTATGAGAGTGTACAAATTCATTGGATTTGGTTTCTTTTTAGGCTTTTCTTTACTGTTTGTTATTCCTTTGTTGTATTCAGTCGGTGTTGGTGTACTTGGTTCTGTTACTGTTGGTAAAGGTTATTTTATATTTGATTCTGTCTCTTTTTATTTGATTTTATTAATATTCTTTCTGGGGGTATATAGACTTTTCTCTACCTTTTGTCG

>HeN_LH

GTTGGTGCTGTAGCTTTTATGTTTATTTATAAGCTATTTTGATCGAGTTATACTAAGTTGGGTTTTAGTATTTTTTTAGGGTAAATATATGGTTTTTTTATATTTCTTCTTGTTTATAATGGTTTTCTTGGGGTTTATTTTGTCTCTAACTCGTTTTCTTAGTTGTTTGATTATTTTAGAGAACTTTAAAGTGTTATTGCTATTATTCAGTTTATTACTTGGCTTACTTGATAGACATGTTTTATTTATTGCGTTGATGGTTGTTTCAACTGTTGAAGTTATTGTTGGTCTTGTTGTTTTAACGCGGGTATGAGAGTGTACAAATTCATTGGATTTGGTTTCTTTTTAGGCTTTTCTTTACTGTTTGTTATTCCTTTGTTGTATTCAGTCGGTGTTGGTGTACTTGGTTCTGTTACTGTTGGTAAAGGTTATTTTATATTTGATTCTGTCTCTTTTTATTTGATTTTATTAATATTCTTTCTGGGGGTATATAGACTTTTCTCTACCTTTTGTCG

>HeN_NY

GTTGGTGCTGTAGCTTTTATGTTTATTTATAAGCTATTTTGATCGAGTTATACTAAGTTGGGTTTTAGTATTTTTTTAGGGTAAATATATGGTTTTTTTATATTTCTTCTTGTTTATAATGGTTTTCTTGGGGTTTATTTTGTCTCTAACTCGTTTTCTTAGTTGTTTGATTATTTTAGAGAACTTTAAAGTGTTATTGCTATTATTCAGTTTATTACTTGGCTTACTTGATAGACATGTTTTATTTATTGCGTTGATGGTTGTTTCAACTGTTGAAGTTATTGTTGGTCTTGTTGTTTTAACGCGGGTATGAGAGTGTACAAATTCATTGGATTTGGTTTCTTTTTAGGCTTTTCTTTACTGTTTGTTATTCCTTTGTTGTATTCAGTCGGTGTTGGTGTACTTGGTTCTGTTACTGTTGGTAAAGGTTATTTTATATTTGATTCTGTCTCTTTTTATTTGATTTTATTAATATTCTTTCTGGGGGTATATAGACTTTTCTCTACCTTTTGTCG

>HeN_XY

GTTGGTGCTGTAGCTTTTATGTTTATTTATAAGCTATTTTGATCGAGTTATACTAAGTTGGGTTTTAGTATTTTTTTAGGGTAAATATATGGTTTTTTTATATTTCTTCTTGTTTATAATGGTTTTCTTGGGGTTTATTTTGTCTCTAACTCGTTTTCTTAGTTGTTTGATTATTTTAGAGAACTTTAAAGTGTTATTGCTATTATTCAGTTTATTACTTGGCTTACTTGATAGACATGTTTTATTTATTGCGTTGATGGTTGTTTCAACTGTTGAAGTTATTGTTGGTCTTGTTGTTTTAACGCGGGTATGAGAGTGTACAAATTCATTGGATTTGGTTTCTTTTTAGGCTTTTCTTTACTGTTTGTTATTCCTTTGTTGTATTCAGTCGGTGTTGGTGTACTTGGTTCTGTTACTGTTGGTAAAGGTTATTTTATATTTGATTCTGTCTCTTTTTATTTGATTTTATTAATATTCTTTCTGGGGGTATATAGACTTTTCTCTACCTTTTGTCG

>HuB_XG

GTTGGTGCTGTAGCTTTTATGTTTATTTATAAGCTATTTTGATCGAGTTATACTAAGTTGGGTTTTAGTATTTTTTTAGGGTAAATATATGGTTTTTTTATATTTCTTCTTGTTTATAATGGTTTTCTTGGGGTTTATTTTGTCTCTAACTCGTTTTCTTAGTTGTTTGATTATTTTAGAGAACTTTAAAGTGTTATTGCTATTATTCAGTTTATTACTTGGCTTACTTGATAGACATGTTTTATTTATTGCGTTGATGGTTGTTTCAACTGTTGAAGTTATTGTTGGTCTTGTTGTTTTAACGCGGGTATGAGAGTGTACAAATTCATTGGATTTGGTTTCTTTTTAGGCTTTTCTTTACTGTTTGTTATTCCTTTGTTGTATTCAGTCGGTGTTGGTGTACTTGGTTCTGTTACTGTTGGTAAAGGTTATTTTATATTTGATTCTGTCTCTTTTTATTTGATTTTATTAATATTCTTTCTGGGGGTATATAGACTTTTCTCTACCTTTTGTCG

>HuB_XN

GTTGGTGCTGTAGCTTTTATGTTTATTTATAAGCTATTTTGATCGAGTTATACTAAGTTGGGTTTTAGTATTTTTTTAGGGTAAATATATGGTTTTTTTATATTTCTTCTTGTTTATAATGGTTTTCTTGGGGTTTATTTTGTCTCTAACTCGTTTTCTTAGTTGTTTGATTATTTTAGAGAACTTTAAAGTGTTATTGCTATTATTCAGTTTATTACTTGGCTTACTTGATAGACATGTTTTATTTATTGCGTTGATGGTTGTTTCAACTGTTGAAGTTATTGTTGGTCTTGTTGTTTTAACGCGGGTATGAGAGTGTACAAATTCATTGGATTTGGTTTCTTTTTAGGCTTTTCTTTACTGTTTGTTATTCCTTTGTTGTATTCAGTCGGTGTTGGTGTACTTGGTTCTGTTACTGTTGGTAAAGGTTATTTTATATTTGATTCTGTCTCTTTTTATTTGATTTTATTAATATTCTTTCTGGGGGTATATAGACTTTTCTCTACCTTTTGTCG

>HuB_HG

GTTGGTGCTGTAGCTTTTATGTTTATTTATAAGCTATTTTGATCGAGTTATACTAAGTTGGGTTTTAGTATTTTTTTAGGGTAAATATATGGTTTTTTTATATTTCTTCTTGTTTATAATGGTTTTCTTGGGGTTTATTTTGTCTCTAACTCGTTTTCTTAGTTGTTTGATTATTTTAGAGAACTTTAAAGTGTTATTGCTATTATTCAGTTTATTACTTGGCTTACTTGATAGACATGTTTTATTTATTGCGTTGATGGTTGTTTCAACTGTTGAAGTTATTGTTGGTCTTGTTGTTTTAACGCGGGTATGAGAGTGTACAAATTCATTGGATTTGGTTTCTTTTTAGGCTTTTCTTTACTGTTTGTTATTCCTTTGTTGTATTCAGTCGGTGTTGGTGTACTTGGTTCTGTTACTGTTGGTAAAGGTTATTTTATATTTGATTCTGTCTCTTTTTATTTGATTTTATTAATATTCTTTCTGGGGGTATATAGACTTTTCTCTACCTTTTGTCG

>HuN_ZJJ

GTTGGTGCTGTAGCTTTTATGTTTATTTATAAGCTATTTTGATCGAGTTATACTAAGTTGGGTTTTAGTATTTTTTTAGGATAAGTATATGGTTTTTTTATATTTCTTCTTGTTTATAATGGTTTTCTTGGGGTTTATTTTGTCTCTAACTCGTTTTCTTAGTTGTTTGATTATTTTAGAGAACTTTAAAGTGTTATTGCTATTATTCAGTTTATTACTTGGCTTATTTGATAGACATGTTTTATTTATTGCGTTGATGGTTGTTTCAACTGTTGAAGTTATTGTTGGTCTTGTTGTTTTAACACGGGTATGAGAGTGTACAAATTCATTGGATTTGGTTTCTTTTTAGGCTTTTCTTTACTGTTTGTTATTCCCTTATTGTATTCAGTCGGTGTTGGTGTACTTGGTTCTGTTACTGTTGGTAAAGGTTATTTTATATTTGATTCTGTTTCTTTTTATTTGATTTTATTAATATTCTTTCTGGGGGTGTATAGACTTTTCTCTACCTTTTGTCG

>HuN_HH

GTTGGTGCTGTAGCTTTTATGTTTATTTATAAGCTATTTTGATCGAGTTATACTAAGTTGGGTTTTAGTATTTTTTTAGGATAAGTATATGGTTTTTTTATATTTCTTCTTGTTTATAATGGTTTTCTTGGGGTTTATTTTGTCTCTAACTCGTTTTCTTAGTTGTTTGATTATTTTAGAGAACTTTAAAGTGTTATTGCTATTATTCAGTTTATTACTTGGCTTATTTGATAGACATGTTTTATTTATTGCGTTGATGGTTGTTTCAACTGTTGAAGTTATTGTTGGTCTTGTTGTTTTAACACGGGTATGAGAGTGTACAAATTCATTGGATTTGGTTTCTTTTTAGGCTTTTCTTTACTGTTTGTTATTCCCTTATTGTATTCAGTCGGTGTTGGTGTACTTGGTTCTGTTACTGTTGGTAAAGGTTATTTTATATTTGATTCTGTTTCTTTTTATTTGATTTTATTAATATTCTTTCTGGGGGTGTATAGACTTTTCTCTACCTTTTGTCG

>HuN_YY

GTTGGTGCTGTAGCTTTTATGTTTATTTATAAGCTATTTTGATCGAGTTATACTAAGTTGGGTTTTAGTATTTTTTTAGGATAAATATATGGTTTTTTTATATTTCTTCTTGTTTATAATGGTTTTCTTGGGGTTTATTTTGTCTCTAACTCGTTTTCTTAGTTGTTTGATTATTTTAGAGAACTTTAAAGTGTTATTGCTATTATTCAGTTTATTACTTGGCTTATTTGATAGACATGTTTTATTTATTGCGTTGATGGTTGTTTCAACTGTTGAAGTTATTGTTGGTCTTGTTGTTTTAACACGGGTATGAGAGTGTACAAATTCATTGGATTTGGTTTCTTTTTAGGCTTTTCTTTACTGTTTGTTATTCCCTTATTGTATTCAGTTGGTGTTGGTGTACTTGGTTCTGTTACTGTTGGTAAAGGTTATTTTATATTTGATTCTGTTTCTTTTTATTTGATTTTATTAATATTCTTTCTGGGGG----------------------------

>HuN_HY

GTTGGTGCTGTAGCTTTTATGTTTATTTATAAGCTATTTTGATCGAGTTATACTAAGTTGGGTTTTAGTATTTTTTTAGGGTAAATATATGGTTTTTTTATATTTCTTCTTGTTTATAATGGTTTTCTTGGGGTTTATTTTGTCTCTAACTCGTTTTCTTAGTTGTTTGATTATTTTAGAGAACTTTAAAGTGTTATTGCTATTATTCAGTTTATTACTTGGCTTACTTGATAGACATGTTTTATTTATTGCGTTGATGGTTGTTTCAACTGTTGAAGTTATTGTTGGTCTTGTTGTTTTAACGCGGGTATGAGAGTGTACAAATTCATTGGATTTGGTTTCTTTTTAGGCTTTTCTTTACTGTTTGTTATTCCTTTGTTGTATTCAGTCGGTGTTGGTGTACTTGGTTCTGTTACTGTTGGTAAAGGTTATTTTATATTTGATTCTGTCTCTTTTTATTTGATTTTATTAATATTCTTTCTGGGGGTATATAGACTTTTCTCTACCTTTTGTCG

>HuN_XT

GTTGGTGCTGTAGCTTTTATGTTTATTTATAAGCTATTTTGATCGAGTTATACTAAGTTGGGTTTTAGTATTTTTTTAGGATAAATATATGGTTTTTTTATATTTCTTCTTGTTTATAATGGTTTTCTTGGGGTTTATTTTGTCTCTAACTCGTTTTCTTAGTTGTTTGATTATTTTAGAGAACTTTAAAGTGTTATTGCTATTATTCAGTTTATTACTTGGCTTATTTGATAGACATGTTTTATTTATTGCGTTGATGGTTGTTTCAACTGTTGAAGTTATTGTTGGTCTTGTTGTTTTAACACGGGTATGAGAGTGTACAAATTCATTGGATTTGGTTTCTTTTTAGGCTTTTCTTTACTGTTTGTTATTCCTTTATTGTATTCAGTCGGTGTTGGTGTACTTGGTTCTGTTATTGTTGGTAAAGGTTATTTTATATTTGATTCTGTTTCTTTTTATTTGATTTTATTAATATTCTTTCTGGGGGTGTATAGACTTTTCTCTACCTTTTGTCG

>HuN_SY

GTTGGTGCTGTAGCTTTTATGTTTATTTATAAGCTATTTTGATCGAGTTATACTAAGTTGGGTTTTAGTATTTTTTTAGGATAAATATATGGTTTTTTTATATTTCTTCTTGTTTATAATGGTTTTCTTGGGGTTTATTTTGTCTCTAACTCGTTTTCTTAGTTGTTTGATTATTTTAGAGAACTTTAAAGTGTTATTGCTATTATTCAGTTTATTACTTGGCTTATTTGATAGACATGTTTTATTTATTGCGTTGATGGTTGTTTCAACTGTTGAAGTTATTGTTGGTCTTGTTGTTTTAACACGGGTATGAGAGTGTACAAATTCATTGGATTTGGTTTCTTTTTAGGCTTTTCTTTACTGTTTGTTATTCCTTTATTGTATTCAGTCGGTGTTGGTGTACTTGGTTCTGTTATTGTTGGTAAAGGTTATTTTATATTTGATTCTGTTTCTTTTTATTTGATTTTATTAATATTCTTTCTGGGGGTGTATAGACTTTTCTCTACCTTTTGTCG

>HuN_XX

GTTGGTGCTGTAGCTTTTATGTTTATTTATAAGCTATTTTGATCGAGTTATACTAAGTTGGGTTTTAGTATTTTTTTAGGATAAGTATATGGTTTTTTTATATTTCTTCTTGTTTATAATGGTTTTCTTGGGGTTTATTTTGTCTCTAACTCGTTTTCTTAGTTGTTTGATTATTTTAGAGAACTTTAAAGTGTTATTGCTATTATTCAGTTTATTACTTGGCTTATTTGATAGACATGTTTTATTTATTGCGTTGATGGTTGTTTCAACTGTTGAAGTTATTGTTGGTCTTGTTGTTTTAACACGGGTATGAGAGTGTACAAATTCATTGGATTTGGTTTCTTTTTAGGCTTTTCTTTACTGTTTGTTATTCCCTTATTGTATTCAGTCGGTGTTGGTGTACTTGGTTCTGTTACTGTTGGTAAAGGTTATTTTATATTTGATTCTGTTTCTTTTTATTTGATTTTATTAATATTCTTTCTGGGGGTGTATAGACTTTTCTCTACCTTTTGTCG

>HuN_CS

GTTGGTGCTGTAGCTTTTATGTTTATTTATAAGCTATTTTGATCGAGTTATACTAAGTTGGGTTTTAGTATTTTTTTAGGATAAATATATGGTTTTTTTATATTTCTTCTTGTTTATAATGGTTTTCTTGGGGTTTATTTTGTCTCTAACTCGTTTTCTTAGTTGTTTGATTATTTTAGAGAACTTTAAAGTGTTATTGCTATTATTCAGTTTATTACTTGGCTTACTTGATAGACATGTTTTATTTATTGCGTTGATGGTTGTTTCAACTGTTGAAGTTATTGTTGGTCTTGTTGTTTTAACGCGGGTATGAGAGTGTACAAATTCATTGGATTTGGTTTCTTTTTAGGCTTTTCTTTACTGTTTGTTATTCCTTTGTTGTATTCAGTCGGTGTTGGTGTACTTGGTTCTGTTACTGTTGGTAAAGGTTATTTTATATTTGATTCTGTCTCTTTTTATTTGATTTTATTAATATTCTTTCTGGGGGTATATAGACTTTTCTCTACCTTTTGTCG

>GD_DG

GTTGGTGCTGTAGCTTTTATGTTTATTTATAAGCTATTTTGATCGAGTTATACTAAGTTGGGTTTTAGTATTTTTTTAGGGTAAATATATGGTTTTTTTATATTTCTTCTTGTTTATAATGGTTTTCTTGGGGTTTATTTTGTCTCTAACTCGTTTTCTTAGTTGTTTGATTATTTTAGAGAACTTTAAAGTGTTATTGCTATTATTCAGTTTATTACTTGGCTTACTTGATAGACATGTTTTATTTATTGCGTTGATGGTTGTTTCAACTGTTGAAGTTATTGTTGGTCTTGTTGTTTTAACGCGGGTATGAGAGTGTACAAATTCATTGGATTTGGTTTCTTTTTAGGCTTTTCTTTACTGTTTGTTATTCCTTTGTTGTATTCAGTCGGTGTTGGTGTACTTGGTTCTGTTACTGTTGGTAAAGGTTATTTTATATTTGATTCTGTCTCTTTTTATTTGATTTTATTAATATTCTTTCTGGGGGTATATAGACTTTTCTCTACCTTTTGTCG

>GD_GZ

GTTGGTGCTGTAGCTTTTATGTTTATTTATAAGCTATTTTGATCGAGTTATACTAAGTTGGGTTTTAGTATTTTTTTAGGGTAAATATATGGTTTTTTTATATTTCTTCTTGTTTATAATGGTTTTCTTGGGGTTTATTTTGTCTCTAACTCGTTTTCTTAGTTGTTTGATTATTTTAGAGAACTTTAAAGTGTTATTGCTATTATTCAGTTTATTACTTGGCTTACTTGATAGACATGTTTTATTTATTGCGTTGATGGTTGTTTCAACTGTTGAAGTTATTGTTGGTCTTGTTGTTTTAACGCGGGTATGAGAGTGTACAAATTCATTGGATTTGGTTTCTTTTTAGGCTTTTCTTTACTGTTTGTTATTCCTTTGTTGTATTCAGTCGGTGTTGGTGTACTTGGTTCTGTTACTGTTGGTAAAGGTTATTTTATATTTGATTCTGTCTCTTTTTATTTGATTTTATTAATATTCTTTCTGGGGGTATATAGACTTTTCTCTACCTTTTGTCG

>GD_JM

GTTGGTGCTGTAGCTTTTATGTTTATTTATAAGCTATTTTGATCGAGTTATACTAAGTTGGGTTTTAGTATTTTTTTAGGATAAGTATATGGTTTTTTTATATTTCTTCTTGTTTATAATGGTTTTCTTGGGGTTTATTTTGTCTCTAACTCGTTTTCTTAGTTGTTTGATTATTTTAGAGAACTTTAAAGTGTTATTGCTATTATTCAGTTTATTACTTGGCTTATTTGATAGACATGTTTTATTTATTGCGTTGATGGTTGTTTCAACTGTTGAAGTTATTGTTGGTCTTGTTGTTTTAACACGGGTATGAGAGTGTACAAATTCATTGGATTTGGTTTCTTTTTAGGCTTTTCTTTACTGTTTGTTATTCCCTTATTGTATTCAGTCGGTGTTGGTGTACTTGGTTCTGTTACTGTTGGTAAAGGTTATTTTATATTTGATTCTGTTTCTTTTTATTTGATTTTATTAATATTCTTTCTGGGGGTGTATAGACTTTTCTCTACCTTTTGTCG

>GD_FS

GTTGGTGCTGTAGCTTTTATGTTTATTTATAAGCTATTTTGATCGAGTTATACTAAGTTGGGTTTTAGTATTTTTTTAGGATAAGTATATGGTTTTTTTATATTTCTTCTTGTTTATAATGGTTTTCTTGGGGTTTATTTTGTCTCTAACTCGTTTTCTTAGTTGTTTGATTATTTTAGAGAACTTTAAAGTGTTATTGCTATTATTCAGTTTATTACTTGGCTTATTTGATAGACATGTTTTATTTATTGCGTTGATGGTTGTTTCAACTGTTGAAGTTATTGTTGGTCTTGTTGTTTTAACACGGGTATGAGAGTGTACAAATTCATTGGATTTGGTTTCTTTTTAGGCTTTTCTTTACTGTTTGTTATTCCCTTATTGTATTCAGTCGGTGTTGGTGTACTTGGTTCTGTTACTGTTGGTAAAGGTTATTTTATATTTGATTCTGTTTCTTTTTATTTGATTTTATTAATATTCTTTCTGGGGGTGTATAGACTTTTCTCTACCTTTTGTCG

>GX_WZ

GTTGGTGCTGTAGCTTTTATGTTTATTTATAAGCTATTTTGATCGAGTTATACTAAGTTGGGTTTTAGTATTTTTTTAGGATAAGTATATGGTTTTTTTATATTTCTTCTTGTTTATAATGGTTTTCTTGGGGTTTATTTTGTCTCTAACTCGTTTTCTTAGTTGTTTGATTATTTTAGAGAACTTTAAAGTGTTATTGCTATTATTCAGTTTATTACTTGGCTTATTTGATAGACATGTTTTATTTATTGCGTTGATGGTTGTTTCAACTGTTGAAGTTATTGTTGGTCTTGTTGTTTTAACACGGGTATGAGAGTGTACAAATTCATTGGATTTGGTTTCTTTTTAGGCTTTTCTTTACTGTTTGTTATTCCCTTATTGTATTCAGTCGGTGTTGGTGTACTTGGTTCTGTTACTGTTGGTAAAGGTTATTTTATATTTGATTCTGTTTCTTTTTATTTGATTTTATTAATATTCTTTCTGGGGGTGTATAGACTTTTCTCTACCTTTTGTCG

>GX_YL

GTTGGTGCTGTAGCTTTTATGTTTATTTATAAGCTATTTTGATCGAGTTATACTAAGTTGGGTTTTAGTATTTTTTTAGGATAAGTATATGGTTTTTTTATATTTCTTCTTGTTTATAATGGTTTTCTTGGGGTTTATTTTGTCTCTAACTCGTTTTCTTAGTTGTTTGATTATTTTAGAGAACTTTAAAGTGTTATTGCTATTATTCAGTTTATTACTTGGCTTATTTGATAGACATGTTTTATTTATTGCGTTGATGGTTGTTTCAACTGTTGAAGTTATTGTTGGTCTTGTTGTTTTAACACGGGTATGAGAGTGTACAAATTCATTGGATTTGGTTTCTTTTTAGGCTTTTCTTTACTGTTTGTTATTCCCTTATTGTATTCAGTCGGTGTTGGTGTACTTGGTTCTGTTACTGTTGGTAAAGGTTATTTTATATTTGATTCTGTTTCTTTTTATTTGATTTTATTAATATTCTTTCTGGGGGTGTATAGACTTTTCTCTACCTTTTGTCG

>GX_NN

GTTGGTGCTGTAGCTTTTATGTTTATTTATAAGCTATTTTGATCGAGTTATACTAAGTTGGGTTTTAGTATTTTTTTAGGATAAGTATATGGTTTTTTTATATTTCTTCTTGTTTATAATGGTTTTCTTGGGGTTTATTTTGTCTCTAACTCGTTTTCTTAGTTGTTTGATTATTTTAGAGAACTTTAAAGTGTTATTGCTATTATTCAGTTTATTACTTGGCTTATTTGATAGACATGTTTTATTTATTGCGTTGATGGTTGTTTCAACTGTTGAAGTTATTGTTGGTCTTGTTGTTTTAACACGGGTATGAGAGTGTACAAATTCATTGGATTTGGTTTCTTTTTAGGCTTTTCTTTACTGTTTGTTATTCCCTTATTGTATTCAGTCGGTGTTGGTGTACTTGGTTCTGTTACTGTTGGTAAAGGTTATTTTATATTTGATTCTGTTTCTTTTTATTTGATTTTATTAATATTCTTTCTGGGGGTGTATAGACTTTTCTCTACCTTTTGTCG

>GX_GL

GTTGGTGCTGTAGCTTTTATGTTTATTTATAAGCTATTTTGATCGAGTTATACTAAGTTGGGTTTTAGTATTTTTTTAGGATAAGTATATGGTTTTTTTATATTTCTTCTTGTTTATAATGGTTTTCTTGGGGTTTATTTTGTCTCTAACTCGTTTTCTTAGTTGTTTGATTATTTTAGAGAACTTTAAAGTGTTATTGCTATTATTCAGTTTATTACTTGGCTTATTTGATAGACATGTTTTATTTATTGCGTTGATGGTTGTTTCAACTGTTGAAGTTATTGTTGGTCTTGTTGTTTTAACACGGGTATGAGAGTGTACAAATTCATTGGATTTGGTTTCTTTTTAGGCTTTTCTTTACTGTTTGTTATTCCCTTATTGTATTCAGTCGGTGTTGGTGTACTTGGTTCTGTTACTGTTGGTAAAGGTTATTTTATATTTGATTCTGTTTCTTTTTATTTGATTTTATTAATATTCTTTCTGGGGGTGTATAGACTTTTCTCTACCTTTTGTCG

>HaN_HK

GTTGGTGCTGTAGCTTTTATGTTTATTTATAAGCTATTTTGATCGAGTTATACTAAGTTGGGTTTTAGTATTTTTTTAGGATAAATATATGGTTTTTTTATATTTCTTCTTGTTTATAATGGTTTTCTTGGGGTTTATTTTGTCTCTAACTCGTTTTCTTAGTTGTTTGATTATTTTAGAGAACTTTAAAGTGTTATTGCTATTATTCAGTTTATTACTTGGCTTATTTGATAGACATGTTTTATTTATTGCGTTGATGGTTGTTTCAACTGTTGAAGTTATTGTTGGTCTTGTTGTTTTAACACGGGTATGAGAGTGTACAAATTCATTGGATTTGGTTTCTTTTTAGGCTTTTCTTTACTGTTTGTTATTCCCTTATTGTATTCAGTCGGTGTTGGTGTACTTGGTTCTGTTACTGTTGGTAAAGGTTATTTTATATTTGATTCTGTTTCTTTTTATTTGATTTTATTAATATTCTTTCTGGGGGTGTATAGACTTTTCTC------------

>HaN_WZS

GTTGGTGCTGTAGCTTTTATGTTTATTTATAAGCTATTTTGATCGAGTTATACTAAGTTGGGTTTTAGTATTTTTTTAGGATAAATATATGGTTTTTTTATATTTCTTCTTGTTTATAATGGTTTTCTTGGGGTTTATTTTGTCTCTAACTCGTTTTCTTAGTTGTTTGATTATTTTAGAGAACTTTAAAGTGTTATTGCTATTATTCAGTTTATTACTTGGCTTATTTGATAGACATGTTTTATTTATTGCGTTGATGGTTGTTTCAACTGTTGAAGTTATTGTTGGTCTTGTTGTTTTAACACGGGTATGAGAGTGTACAAATTCATTGGATTTGGTTTCTTTTTAGGCTTTTCTTTACTGTTTGTTATTCCCTTATTGTATTCAGTCGGTGTTGGTGTACTTGGTTCTGTTACTGTTGGTAAAGGTTATTTTATATTTGATTCTGTTTCTTTTTATTTGATTTTATTAATATTCTTTCTGGGGGTGTATAGACTTTTCTC------------

>SC_DZ

GTTGGTGCTGTAGCTTTTATGTTTATTTATAAGCTATTTTGATCGAGTTATACTAAGTTGGGTTTTAGTATTTTTTTAGGATAAGTATATGGTTTTTTTATATTTCTTCTTGTTTATAATGGTTTTCTTGGGGTTTATTTTGTCTCTAACTCGTTTTCTTAGTTGTTTGATTATTTTAGAGAACTTTAAAGTGTTATTGCTATTATTCAGTTTATTACTTGGCTTATTTGATAGACATGTTTTATTTATTGCGTTGATGGTTGTTTCAACTGTTGAAGTTATTGTTGGTCTTGTTGTTTTAACACGGGTATGAGAGTGTACAAATTCATTGGATTTGGTTTCTTTTTAGGCTTTTCTTTACTGTTTGTTATTCCTTTATTGTATTCAGTCGGTGTTGGTGTACTTGGTTCTGTTACTGTTGGTAAAGGTTATTTTATATTTGATTCTGTTTCTTTTTATTTGATTTTATTAATATTCTTTCTGGGGGTGTATAGACTTTTCTCTACCTTTTGTCG

>SC_NC

GTTGGTGCTGTAGCTTTTATGTTTATTTATAAGCTATTTTGATCGAGTTATACTAAGTTGGGTTTTAGTATTTTTTTAGGATAAGTATATGGTTTTTTTATATTTCTTCTTGTTTATAATGGTTTTCTTGGGGTTTATTTTGTCTCTAACTCGTTTTCTTAGTTGTTTGATTATTTTAGAGAACTTTAAAGTGTTATTGCTATTATTCAGTTTATTACTTGGCTTATTTGATAGACATGTTTTATTTATTGCGTTGATGGTTGTTTCAACTGTTGAAGTTATTGTTGGTCTTGTTGTTTTAACACGGGTATGAGAGTGTACAAATTCATTGGATTTGGTTTCTTTTTAGGCTTTTCTTTACTGTTTGTTATTCCTTTATTGTATTCAGTCGGTGTTGGTGTACTTGGTTCTGTTACTGTTGGTAAAGGTTATTTTATATTTGATTCTGTTTCTTTTTATTTGATTTTATTAATATTCTTTCTGGGGGTATATAGACTTTTCTCTACCTTTTGTCG

>SC_GA

GTTGGTGCTGTAGCTTTTATGTTTATTTATAAGCTATTTTGATCGAGTTATACTAAGTTGGGTTTTAGTATTTTTTTAGGATAAGTATATGGTTTTTTTATATTTCTTCTTGTTTATAATGGTTTTCTTGGGGTTTATTTTGTCTCTAACTCGTTTTCTTAGTTGTTTGATTATTTTAGAGAACTTTAAAGTGTTATTGCTATTATTCAGTTTATTACTTGGCTTATTTGATAGACATGTTTTATTTATTGCGTTGATGGTTGTTTCAACTGTTGAAGTTATTGTTGGTCTTGTTGTTTTAACACGGGTATGAGAGTGTACAAATTCATTGGATTTGGTTTCTTTTTAGGCTTTTCTTTACTGTTTGTTATTCCTTTATTGTATTCAGTCGGTGTTGGTGTACTTGGTTCTGTTACTGTTGGTAAAGGTTATTTTATATTTGATTCTGTTTCTTTTTATTTGATTTTATTAATATTCTTTCTGGGGGTATATAGACTTTTCTCTACCTTTTGTCG

>SC_LZ

GTTGGTGCTGTAGCTTTTATGTTTATTTATAAGCTATTTTGATCGAGTTATACTAAGTTGGGTTTTAGTATTTTTTTAGGGTAAATATATGGTTTTTTTATATTTCTTCTTGTTTATAATGGTTTTCTTGGGGTTTATTTTGTCTCTAACTCGTTTTCTTAGTTGTTTGATTATTTTAGAGAACTTTAAAGTGTTATTGCTATTATTCAGTTTATTACTTGGCTTACTTGATAGACATGTTTTATTTATTGCGTTGATGGTTGTTTCAACTGTTGAAGTTATTGTTGGTCTTGTTGTTTTAACGCGGGTATGAGAGTGTACAAATTCATTGGATTTGGTTTCTTTTTAGGCTTTTCTTTACTGTTTGTTATTCCTTTGTTGTATTCAGTCGGTGTTGGTGTACTTGGTTCTGTTACTGTTGGTAAAGGTTATTTTATATTTGATTCTGTCTCTTTTTATTTGATTTTATTAATATTCTTTCTGGGGGTATATAGACTTTTCTCTACCTTTTGTCG

>SC_LSZ

GTTGGTGCTGTAGCTTTTATGTTTATTTATAAGCTATTTTGATCGAGTTATACTAAGTTGGGTTTTAGTATTTTTTTAGGGTAAATATATGGTTTTTTTATATTTCTTCTTGTTTATAATGGTTTTCTTGGGGTTTATTTTGTCTCTAACTCGTTTTCTTAGTTGTTTGATTATTTTAGAGAACTTTAAAGTGTTATTGCTATTATTCAGTTTATTACTTGGCTTACTTGATAGACATGTTTTATTTATTGCGTTGATGGTTGTTTCAACTGTTGAAGTTATTGTTGGTCTTGTTGTTTTAACGCGGGTATGAGAGTGTACAAATTCATTGGATTTGGTTTCTTTTTAGGCTTTTCTTTACTGTTTGTTATTCCTTTGTTGTATTCAGTCGGTGTTGGTGTACTTGGTTCTGTTACTGTTGGTAAAGGTTATTTTATATTTGATTCTGTTTCTTTTTATTTGATTTTATTAATATTCTTTCTGGGGGTATATAGACTTTTCTCTACCTTTTGTCG

>SC_LS

GTTGGTGCTGTAGCTTTTATGTTTATTTATAAGCTATTTTGATCGAGTTATACTAAGTTGGGTTTTAGTATTTTTTTAGGGTAAATATATGGTTTTTTTATATTTCTTCTTGTTTATAATGGTTTTCTTGGGGTTTATTTTGTCTCTAACTCGTTTTCTTAGTTGTTTGATTATTTTAGAGAACTTTAAAGTGTTATTGCTATTATTCAGTTTATTACTTGGCTTACTTGATAGACATGTTTTATTTATTGCGTTGATGGTTGTTTCAACTGTTGAAGTTATTGTTGGTCTTGTTGTTTTAACGCGGGTATGAGAGTGTACAAATTCATTGGATTTGGTTTCTTTTTAGGCTTTTCTTTACTGTTTGTTATTCCTTTGTTGTATTCAGTCGGTGTTGGTGTACTTGGTTCTGTTACTGTTGGTAAAGGTTATTTTATATTTGATTCTGTTTCTTTTTATTTGATTTTATTAATATTCTTTCTGGGAGTATATAGACTTTTCTCTACCTTTTGTCG

>SC_ZG

GTTGGTGCTGTAGCTTTTATGTTTATTTATAAGCTATTTTGATCGAGTTATACTAAGTTGGGTTTTAGTATTTTTTTAGGGTAAATATATGGTTTTTTTATATTTCTTCTTGTTTATAATGGTTTTCTTGGGGTTTATTTTGTCTCTAACTCGTTTTCTTAGTTGTTTGATTATTTTAGAGAACTTTAAAGTGTTATTGCTATTATTCAGTTTATTACTTGGCTTACTTGATAGACATGTTTTATTTATTGCGTTGATGGTTGTTTCAACTGTTGAAGTTATTGTTGGTCTTGTTGTTTTAACGCGGGTATGAGAGTGTACAAATTCATTGGATTTGGTTTCTTTTTAGGCTTTTCTTTACTGTTTGTTATTCCTTTGTTGTATTCAGTCGGTGTTGGTGTACTTGGTTCTGTTACTGTTGGTAAAGGTTATTTTATATTTGATTCTGTCTCTTTTTATTTGATTTTATTAATATTCTTTCTGGGGGTATATAGACTTTTCTCTACCTTTTGTCG

>YN_KM

GTTGGTGCTGTAGCTTTTATGTTTATTTATAAGCTATTTTGATCGAGTTATACTAAGTTGGGTTTTAGTATTTTTTTAGGATAAGTATATGGTTTTTTTATATTTCTTCTTGTTTATAATGGTTTTCTTGGGGTTTATTTTGTCTCTAACTCGTTTTCTTAGTTGTTTGATTATTTTAGAGAACTTTAAAGTGTTATTGCTATTATTCAGTTTATTACTTGGCTTATTTGATAGACATGTTTTATTTATTGCGTTGATGGTTGTTTCAACTGTTGAAGTTATTGTTGGTCTTGTTGTTTTAACACGGGTATGAGAGTGTACAAATTCATTGGATTTGGTTTCTTTTTAGGCTTTTCTTTACTGTTTGTTATTCCCTTATTGTATTCAGTCGGTGTTGGTGTACTTGGTTCTGTTACTGTTGGTAAAGGTTATTTTATATTTGATTCTGTTTCTTTTTATTTGATTTTATTAATATTCTTTCTGGGGGTGTATAGACTTTTCTCTACCTTTTGTCG

>YN_BS

GTTGGTGCTGTAGCTTTTATGTTTATTTATAAGCTATTTTGATCGAGTTATACTAAGTTGGGTTTTAGTATTTTTTTAGGATAAATATATGGTTTTTTTATATTTCTTCTTGTTTATAATGGTTTTCTTGGGGTTTATTTTGTCTCTAACTCGTTTTCTTAGTTGTTTAATTATTTTAGAGAACTTTAAAGTGTTATTGCTATTATTCAGTTTATTACTTGGCTTATTTGATAGACATGTTTTATTTATTGCGTTGATGGTTGTTTCAACTGTTGAAGTTATTGTTGGTCTTGTTGTTTTAACACGGGTATGAGAGTGTACAAATTCATTGGATTTGGTTTCTTTTTAGGCTTTTCTTTACTGTTTGTTATTCCCTTATTGTATTCAGTCGGTGTTGGTGTACTTGGTTCTGTTACTGTTGGTAAAGGTTATTTTATATTTGATTCTGTTTCTTTTTATTTGATTTTATTAATATTCTTTCTGGGGGTGTATAGACTTTTCTCTACCTTTTGTCG

>YN_DHZ

GTTGGTGCTGTAGCTTTTATGTTTATTTATAAGCTATTTTGATCGAGTTATACTAAGTTGGGTTTTAGTATTTTTTTAGGATAAATATATGGTTTTTTTATATTTCTTCTTGTTTATAATGGTTTTCTTGGGGTTTATTTTGTCTCTAACTCGTTTTCTTAGTTGTTTGATTATTTTAGAGAACTTTATAGTGTTATTGCTATTATTCAGTTTATTACTTGGCTTATTTGATAGACATGTTTTATTTATTGCGTTGATGGTTGTTTCAACTGTTGAAGTTATTGTTGGTCTTGTTGTTTTAACACGGGTATGAGAGTGTACAAATTCATTGGATTTGGTTTCTTTTTAGGCTTTTCTTTACTGTTTGTTATTCCCTTATTGTATTCAGTCGGTGTTGGTGTACTTGGTTCTGTTACTGTTGGTAAAGGTTATTTTATATTTGATTCTGTTTCTTTTTATTTGATTTTATTAATATTCTTTCTGGGGGTGTATAGACTTTTCTCTACCTTTTGTCG

>YN_WS

GTTGGTGCTGTAGCTTTTATGTTTATTTATAAGCTATTTTGATCGAGTTATACTAAGTTGGGTTTTAGTATTTTTTTAGGATAAATATATGGTTTTTTTATATTTCTTCTTGTTTATAATGGTTTTCTTGGGGTTTATTTTGTCTCTAACTCGTTTTCTTAGTTGTTTGATTATTTTAGAGAACTTTAAAGTGTTATTGCTATTATTCAGTTTATTACTTGGCTTATTTGATAGACATGTTTTATTTATTGCGTTGATGGTTGTTTCAACTGTTGAAGTTATTGTTGGTCTTGTTGTTTTAACACGGGTATGAGAGTGTACAAATTCATTGGATTTGGTTTCTTTTTAGGCTTTTCTTTACTGTTTGTTATTCCCTTATTGTATTCAGTCGGTGTTGGTGTACTTGGTTCTGTTACTGTTGGTAAAGGTTATTTTATATTTGATTCTGTTTCTTTTTATTTGATTTTATTAATATTCTTTCTGGGGGTGTATAGACTTTTCTCTACCTTTTGTCG

>YN_HH

GTTGGTGCTGTAGCTTTTATGTTTATTTATAAGCTATTTTGATCGAGTTATACTAAGTTGGGTTTTAGTATTTTTTTAGGATAAATATATGGTTTTTTTATATTTCTTCTTGTTTATAATGGTTTTCTTGGGGTTTATTTTGTCTCTAACTCGTTTTCTTAGTTGTTTGATTATTTTAGAGAACTTTAAAGTGTTATTGCTATTATTCAGTTTATTACTTGGCTTATTTGATAGACATGTTTTATTTATTGCGTTGATGGTTGTTTCAACTGTTGAAGTTATTGTTGGTCTTGTTGTTTTAACACGGGTATGAGAGTGTACAAATTCATTGGATTTGGTTTCTTTTTAGGCTTTTCTTTACTGTTTGTTATTCCCTTATTGTATTCAGTCGGTGTTGGTGTACTTGGTTCTGTTACTGTTGGTAAAGGTTATTTTATATTTGATTCTGTTTCTTTTTATTTGATTTTATTAATATTCTTTCTGGGGGTGTATAGACTTTTCTCTACCTTTTGTCG

>GZ_ZY

GTTGGTGCTGTAGCTTTTATGTTTATTTATAAGCTATTTTGATCGAGTTATACTAAGTTGGGTTTTAGTATTTTTTTAGGATAAGTATATGGTTTTTTTATATTTCTTCTTGTTTATAATGGTTTTCTTGGGGTTTATTTTGTCTCTAACTCGTTTTCTTAGTTGTTTGATTATTTTAGAGAACTTTAAAGTGTTATTGCTATTATTCAGTTTATTACTTGGCTTATTTGATAGACATGTTTTATTTATTGCGTTGATGGTTGTTTCAACTGTTGAAGTTATTGTTGGTCTTGTTGTTTTAACACGGGTATGAGAGTGTACAAATTCATTGGATTTGGTTTCTTTTTAGGCTTTTCTTTACTGTTTGTTATTCCCTTATTGTATTCAGTCGGTGTTGGTGTACTTGGTTCTGTTACTGTTGGTAAAGGTTATTTTATATTTGATTCTGTTTCTTTTTATTTGATTTTATTAATATTCTTTCTGGGGGTATATAGACTTTTCTCTACCTTTTGTCG

>GZ_GY

GTTGGTGCTGTAGCTTTTATGTTTATTTATAAGCTATTTTGATCGAGTTATACTAAGTTGGGTTTTAGTATTTTTTTAGGATAAGTATATGGTTTTTTTATATTTCTTCTTGTTTATAATGGTTTTCTTGGGGTTTATTTTGTCTCTAACTCGTTTTCTTAGTTGTTTGATTATTTTAGAGAACTTTAAAGTGTTATTGCTATTATTCAGTTTATTACTTGGCTTATTTGATAGACATGTTTTATTTATTGCGTTGATGGTTGTTTCAACTGTTGAAGTTATTGTTGGTCTTGTTGTTTTAACACGGGTATGAGAGTGTACAAATTCATTGGATTTGGTTTCTTTTTAGGCTTTTCTTTACTGTTTGTTATTCCCTTATTGTATTCAGTCGGTGTTGGTGTACTTGGTTCTGTTACTGTTGGTAAAGGTTATTTTATATTTGATTCTGTTTCTTTTTATTTGATTTTATTAATATTCTTTCTGGGGGTGTATAGACTTTTCTCTACCTTTTGTCG

>GZ_KL

GTTGGTGCTGTAGCTTTTATGTTTATTTATAAGCTATTTTGATCGAGTTATACTAAGTTGGGTTTTAGTATTTTTTTAGGGTAAATATATGGTTTTTTTATATTTCTTCTTGTTTATAATGGTTTTCTTGGGGTTTGTTTTGTCTCTAACTCGTTTTCTTAGTTGTTTGATTATTTTAGAGAACTTTAAAGTGTTATTGCTATTATTCAGTTTATTACTTGGCTTACTTGATAGACATGTTTTATTTATTGCGTTGATGGTTGTTTCAACTGTTGAAGTTATTGTTGGTCTTGTTGTTTTAACGCGGGTATGAGAGTGTACAAATTCATTGGATTTGGTTTCTTTTTAGGCTTTTCTTTACTGTTTGTTATTCCTTTGTTGTATTCAGTCGGTGTTGGTGTACTTGGTTCTGTTACTGTTGGTAAAGGTTATTTTATATTTGATTCTGTCTCTTTTTATTTGATTTTATTAATATTCTTTCTGGGGGTGTATAGACTTTTCTCTACCTTTTGTCG

>GZ_AS

GTTGGTGCTGTAGCTTTTATGTTTATTTATAAGCTATTTTGATCGAGTTATACTAAGTTGGGTTTTAGTATTTTTTTAGGGTAAATATATGGTTTTTTTATATTTCTTCTTGTTTATAATGGTTTTCTTGGGGTTTGTTTTGTCTCTAACTCGTTTTCTTAGTTGTTTGATTATTTTAGAGAACTTTAAAGTGTTATTGCTATTATTCAGTTTATTACTTGGCTTACTTGATAGACATGTTTTATTTATTGCGTTGATGGTTGTTTCAACTGTTGAAGTTATTGTTGGTCTTGTTGTTTTAACGCGGGTATGAGAGTGTACAAATTCATTGGATTTGGTTTCTTTTTAGGCTTTTCTTTACTGTTTGTTATTCCTTTGTTGTATTCAGTCGGTGTTGGTGTACTTGGTTCTGTTACTGTTGGTAAAGGTTATTTTATATTTGATTCTGTCTCTTTTTATTTGATTTTATTAATATTCTTTCTGGGGGTGTATAGACTTTTCTCTACCTTTTGTCG

>CQ_LP

GTTGGTGCTGTAGCTTTTATGTTTATTTATAAGCTATTTTGATCGAGTTATACTAAGTTGGGTTTTAGTATTTTTTTAGGGTAAATATATGGTTTTTTTATATTTCTTCTTGTTTATAATGGTTTTCTTGGGGTTTATTTTGTCTCTAACTCGTTTTCTTAGTTGTTTGATTATTTTAGAGAACTTTAAAGTGTTATTGCTATTATTCAGTTTATTACTTGGCTTACTTGATAGACATGTTTTATTTATTGCGTTGATGGTTGTTTCAACTGTTGAAGTTATTGTTGGTCTTGTTGTTTTAACGCGGGTATGAGAGTGTACAAATTCATTGGATTTGGTTTCTTTTTAGGCTTTTCTTTACTGTTTGTTATTCCTTTGTTGTATTCAGTCGGTGTTGGTGTACTTGGTTCTGTTACTGTTGGTAAAGGTTATTTTATATTTGATTCTGTCTCTTTTTATTTGATTTTATTAATATTCTTTCTGGGGGTATATAGACTTTTCTCTACCTTTTGTCG

>CQ_Z

GTTGGTGCTGTAGCTTTTATGTTTATTTATAAGCTATTTTGATCGAGTTATACTAAGTTGGGTTTTAGTATTTTTTTAGGGTAAATATATGGTTTTTTTATATTTCTTCTTGTTTATAATGGTTTTCTTGGGGTTTATTTTGTCTCTAACTCGTTTTCTTAGTTGTTTGATTATTTTAGAGAACTTTAAAGTGTTATTGCTATTATTCAGTTTATTACTTGGCTTACTTGATAGACATGTTTTATTTATTGCGTTGATGGTTGTTTCAACTGTTGAAGTTATTGTTGGTCTTGTTGTTTTAACGCGGGTATGAGAGTGTACAAATTCATTGGATTTGGTTTCTTTTTAGGCTTTTCTTTACTGTTTGTTATTCCTTTGTTGTATTCAGTCGGTGTTGGTGTACTTGGTTCTGTTACTGTTGGTAAAGGTTATTTTATATTTGATTCTGTCTCTTTTTATTTGATTTTATTAATATTCTTTCTGGGGGTATATAGACTTTTCTCTACCTTTTGTCG

>CQ_FL

GTTGGTGCTGTAGCTTTTATGTTTATTTATAAGCTATTTTGATCGAGTTATACTAAGTTGGGTTTTAGTATTTTTTTAGGGTAAATATATGGTTTTTTTATATTTCTTCTTGTTTATAATGGTTTTCTTGGGGTTTATTTTGTCTCTAACTCGTTTTCTTAGTTGTTTGATTATTTTAGAGAACTTTAAAGTGTTATTGCTATTATTCAGTTTATTACTTGGCTTACTTGATAGACATGTTTTATTTATTGCGTTGATGGTTGTTTCAACTGTTGAAGTTATTGTTGGTCTTGTTGTTTTAACGCGGGTATGAGAGTGTACAAATTCATTGGATTTGGTTTCTTTTTAGGCTTTTCTTTACTGTTTGTTATTCCTTTGTTGTATTCAGTCGGTGTTGGTGTACTTGGTTCTGTTACTGTTGGTAAAGGTTATTTTATATTTGATTCTGTTTCTTTTTATTTGATTTTATTAATATTCTTTCTGGGGGTATATAGACTTTTCTCTACCTTTTGTCG

>CQ_YuY

GTTGGTGCTGTAGCTTTTATGTTTATTTATAAGCTATTTTGATCGAGTTATACTAAGTTGGGTTTTAGTATTTTTTTAGGGTAAATATATGGTTTTTTTATATTTCTTCTTGTTTATAATGGTTTTCTTGGGGTTTATTTTGTCTCTAACTCGTTTTCTTAGTTGTTTGATTATTTTAGAGAACTTTAAAGTGTTATTGCTATTATTCAGTTTATTACTTGGCTTACTTGATAGACATGTTTTATTTATTGCGTTGATGGTTGTTTCAACTGTTGAAGTTATTGTTGGTCTTGTTGTTTTAACGCGGGTATGAGAGTGTACAAATTCATTGGATTTGGTTTCTTTTTAGGCTTTTCTTTACTGTTTGTTATTCCTTTGTTGTATTCAGTCGGTGTTGGTGTACTTGGTTCTGTTACTGTTGGTAAAGGTTATTTTATATTTGATTCTGTCTCTTTTTATTTGATTTTATTAATATTCTTTCTGGGGGTATATAGACTTTTCTCTACCTTTTGTCG

>CQ_YoY

GTTGGTGCTGTAGCTTTTATGTTTATTTATAAGCTATTTTGATCGAGTTATACTAAGTTGGGTTTTAGTATTTTTTTAGGATAAGTATATGGTTTTTTTATATTTCTTCTTGTTTATAATGGTTTTCTTGGGGTTTATTTTGTCTCTAACTCGTTTTCTTAGTTGTTTGATTATTTTAGAGAACTTTAAAGTGTTATTGCTATTATTCAGTTTATTACTTGGCTTATTTGATAGACATGTTTTATTTATTGCGTTGATGGTTGTTTCAACTGTTGAAGTTATTGTTGGTCTTGTTGTTTTAACACGGGTATGAGAGTGTACAAATTCATTGGATTTGGTTTCTTTTTAGGCTTTTCTTTACTGTTTGTTATTCCCTTATTGTATTCAGTCGGTGTTGGTGTACTTGGTTCTGTTACTGTTGGTAAAGGTTATTTTATATTTGATTCTGTCTCTTTTTATTTGATTTTATTAATATTCTTTCTGGGGGTGTATAGACTTTTCTCTACCTTTTGTCG
